# Supplementary material for: Effectiveness and acceptability of cognitive–behavioural therapy based interventions for maternal peripartum depression: a systematic review, meta-analysis and thematic synthesis protocol
Source: BMJ Open. 2019 Dec 22;9(12):e032659. doi: 10.1136/bmjopen-2019-032659 (PMC6937015; doi:10.1136/bmjopen-2019-032659)
Supplement: Supplementary data [file bmjopen-2019-032659supp002.pdf]

PICOS STATEMENT

Table 1: PICOS statement

PICOS	Eligibility Criteria	✓
Population	Adult women (aged ≥ 16 years)	❑
	Antepartum or postpartum (up to 12 months)	❑
	Depressive disorder with peripartum onset (no limits will be placed on depression severity)<br><br>For example:<br>• A diagnosis of major depression with peripartum onset specifier in accordance with the Diagnostic and Statistical Manual of Mental Disorders (DSM) IV or V.[37,38]<br><br>• Reporting depression symptomatology with a peripartum onset using a validated tool e.g., Edinburgh Postnatal Depression Scale (EPDS).[39]	❑
	Exclusion criteria: intervention for mood disorders other than depression (e.g. bipolar affective disorder).	❑ Does not contain
	Exclusion criteria: intervention explicitly targeting the prevention of symptoms of depression in at-risk mothers in the perinatal period.<br><br>For example,<br>A study to treating current depression during the antepartum period, with an aim to prevent depression during the postpartum period would be eligible for inclusion. However, interventions explicitly targeting the prevention of depression during either the antepartum or postpartum period will be excluded.	❑ Does not contain
Intervention	Interventions explicitly targeting an improvement in peripartum depression e.g., not targeting mother-infant interaction.<br><br>*Note:* no exclusions are placed upon:<br>• Professional group supporting the intervention<br>• Clinical setting of the intervention<br>• Delivery mode (self-guided, individual or group)<br>• Support methods (internet, face-to-face or telephone)	❑
	Intervention explicitly states the use of cognitive behavioural therapy (CBT), behavioural activation (BA) and/or problem-solving.<br><br>For example:<br><br>• CBT will be defined as interventions in which the focus is modifying a client's dysfunctional thoughts on current behaviour and future functioning.[13]	❑

	• BA will be defined as interventions targeting reductions in behavioural avoidance and increases in positively reinforcing activities, including interventions that focus on scheduling behaviours.[40]<br>• Problem-solving interventions will be defined as a psychological intervention including the following elements: definition of personal problems, generation of multiple solutions to each problem, selection of the best solution, developing a systematic plan for this solution, and evaluating whether the solution has resolved the problem. [13]	
Comparison	Trial contains a suitable control condition<br><br>For example:<br><br>• no-treatment control<br>• wait-list control<br>• treatment-as-usual<br>• non-specific factors component control<br>• specific factors component control<br>• active comparator<br><br>*Note:* only designs that isolate the effects of CBT should be included. For example, a study comparing CBT alone versus medication alone would be excluded as it would not be possible to isolate the effect of the CBT.	❑
Outcomes	Depression or peripartum depression is the primary outcome measure (self-report, clinician or proxy administered measure of depression)	❑
	Quality of depression measure used has internal consistency and test-retest reliability with a Cronbach's alpha ≥0.70	❑
Study design	Only randomized controlled trials	❑
	Quality of randomization procedure based on Risk of Bias tool 2.0. [57]<br><br>*Exclusion criteria:* Non-random sequence generation (high risk of bias)<br><br>*Exclusion criteria:* Designs in which allocation sequence is not concealed (before participants are enrolled and assigned to interventions) (high risk of bias)	❑<br>❑
Overall decision<br><br>Include ❑ Exclude ❑ Review ❑		
Notes:		

Table 2: Article allocation in Endnote

# Endnote	Allocation	Description
0	Include	Article in line with PICOS or does not detail enough information to warrant exclusion
1	Exclude	Article clearly not in line with PICOS
2	Systematic review	Article is a systematic review in line with the PICOS
3	Meta-analysis	Article is a meta-analysis in line with the PICOS
4	Review	Article is a review of relevant literature
5	Protocol	Article is a study protocol in line with PICOS
6	Qualitative	Article is a qualitative paper in line with PICOS
7	Other	Article is useful to researcher for another purpose
