# Supplementary material for: Effectiveness and acceptability of cognitive–behavioural therapy based interventions for maternal peripartum depression: a systematic review, meta-analysis and thematic synthesis protocol
Source: BMJ Open. 2019 Dec 22;9(12):e032659. doi: 10.1136/bmjopen-2019-032659 (PMC6937015; doi:10.1136/bmjopen-2019-032659)
Supplement: Supplementary data [file bmjopen-2019-032659supp004.pdf]

PRESS Guideline — Search Submission & Peer Review Assessment

SEARCH SUBMISSION: *THIS SECTION TO BE FILLED IN BY THE SEARCHER*

Searcher: Danelle Pettman **Email:** Danelle.pettman@kbh.uu.se
Reviewer: Professor Alkistis Skalkidou
Date submitted: 12.11.2018 **Date requested by**: 19.11.2018

Systematic Review Title:

Effectiveness and acceptability of cognitive behavioural therapy based interventions for maternal peripartum depression: A systematic review, meta-analysis and thematic synthesis.

This search strategy is …

X	My PRIMARY (core) database strategy — First time submitting a strategy for search question and database
	My PRIMARY (core) strategy — Follow-up review NOT the first time submitting a strategy for search question and database. If this is a response to peer review, itemize the changes made to the review suggestions
	SECONDARY search strategy— First time submitting a strategy for search question and database
	SECONDARY search strategy — NOT the first time submitting a strategy for search question and database. If this is a response to peer review, itemize the changes made to the review suggestions

Database

(i.e., MEDLINE,CINAHL…): *[mandatory]*

Medline

Interface

(i.e., Ovid, EBSCO…): *[mandatory]*

Pubmed

Additional database searches

Once the primary search strategy is confirmed we will also search; ISI Web of Science; Cumulative Index to Nursing and Allied Health Literature (CINAHL); Cochrane Central Register of Controlled Trials (CENTRAL); Prospero; Excerpta Medica DataBase (EMBASE); Applied Social Sciences Index and Abstracts (ASSIA); Psychinfo; SCOPUS; and SweMed+

Research Question

(Describe the purpose of the search) *[mandatory]*

(1) to examine the effectiveness of CBT based interventions for PPD on symptoms of depression; (2) to examine the effectiveness of CBT based interventions for PPD on secondary outcome measures including; anxiety, stress (individual and perceived parenting stress), parenting (e.g., sensitivity/responsiveness) perceived social support and perceived parental competence; (3) to investigate clinical and methodological moderators potentially associated with effectiveness; and (4) to describe the acceptability of CBT based interventions for PPD including (but not limited to) mothers' experiences and perceptions, satisfaction, barriers and facilitators to intervention use, intervention relevance to mothers' situations and suggestions for improvements; and (5) to identify adaptations to CBT based interventions associated with acceptability.

PICO Format

(Outline the PICOs for your question — i.e., Patient, Intervention, Comparison, Outcome, and Study Design — as applicable)

**P**	Mothers from conception to 1 year postpartum suffering with depression or low mood
**I**	All variants of behavioural, cognitive and cognitive-behavioural therapy
**C**	All comparators will be considered
**O**	Measures of depression or peripartum depression
**S**	Randomised control trials

Inclusion Criteria

(List criteria such as age groups, study designs, etc., to be included) *[optional]*

Participants. Adult women with either a diagnosis of PDD, or reporting some level of depression symptomatology with a peripartum onset using a validated tool (e.g., Edinburgh Postnatal Depression Scale (EPDS).

Interventions. CBT, behavioural activation (BA) and problem-solving interventions explicitly targeting an improvement in peripartum depression as a primary outcome.

Comparators. (1) no-treatment control; (2) wait-list control; (3) treatment-as-usual (TAU); (4) non-specific factors component control; (5) specific factors component control; and (6) active comparator

Outcomes: Primary outcomes of interest are self-report, clinician or proxy administered standardised measurement of depression or peripartum depession. Secondary outcomes of interest are standardised measures of (1) anxiety; (2) individual stress; (3) perceived parental stress; (4) self-report parenting; (5) perceived social support; and (6) parental competence.

Study design. Only randomized control trials will be included.

Exclusion Criteria

(List criteria such as study designs, date limits, etc., to be excluded) *[optional]*

Participants. (a) the treatment of mood disorders other than depression (e.g. bipolar affective disorder); and (b) interventions focussed on prevention of maternal psychopathology in at-risk, but not currently symptomatic mothers.

Only studies in English or Swedish will be included.

Was a search filter applied?

No ■ Yes ☐

If YES, which one(s) (e.g., Cochrane RCT filter, PubMed Clinical Queries filter)? Provide the source if this is a published filter. *[mandatory if YES to previous question — textbox]*

Pettman D, *et al. BMJ Open* 2019; 9:e032659. doi: 10.1136/bmjopen-2019-032659

Other notes or comments you feel would be useful for the peer reviewer? ***[optional]***

We had considered not including the concept 4 therapy terms, however this reduces the specificity of the search.

Please copy and paste your search strategy here, exactly as run, including the number of hits per line. ***[mandatory]***

Pettman D, *et al. BMJ Open* 2019; 9:e032659. doi: 10.1136/bmjopen-2019-032659

Concept 1	Concept 2	Concept 3	Concept 4	Concept 5
**Perinatal**	**Depression terms**	**CBT**	**Therapy terms**	**RCT**
**Title & Abstract**<br>Postpartum 47725<br>post-partum 11003<br>antepartum 5374<br>ante-partum 423<br>partum 12020<br>prepartum 2077<br>pre-partum 329<br>intrapartum 7905<br>intra-partum 319<br>peripartum 4151<br>peri-partum 160<br>postnatal 96485<br>post-natal 6733<br>Perinatal 65465<br>peri-natal 177<br>antenatal 31533<br>ante-natal 511<br>prenatal 87822<br>pre-natal 1028<br>pregnan* 480609<br>puerper* 14147<br>maternal 231206<br>trimester 49419<br>impregnated 10808<br>gravid* 12698<br>multigravid* 1185<br>primigravid* 4766<br>parity 29634<br>obstetric 39258<br>gestation 109623<br>before birth 3679<br>in utero 25721<br>**Mesh terms**<br>Pregnancy 844017	**Title & Abstract**<br>depress* 416497<br>depressive 102129<br>low mood 621<br>mood 66046<br>distress 99553<br>wellbeing 12032<br>well-being 63718<br>emotion 33746<br>emotional 133450<br>melanchol* 2906<br>affect 589721<br>affective 51447<br>affective symptoms 1647<br>negative affect 8508<br>dysphori*5153<br>affective disorder 6830<br>**Mesh terms**<br>affect 30820<br>mood disorders 112524<br>depression 195007<br>depressive disorder 100595<br>affective symptoms 12245<br>affective disorder 112524	**Title & Abstract**<br>cognitive 306140<br>cognitive behavio* 23129<br>behavio* 1122596<br>CBT 9128<br>BA 25470<br>problem solving 16422<br>ccbt 155<br>icbt 551<br>cognitive restructuring 785<br>cognitive reframing 84<br>activity scheduling 39<br>**Mesh terms**<br>behavior therapy 67509<br>cognitive therapy 24435<br>behavior therapies, cognitive 24435	**Title & Abstract**<br>Therapy 1749047<br>Therapies 248514<br>Psychotherapy 35578<br>Intervention 515758<br>Management 983110<br>program evaluation 6170<br>program 400451<br>programs 255468<br>programme 86763<br>programmes 55208<br>group 2335915<br>course 515471<br>online 86832<br>Internet 45003<br>Web 89875<br>web-based 25325<br>Phone 18507<br>Telephone 52303<br>Skype 273<br>e-therapy 452<br>etherapy 9<br>computer-assisted 24439<br>computerized 67756<br>internet intervention* 462<br>computer* 281642<br>mobile 85365<br>tablet* 48173<br>smartphone* 6968<br>internet-administered 65<br>ementalhealth 26	**Publication type**<br>Randomized controlled trial 470367<br>systematic review 222096<br>Clinical trial 809548<br>controlled clinical trial 558223<br>meta-analysis 93273<br>**Title & Abstract**<br>Randomized controlled trial 57325<br>Randomized control trial 3210<br>RCT 18011<br>Controlled 684318<br>randomi* 557922<br>random assignment 2191<br>random allocation 1516<br>random 239928<br>randomly 299405<br>control 2340336<br>controls 775814<br>feasibility 154608<br>pilot 132357<br>comparative study 72223<br>follow up 852728<br>meta-analysis 116966<br>metaanalysis 1531<br>Review 1392398<br>**Mesh terms**<br>allocation, random 96464<br>randomized controlled trial 121644<br>meta analysis 17054<br>randomized controlled trials as topic 121644<br>meta analysis as topic 17054<br>review, systematic 0

postpartum period 59467<br>prenatal care 24767<br>perinatal care 8957<br>care, postnatal 5085<br>pregnancy complications 400382<br>depression, postpartum 4780			e-mental health 175<br>m-mental health 3<br>Mmentalhealth 1<br>Ehealth 2629<br>e-health 2356	
**Total for concept 1: 1230102**	**Total for concept 2: 1670493**	**Total for concept 3: 1414869**	**Total for concept 4: 6226286**	**Total for concept 5: 6135718**
				**Total for all: 3917**

Syntax:

(((((((((((((((((((((((((((((((((((((postpartum[Title/Abstract]) OR post-partum antepartum[Title/Abstract]) OR ante-partum partum[Title/Abstract]) OR prepartum[Title/Abstract]) OR pre-partum[Title/Abstract]) OR intrapartum[Title/Abstract]) OR intra-partum[Title/Abstract]) OR peripartum[Title/Abstract]) OR peri-partum[Title/Abstract]) OR postnatal[Title/Abstract]) OR post-natal[Title/Abstract]) OR Perinatal[Title/Abstract]) OR peri-natal[Title/Abstract]) OR antenatal[Title/Abstract]) OR ante-natal[Title/Abstract]) OR prenatal[Title/Abstract]) OR pre-natal[Title/Abstract]) OR pregnan*[Title/Abstract]) OR puerper[Title/Abstract]) OR puerper*[Title/Abstract]) OR maternal[Title/Abstract]) OR trimester[Title/Abstract]) OR impregnated[Title/Abstract]) OR gravid*[Title/Abstract]) OR multigravid*[Title/Abstract]) OR primigravid*[Title/Abstract]) OR parity[Title/Abstract]) OR obstetric[Title/Abstract]) OR gestation[Title/Abstract]) OR before birth[Title/Abstract]) OR in utero[Title/Abstract])) OR (((((((pregnancy[MeSH Terms]) OR postpartum period[MeSH Terms]) OR prenatal care[MeSH Terms]) OR perinatal care[MeSH Terms]) OR care, postnatal[MeSH Terms]) OR pregnancy complications[MeSH Terms]) OR depression, postpartum[MeSH Terms]))) AND (((((((((((((((((((depress*[Title/Abstract]) OR depressive[Title/Abstract]) OR low mood[Title/Abstract]) OR mood[Title/Abstract]) OR distress[Title/Abstract]) OR wellbeing[Title/Abstract]) OR well being[Title/Abstract]) OR emotion[Title/Abstract]) OR emotional[Title/Abstract]) OR melanchol*[Title/Abstract]) OR affect[Title/Abstract]) OR affective[Title/Abstract]) OR affective symptoms[Title/Abstract]) OR negative affect[Title/Abstract]) OR dysphori*[Title/Abstract]) OR affective disorder[Title/Abstract])) OR ((((((affect[MeSH Terms]) OR mood disorders[MeSH Terms]) OR depression[MeSH Terms]) OR depressive disorder[MeSH Terms]) OR affective symptoms[MeSH Terms]) OR affective disorder[MeSH Terms]))) AND (((((((((((((cognitive[Title/Abstract]) OR cognitive behavio*[Title/Abstract]) OR behavio*[Title/Abstract]) OR CBT[Title/Abstract]) OR BA[Title/Abstract]) OR problem solving[Title/Abstract]) OR ccbt[Title/Abstract]) OR icbt[Title/Abstract]) OR cognitive

restructuring[Title/Abstract]) OR cognitive reframing[Title/Abstract]) OR activity scheduling[Title/Abstract])) OR (((cognitive behavior therapy[MeSH Terms]) OR behavior therapy[MeSH Terms]) OR cognitive therapy[MeSH Terms]))) AND (((((((((((((((((((((((((((((((((((therapy[Title/Abstract]) OR therapies[Title/Abstract]) OR psychotherapy[Title/Abstract]) OR intervention[Title/Abstract]) OR management[Title/Abstract]) OR program evaluation[Title/Abstract]) OR program[Title/Abstract]) OR programs[Title/Abstract]) OR programme[Title/Abstract]) OR programmes[Title/Abstract]) OR group[Title/Abstract]) OR course[Title/Abstract]) OR online[Title/Abstract]) OR Internet[Title/Abstract]) OR Web[Title/Abstract]) OR web-based[Title/Abstract]) OR Phone[Title/Abstract]) OR Telephone[Title/Abstract]) OR Skype[Title/Abstract]) OR e-therapy[Title/Abstract]) OR Etherapy[Title/Abstract]) OR computer-assisted[Title/Abstract]) OR computerized[Title/Abstract]) OR internet intervention*[Title/Abstract]) OR computer*[Title/Abstract]) OR mobile[Title/Abstract]) OR tablet*[Title/Abstract]) OR smartphone*[Title/Abstract]) OR internet-administered[Title/Abstract]) OR ementalhealth[Title/Abstract]) OR e-mental health[Title/Abstract]) OR m-mental health[Title/Abstract]) OR Mmentalhealth[Title/Abstract]) OR Ehealth[Title/Abstract]) OR e-health[Title/Abstract])) AND (((((((Randomized controlled trial[Publication Type]) OR systematic review[Publication Type]) OR controlled clinical trial[Publication Type]) OR meta-analysis[Publication Type])) OR ((((((((((((((((((Randomized controlled trial[Title/Abstract]) OR Randomized control trial[Title/Abstract]) OR RCT[Title/Abstract]) OR Controlled[Title/Abstract]) OR randomi*[Title/Abstract]) OR random assignment[Title/Abstract]) OR random allocation[Title/Abstract]) OR random[Title/Abstract]) OR randomly[Title/Abstract]) OR control[Title/Abstract]) OR controls[Title/Abstract]) OR feasibility[Title/Abstract]) OR pilot[Title/Abstract]) OR comparative study[Title/Abstract]) OR follow up[Title/Abstract]) OR meta-analysis[Title/Abstract]) OR metaanalysis[Title/Abstract]) OR Review[Title/Abstract])) OR ((((((allocation, random[MeSH Terms]) OR randomized controlled trial[MeSH Terms]) OR meta analysis[MeSH Terms]) OR randomized controlled trials as topic[MeSH Terms]) OR meta analysis as topic[MeSH Terms]) OR review, systematic[MeSH Terms]))

(Add more space, as necessary.)

Pettman D, *et al. BMJ Open* 2019; 9:e032659. doi: 10.1136/bmjopen-2019-032659

PEER REVIEW ASSESSMENT: *THIS SECTION TO BE FILLED IN BY THE REVIEWER*

Reviewer: Professor Alkistis Skalkidou
Date completed: 12/12/2018

1. TRANSLATION

A ---No revisions	☐
B --- Revision(s) suggested	■
C --- Revision(s) required	☐

If "B" or "C," please provide an explanation or example:

Does the search strategy match the research question/PICO?
Yes
Are the search concepts clear?
Yes
Are there too many or too few PICO elements included?
Adequate- but I wonder if the algorithm will work well when you require CBT AND therapy- will it catch something that only has CBT in the text? The word CBT includes the concept of therapy
Same goes for MESH term depression, postpartum AND depression…
Are the search concepts too narrow or too broad?
Ok but sometimes they include each other, see above- Maybe this is not a problem but should be checked.
Does the search retrieve too many or too few records? (Please show number of hits per line.)
As expected
Are unconventional or complex strategies explained?
Yes

2. BOOLEAN AND PROXIMITY OPERATORS

A ---No revisions	☐
B --- Revision(s) suggested	■
C --- Revision(s) required	☐

If "B" or "C," please provide an explanation or example:

Are Boolean or proximity operators used correctly?
Yes
Is the use of nesting with brackets appropriate and effective for the search?
Yes
If NOT is used, is this likely to result in any unintended exclusions?
-
Could precision be improved by using proximity operators (eg, adjacent, near, within) or phrase searching instead of AND?
Probably, should be checked
Is the width of proximity operators suitable (eg, might adj5 pick up more variants than adj2)?
Not used

3. SUBJECT HEADINGS

A ---No revisions	■
B --- Revision(s) suggested	□
C --- Revision(s) required	□

If "B" or "C," please provide an explanation or example:

Are the subject headings relevant?
Yes
Are any subject headings too broad or too narrow?
No but somethings they intercept
Are subject headings exploded where necessary and vice versa?
Yes
Are major headings ("starring" or restrict to focus) used? If so, is there adequate justification?
-
Are subheadings missing?
No
Are subheadings attached to subject headings? (Floating subheadings may be preferred.)
Are not
Are floating subheadings relevant and used appropriately?
Have not seen any used (Subheading [sh:noexp])
Are both subject headings and terms in free text (see the following) used for each concept?
Yes

4. TEXT WORD SEARCHING

A ---No revisions	☒
B --- Revision(s)suggested	☐
C --- Revision(s) required	☐

If “B” or “C,” please provide an explanation or example:

Does the search include all spelling variants in free text (eg, UK vs. US spelling)?
Yes
Does the search include all synonyms or antonyms (eg, opposites)?
Perinatal concept include: “Maternity”, “Partus” and “Obstetrical”
Depression concept include: “dysthymia” and “alexithymia”
Does the search capture relevant truncation (ie, is truncation at the correct place)?
Yes
Is the truncation too broad or too narrow?
Adequate
Are acronyms or abbreviations used appropriately? Do they capture irrelevant material? Are the full terms also included?
Yes
Have the appropriate fields been searched; for example, is the choice of the text word fields (.tw.) or all fields (.af.) appropriate? Are there any other fields to be included or excluded (database specific)?
OK
Should any long strings be broken into several shorter search statements?

5. SPELLING, SYNTAX, AND LINE NUMBERS

A ---No revisions	☒
B --- Revision(s)suggested	☐
C --- Revision(s) required	☐

If “B” or “C,” please provide an explanation or example:

Are there any spelling errors?
I have not identified any.
Are there any errors in system syntax; for example, the use of a truncation symbol from a different search interface?
No
Are there incorrect line combinations or orphan lines (ie, lines that are not referred to in the final summation that could indicate an error in an AND or OR statement)?
No

6. LIMITS AND FILTERS

A ---No revisions	■
B --- Revision(s) suggested	☐
C --- Revision(s) required	☐

If "B" or "C," please provide an explanation or example:

OVERALL EVALUATION (Note: If one or more "revision required" is noted above, the response below must be "revisions required".)

A ---No revisions	☐
B --- Revision(s) suggested	■
C --- Revision(s) required	☐

Additional comments:

Some more comments- look at my review on ART and endometrial cancer, Cochrane, on how we looked at grey literature (additional databases).
I also thought that you have the whole perinatal period as one, and it could be very different effects of interventions for those depressed during and/or after pregnancy- I do not know if you want to already now plan for sensitivity analyses according to timing of intervention and/or maternal depression. Outcome has the same problem- at which time is depression measured? Just a thought.
