# Supplementary material for: Effectiveness and acceptability of cognitive–behavioural therapy based interventions for maternal peripartum depression: a systematic review, meta-analysis and thematic synthesis protocol
Source: BMJ Open. 2019 Dec 22;9(12):e032659. doi: 10.1136/bmjopen-2019-032659 (PMC6937015; doi:10.1136/bmjopen-2019-032659)
Supplement: Supplementary data [file bmjopen-2019-032659supp005.pdf]

Data Extraction Form

Reviewer	
**Study Identification Features**	
Unique Study Identifier	
Title	
Authors	
Year of publication	
Citation	
Publication type	
Journal name	
Linked papers	
Contact author information	
Country of origin	
Funding source	
Language	
**Study Characteristics**	
Aims and objectives	
Design	
Inclusion/exclusion criteria	
**Participant characteristics**	
Method of depression assessment (e.g. structured clinical interview or screening tool)	
Diagnosis of major depressive disorder (yes/no)	
Recruitment setting (clinical or community)	
Age of mother (Mean, SD)	
Age of infant (Mean, SD)	
Point of intervention (e.g., prenatal or postnatal) ***Moderator***	
Ethnicity of mother (n, %)	
Relationship status (n, %)	
Educational status (n, %)	
Employment status (n, %),	
Mothers First child (n, %)	
Average household income (n,%)	
Breastfeeding (n,%)	
Level of depression severity at baseline (if clinical cut offs available)	
Depression Chronicity years (Mean, SD)	

**Intervention Components**	
Type of CBT intervention (CBT, BA or problem solving) ***Moderator***	
Inclusion of social components (e.g., peer support group or involvement of partner) -Yes/No ***Moderator***	
Inclusion of parenting intervention components (e.g., video interaction guidance - Yes/No ***Moderator***	
Treatment manual (yes/no)	
Measurement of treatment adherence (yes/no)	
Method of delivery (e.g. face to face, group or internet-administered) ***Moderator***	
Treatment setting (for example, primary care, inpatient)	
Health professional delivering intervention (e.g., clinical psychologist or midwife) ***Moderator***	
Study specific training (Yes/No)	
Duration of treatment (Weeks)	
Number of sessions	
Length of sessions (Minutes)	
Maximum length of treatment sessions over treatment course (Minutes)	
Group size (Mean, SD)	
Type of control condition used (e.g., no-treatment control, wait-list control, TAU, non- specific factors component control, specific factors component control and active comparator) ***Moderator***	
**Outcome Measurements**	
Primary outcome measure for each time point collected in both intervention and control conditions. Where multiple time points are reported, a primary end point ≤6 months post-treatment will be adopted. However, outcomes for all time points reported will be examined to enable a potential moderator analysis on length of follow-up.	
Depression measure used	
Participant n	
Mean	

SD and/or SE	
Quality of primary outcome measure - Cronbach's alpha for internal consistency (may need original validation paper)	
Quality of primary outcome measure - Cronbach's alpha for test-retest reliability (may need original validation paper)	
Length of follow up categorical (e.g., short; post-treament-3 months, medium; 3-6 months, long; 7-11 months; extended; 12 months+) ***Moderator***	
Secondary outcome measure for each secondary outcome (anxiety, individual stress, perceived parental stress, self-report parenting, perceived social support, parental competence and observational parenting) at each time point collected in both intervention and control conditions.	
Measure used	
Participant n	
Mean	
SD and/or SE	
**Statistical Techniques**	
Power calculation	
Intention to treat (yes/no)	
Method of dealing with missing data	
Baseline comparability	
Estimates of intracluster correlation coefficients (ICC) (cluster trials)	
**Participant Flow**	
Randomised to intervention	
Randomised to control	
Lost to follow-up intervention (at each time point measured)	
Lost to follow-up control (at each time point measured)	
Analysed intervention (at each time point measured)	
Analysed control (at each time point measured)	
Attrition rate	
**Research ethics**	
Data relating to ethics (e.g., ethical approval, ethical issues highlighted)	
**Additional comments**	
