# Supplementary material for: Effectiveness and acceptability of cognitive–behavioural therapy based interventions for maternal peripartum depression: a systematic review, meta-analysis and thematic synthesis protocol
Source: BMJ Open. 2019 Dec 22;9(12):e032659. doi: 10.1136/bmjopen-2019-032659 (PMC6937015; doi:10.1136/bmjopen-2019-032659)
Supplement: Author's manuscript [file bmjopen-2019-032659.draft_revisions.pdf]

BMJ Open is committed to open peer review. As part of this commitment we make the peer review history of every article we publish publicly available.

When an article is published we post the peer reviewers' comments and the authors' responses online. We also post the versions of the paper that were used during peer review. These are the versions that the peer review comments apply to.

The versions of the paper that follow are the versions that were submitted during the peer review process. They are not the versions of record or the final published versions. They should not be cited or distributed as the published version of this manuscript.

BMJ Open is an open access journal and the full, final, typeset and author-corrected version of record of the manuscript is available on our site with no access controls, subscription charges or pay-per-view fees (http://bmjopen.bmj.com).

If you have any questions on BMJ Open's open peer review process please email info.bmjopen@bmj.com

BMJ Open

Effectiveness and acceptability of cognitive behavioural therapy based interventions for maternal peripartum depression: a systematic review, meta-analysis and thematic synthesis protocol.

Journal:	*BMJ Open*
Manuscript ID	bmjopen-2019-032659
Article Type:	Protocol
Date Submitted by the Author:	10-Jul-2019

Complete List of Authors:	Pettman, Danelle; Uppsala University, 751 85 Uppsala, Sweden, Clinical Psychology in Healthcare, Department of Women's and Children's Health<br>O'Mahen, Heather; University of Exeter, Perry Road, Devon, EX4 4QG, United Kingdom, Mood Disorders Centre, School of Psychology<br>Svanberg, Agneta ; Uppsala University, 751 85 Uppsala, Sweden, Reproductive Health, Department of Women's and Children's Health<br>von Essen, Louise; Uppsala University, 751 85 Uppsala, Sweden, Clinical Psychology in Healthcare, Department of Women's and Children's Health<br>Axfors, Cathrine; Uppsala University, 751 85 Uppsala, Sweden, Reproductive Health, Department of Women's and Children's Health,<br>Blomberg, Oscar; Uppsala University, 751 85 Uppsala, Sweden, Clinical Psychology in Healthcare, Department of Women's and Children's Health<br>Woodford, Joanne; Uppsala University, 751 85 Uppsala, Sweden, Clinical Psychology in Healthcare, Department of Women's and Children's Health

Keywords:	Depression & mood disorders < PSYCHIATRY, PERINATOLOGY, PSYCHIATRY

SCHOLARONE™
Manuscripts

Effectiveness and acceptability of cognitive behavioural therapy based interventions for maternal peripartum depression: a systematic review, meta-analysis and thematic synthesis protocol.

Danelle PETTMAN[1] (danelle.pettman@kbh.uu.se), Heather O'MAHEN[2] (H.OMahen@exeter.ac.uk), Agneta SKOOG SVANBERG[3] (Agneta.Skoog_Svanberg@kbh.uu.se), Louise VON ESSEN[1] (louise-von.essen@kbh.uu.se), Cathrine AXFORS[3] (cathrine.axfors@neuro.uu.se), Oscar BLOMBERG[1], (oscar.blomberg@kbh.uu.se), Joanne WOODFORD[1] (joanne.woodford@kbh.uu.se)

Corresponding author

Dr Danelle Pettman, Clinical Psychology in Healthcare, Department of Women's and Children's Health, Uppsala University, 751 23 Uppsala, Sweden. E-mail: danelle.pettman@kbh.uu.se. Telephone: 0046 18-471 62 58

Affiliations

[1]Clinical Psychology in Healthcare, Department of Women's and Children's Health, Uppsala University, 751 85 Uppsala, Sweden

[2]Mood Disorders Centre, School of Psychology, University of Exeter, Perry Road, Devon, EX4 4QG, United Kingdom,

[3]Reproductive Health, Department of Women's and Children's Health, Uppsala University, 751 85 Uppsala, Sweden

Word Count

3,771

For peer review only

For peer review only - http://bmjopen.bmj.com/site/about/guidelines.xhtml

ABSTRACT

Introduction: Peripartum depression is a common mental health difficulty associated with a range of negative impacts for the mother, infant and wider family. Existing reviews suggest cognitive-behavioural therapy (CBT) based interventions are effective for peripartum depression, but there has been little examination of the evidence base concerning secondary outcomes (e.g. anxiety, stress and parenting), clinical and methodological moderators associated with effectiveness, and intervention acceptability. The review aims to examine the overall effectiveness of CBT based interventions for peripartum depression, alongside impact on secondary outcomes and moderators potentially associated with effectiveness. Further, this review aims to explore the acceptability of CBT based interventions for women with peripartum depression and examine important adaptations for this population.

Methods and analysis: Electronic databases; (e.g., MEDLINE; ISI Web of Science; CINAHL; CENTRAL; Prospero; EMBASE; ASSIA; PsycINFO; SCOPUS; and SweMed+) will be systematically searched. Database searches will be supplemented by expert contact, reference and citation checking, and grey literature. Primary outcomes of interest will be validated measures of peripartum depression. A proposed meta-analysis will examine: (1) the overall effectiveness of psychological interventions in relation to peripartum depression; (2) the impact of interventions on secondary outcomes (e.g. anxiety, stress and parenting); (3) clinical and methodological moderators associated with effectiveness. A thematic synthesis will be conducted on qualitative data exploring the acceptability of CBT intervention for women with postpartum depression including participants' experience and perspectives of the interventions, satisfaction, barriers and facilitators to intervention use, intervention relevance to mothers'

For peer review only

For peer review only - http://bmjopen.bmj.com/site/about/guidelines.xhtml

situations and suggestions for improvements as well as adaptations made to tailor CBT interventions to the perinatal client group.

Ethics and dissemination: Formal ethical approval is not required as primary data will not be collected. The results will be disseminated through a peer-reviewed publication and inform the development of a new psychological intervention for women with peripartum depression. This study including protocol development will run from March 2019 – March 2020.

STRENGTHS AND LIMITATION OF THIS STUDY

- Systematic review adopts quality standards informed by the Centre of Reviews and Dissemination guidance and PRISMA-P guidelines such as; independent study selection, data extraction and risk of bias assessments by two researchers.
- The review examines the effectiveness of CBT based peripartum depression interventions on secondary outcomes (e.g. anxiety, stress and parenting) and methodological moderators of effectiveness of CBT for peripartum depression.
- The first review of CBT interventions for peripartum depression to synthesise qualitative data regarding participants experience and perspectives of CBT based interventions for peripartum depression.
- High levels of clinical heterogeneity may exist as a consequence of included studies defining and delivering CBT based interventions in a variety of ways.
- Due to resource limitations, selected studies are only those publically available in the English or Swedish language; therefore, language bias may be present.

For peer review only - http://bmjopen.bmj.com/site/about/guidelines.xhtml

INTRODUCTION

Peripartum depression (PPD) is a common mental health difficulty associated with low mood during pregnancy (antepartum period) and after childbirth (postpartum period), with prevalence rates ranging between 10-23%.[1,2] Recent studies have demonstrated that depression may be more prevalent during the postpartum period than in age matched controls. [3] Further, approximately 40% of women will experience their first depressive episode postpartum,[4] and when left untreated, are more likely to experience further episodes of depression later in life.[5] The impact of PPD is significant for both mothers, infants and the wider family. For mothers PPD is associated with poor health-related quality of life and in extreme cases loss of life through suicide.[6] Further, postpartum depression is associated with increased risk for impairments in the mother's perceived bonding and attachment to the infant, both during the antepartum and postpartum period; in the postpartum period this is related to impairments in maternal responsiveness or sensitivity (hereafter referred to as parenting).[7] Prospectively, PPD is related to impairments in the infant's cognitive, social and emotional functioning.[8,9] Maternal postpartum depression is also associated with increased marital discord [10] and depression in fathers.[11] Given the prevalence and impact of PPD, it is important to identify and develop effective interventions.

Existing reviews highlight the effectiveness of a number of interventions for PPD including medication (e.g. antidepressant medication), health promotion interventions (e.g. educational home visits), and psychological interventions (e.g., cognitive behavioural therapy (CBT) &

For peer review only - http://bmjopen.bmj.com/site/about/guidelines.xhtml

interpersonal therapy (IPT).[12–23] However, the cost/benefit ratio for medication shifts for PPD given unclear evidence concerning safety during pregnancy [24], and mothers concerns regarding medication use whilst breastfeeding [25]. As such, women experiencing PPD indicate a preference for psychological interventions over medication. [25] Although there are mixed findings regarding the superiority of one type of psychological intervention over another,[13,15,23] several studies have found CBT to be an effective psychological intervention for PPD.[14,21,23] However, although evidence suggests CBT is an effective psychological intervention for PDD, the effect of CBT on secondary outcomes (e.g. anxiety, stress and parenting) has been largely unexamined. It is important to investigate the impact of PPD interventions on such secondary outcomes given they are commonly comorbid with PPD [26–28] and associated with poor infant developmental trajectories with a range of adverse outcomes including emotional problems, symptoms of attention deficit hyperactivity disorder, and impaired cognitive development.[29]

Further, the current evidence base for CBT lacks a systematic examination of moderators of the effects of CBT on PPD, for example, method of delivery (e.g., individual face-to-face, group or internet-administered) and health professional delivering intervention (e.g., clinical psychologist or midwife). Identifying key moderators may help identify factors associated with improved PPD outcomes. Additionally, further research is needed to examine barriers to accessing psychological interventions that are potentially related to intervention engagement and acceptability. Recent studies have found low rates of intervention engagement in PPD populations, with up to 86% of those suitable for psychological interventions failing to receive any support.[30] Previous qualitative studies have highlighted both practice (e.g. transportations

For peer review only

For peer review only - http://bmjopen.bmj.com/site/about/guidelines.xhtml

difficulties, attending appointments around feeding and sleep schedules, childcare needs) and psychological (stigma, sleep deprivation, fear of having child removed) barriers.[21,31] Although there is some evidence to suggest interventions that are adapted to address these barriers, can improve intervention engagement [17] there is a lack of systematic research investigating how such barriers are addressed and how uniquely tailoring interventions to the peripartum period may increase engagement and acceptability. As such, we will examine qualitative studies preceding eligible randomised controlled trails (RCTs) regarding intervention development and qualitative studies embedded within RCTs [32] that describe participants' experiences, perspectives and adaptations made to tailor CBT interventions to mothers situations associated with acceptability.

This review seeks to provide an updated systematic review of CBT based interventions for PPD. Meta-analysis, data permitting, will be used to examine the effect of CBT on the main outcome, depression and secondary outcomes (e.g. anxiety, stress and parenting). To address the aforementioned gaps in the literature the planned meta-analysis will also include potential moderators. Further, data from preceding or embedded qualitative studies [32] will be synthesized to examine women's experiences and perspectives of the interventions. Together this will provide a comprehensive review of the best current evidence regarding CBT based interventions for PPD that can be used to inform future intervention development.

Objectives: (1) to examine the effectiveness of CBT based interventions for PPD on symptoms of depression; (2) to examine the effectiveness of CBT based interventions for PPD on secondary outcome measures including; anxiety, stress (individual and perceived parenting stress),

For peer review only - http://bmjopen.bmj.com/site/about/guidelines.xhtml

parenting (e.g., sensitivity/responsiveness) perceived social support and perceived parental competence; (3) to investigate clinical and methodological moderators potentially associated with effectiveness; and (4) to describe the acceptability of CBT based interventions for PPD including (but not limited to) participants' experience and perspectives of the interventions, satisfaction, barriers and facilitators to intervention use, intervention relevance to mothers' situations and suggestions for improvements; and (5) to identify adaptations to tailor CBT based interventions to mothers situations potentially associated with acceptability.

METHODS AND ANALYSIS

Design: The protocol for this systematic review has been developed in accordance with the Preferred Reporting Items for Systematic Review and Meta-Analysis Protocols (PRISMA-P) 2015 statement [33] (see online supplementary appendix 1) and registered with PROSPERO International Prospective Register of Systematic Reviews (#CRDXXXXXXX). Protocol amendments will be added to PROSPERO. This systematic review will be conducted in accordance with the Cochrane Collaboration guidance for systematic reviews [34] and reported in accordance with the PRISMA statement. [35]

Patient and public involvement: Due to project funding constraints patients and members of the public were not involved in the design of this systematic review protocol. However, we will involve mothers with lived experience of PPD regarding the synthesis of the qualitative findings and considering implications of the results for future practice.

For peer review only - http://bmjopen.bmj.com/site/about/guidelines.xhtml

Inclusion and exclusion criteria: Study inclusion and exclusion criteria were developed using the following domains: Patient; Intervention; Comparison; Outcome; and Study Design (PICOS).[36]

Participants*.* Adult women (aged ≥16 years) from pregnancy to 12 months postpartum with either; (1) a diagnosis of PPD e.g., Diagnostic and Statistical Manual of Mental Disorders (DSM) IV or V [37,38]; or (2) reporting depression symptomatology within the peripartum period using a validated tool e.g., Edinburgh Postnatal Depression Scale (EPDS;[39]). Exclusion criteria are; (1) intervention for mood disorders other than depression (e.g. bipolar affective disorder); and (2) interventions focused on prevention of maternal psychopathology in at-risk, but not currently symptomatic mothers.[21]

Interventions. Only studies investigating interventions explicitly targeting PPD will be included. Eligible interventions will state the use of CBT, including behavioural activation or problem-solving, and contain specific therapeutic components associated with these approaches. CBT will be defined as interventions in which the focus is modifying a client's dysfunctional thoughts on current behaviour and future functioning.[13] BA will be defined as interventions targeting reductions in behavioural avoidance and increases in positively reinforcing activities, including interventions that focus on scheduling behaviours.[40] Problem-solving interventions will be defined as a psychological intervention including the following elements: definition of personal problems, generation of multiple solutions to each problem, selection of the best solution, developing a systematic plan for this solution, and evaluating whether the solution has resolved the problem.[13] There will be no exclusions regarding professional group supporting the

For peer review only - http://bmjopen.bmj.com/site/about/guidelines.xhtml

intervention, the clinical setting of the intervention, the method of delivery (individual face-to-face, group or internet-administered), or support methods used (e.g., e-mail, face-to-face, telephone). Additionally, self-guided / self-administered interventions will be eligible for inclusion.

Comparators**.** Control conditions eligible for inclusion will be based on standard definitions,[41] with eligible control conditions including: (1) no-treatment control; (2) wait-list control; (3) treatment-as-usual (TAU); (4) non-specific factors component control; (5) specific factors component control; and (6) active comparator. Only trial designs that allow for the isolation of the effects of CBT will be included given it is important for active comparators to discriminate intervention effects.[42] For example, a study comparing CBT alone versus medication alone would be excluded as it would not be possible to isolate the effect of the CBT.

Outcomes. This is a mixed methods systematic review involving both quantitative and qualitative outcomes. [43]

Quantitative outcomes. Studies eligible for inclusion will use a self-report or proxy/clinician administered standardised measurement of depression (e.g., the Beck Depression Inventory;[44] or PPD (e.g., EPDS).[39] In order to ensure the quality of the outcome measurements concerning the primary outcome of depression only studies using depression outcome measurements with at least acceptable internal consistency and test-retest reliability at Cronbach's alpha ≥0.70 (as reported in outcome measurement validation studies) will be included.[45] Secondary outcomes of interest are validated self-report measurements of (1) anxiety, (e.g., the Beck Anxiety

For peer review only

For peer review only - http://bmjopen.bmj.com/site/about/guidelines.xhtml

Inventory;[46] (2) individual stress (e.g., Perceived Stress Scale;[47]; (3) perceived parental stress (e.g., Parenting Stress Index;[48]; (4) self-report parenting (e.g., sensitivity/responsiveness such as; Postpartum Bonding Questionnaire;[49]; (5) perceived social support (e.g., Multidimensional Scale of Perceived Social Support;[50]; and (6) parental competence (e.g., Parenting Sense of Competence Scale).[51] Additionally observational parenting (e.g., sensitivity/responsiveness) measures (e.g., video tapes assessed with mind-mindedness coding manual;[52] will be included as secondary outcome measurements.

Qualitative outcomes. Studies eligible for inclusion will be; (1) qualitative studies preceding RCT studies to develop the intervention(s) examined in RCTs or qualitative studies embedded within RCT studies; and (2) examining acceptability of interventions (e.g., participants' experience and perspectives of the interventions, satisfaction, barriers and facilitators to intervention use, intervention relevance to mothers' situations and suggestions for improvements); and/or (3) examining adaptations made to tailor the intervention to the population. If mixed methods studies are identified using quantitative measures of intervention acceptability (e.g., the Treatment Acceptability/Adherence Scale TAAS; [53]) this data will be extracted and triangulated with the qualitative findings regarding acceptability.

Study Designs: Both studies with quantitative and qualitative designs will be included. *Quantitative:* To examine effectiveness only RCTs will be included, with non-randomised and uncontrolled designs excluded. RCT designs were chosen for this systematic review as they as they are able to eliminate many of the biases inherent with other study designs.[54] Further, RCTs with randomisation procedures at allocation and concealment rated as high risk of bias, in

For peer review only - http://bmjopen.bmj.com/site/about/guidelines.xhtml

line with the revised Cochrane risk-of-bias tool for randomized trials (RoB 2), [55] will be excluded (see online supplementary appendix 2).

Qualitative: Data from preceding or embedded qualitative studies within included RCTs will be examined for mothers' experiences and perspectives of interventions and potential adaptations of interventions.

Search methods:

Electronic searches. Searches will be carried out in relevant international electronic databases; MEDLINE; ISI Web of Science; Cumulative Index to Nursing and Allied Health Literature (CINAHL); Cochrane Central Register of Controlled Trials (CENTRAL); Prospero; Excerpta Medica DataBase (EMBASE); Applied Social Sciences Index and Abstracts (ASSIA); PsycINFO; SCOPUS; and SweMed+. Searches will also be conducted on clinical trial registers (www.ClinicalTrials.gov and www.who.int/trialsearch/) and conference proceedings (BIOSIS Previews; Index to Scientific and Technical Proceedings, Health Management Consortium and Web of Science with Conference Proceedings). Databases will be searched using medical subject headings (MeSH) and text words in the title and abstract (see online supplementary appendix 3). The search strategy was developed alongside Agnes Kotka (librarian at Uppsala University Library) and reviewed by Professor Alkistis Skalkidou (Uppsala University) using the PRESS Peer Review Guidelines; [56]; see online supplementary appendix 4). No date limits will be imposed. Studies published in English and Swedish will be accepted.

Other resources. Forward citation searches will be conducted for all included studies and reference lists of all of the included studies will be hand searched. Studies included within identified secondary evidence reports (e.g. relevant systematic reviews and meta-analyses) will

For peer review only - http://bmjopen.bmj.com/site/about/guidelines.xhtml

also be reviewed. Systematic reviews will only be included in the hand search if they meet the following criteria; (1) search of at least one database; (2) reporting of selection criteria; (3) a quality appraisal of included studies; and (4) provide a list and synthesis of included studies.[57]

Additionally, the five journals containing the highest numbers of included studies will be hand searched for recent potentially eligible publications in the past 12 months.[58] Grey literature will be searched using; OpenGrey, a system for grey literature produced in Europe, such as research reports, doctoral dissertations and conference papers (http://www.opengrey.eu/); ProQuest a dissertation and thesis database (http://www.proquest.com/en-US/catalogs/databases/detail/ pqdt.shtml); and DIVA a portal for searching publications from universities managed by Uppsala University. Searches for relevant dissertations will be conducted; however, due to time and funding limitations full dissertations will not be reviewed. The references for potentially eligible dissertations will be made available as part of a list of excluded studies. Potentially relevant studies published in a languages other than English and Swedish will not be included within the review due to lack of resources for translation, and will be provided as part of a list of excluded studies.

Screening and data extraction:

Screening. Duplicates of studies across searches will be identified and removed. Disagreements regarding inclusion will be discussed between the two reviewers, with a third reviewer consulted if consensus cannot be reached. Two independent reviewers will conduct a broad screen of study titles and abstracts, followed by full paper checks of potentially eligible studies. Studies will be excluded that do not clearly meet the outlined PICOS criteria (see online supplementary appendix 2). In the case of studies in which missing data is needed to determine eligibility,

For peer review only

For peer review only - http://bmjopen.bmj.com/site/about/guidelines.xhtml

authors will be contacted by email for additional information. For authors that do not respond to the first email within two weeks a follow up email with be sent.

Data extraction.

Quantitative extraction. Two reviewers will independently extract data from the included studies following Centre for Reviews and Dissemination (CRD) guidance. [59] Data will be extracted according to data extraction form (see online supplementary appendix 5) including; (1) study characteristics; (2) participant characteristics; (3) intervention characteristics; (4) study outcome measurements; and (5) quantitative results

Qualitative extraction. Qualitative studies (preceding or embedded within included RCT studies) focused on CBT for PPD intervention acceptability and intervention adaptations will be sought by two reviewers. Data from these studies will be imported into NVivo [60] software for analysis.

Discrepancies will be discussed between the two reviewers, with a third reviewer consulted if consensus cannot be reached. Study authors will be contacted in the event of missing data.

Risk of bias assessment. Methodological quality of included studies will be assessed using the Cochrane Collaboration's Risk of Bias tool 2.0.[55] Two reviewers will work independently to determine the adequacy of (1) randomisation; (2) allocation to intervention; (3) adherence to intervention; (4) handling of missing outcome data; (5) measurement of outcome; and (6) selection of the reported results. Ratings will be compared and any discrepancies discussed, and

For peer review only - http://bmjopen.bmj.com/site/about/guidelines.xhtml

if consensus is not reached, further discussion will be held with a third reviewer. To assess reporting bias study protocols will be sought for all included studies either via published protocols, trial databases or emailing study authors. Comparisons will be made between outcome measurements reported in the protocol and the paper as well as outcomes reported in the methods and results section of published trial results. Study authors will be contacted to clarify discrepancies and to identify potential changes to the study protocol and request any missing data. Consistent with a previous meta-analysis,[61] in order to include quality as a moderator each area addressing risk of bias will be given a score of 0, 1 or 2, indicating bias based on the Risk of Bias tool 2.0,[55] yielding a possible total score of 12. The ratings will be defined as high (9-12), unclear (4-8), or low risk (0-3) respectively. All findings will be summarised within a table to allow easy comparison across studies.

Data synthesis and statistical analysis: Quantitative and qualitative data will be analysed separately.[62] Quantitative data will be managed using an extraction database developed in Microsoft Access for this review and analysed in Comprehensive Meta-analysis (version 2). Qualitative data will be managed and analysed using NVivo10.[60]

Measures of intervention effect. If data allows, a meta-analysis will be conducted. Hedges' g will be calculated to determine the post-treatment between-group standardised mean effect size from outcomes relating to the primary (depression) and secondary outcomes (anxiety, individual stress, perceived parental stress, parenting, perceived social support and parental competence). Where multiple time points are reported, a primary end point ≤6 months post-treatment will be adopted.[63] However, outcomes for all time points reported will be examined to enable a potential moderator analysis on length of follow-up. The control condition sample size will be

For peer review only - http://bmjopen.bmj.com/site/about/guidelines.xhtml

halved for studies where two interventions eligible for inclusion are compared with one control condition and comparisons will be analysed separately. Additionally, in studies comparing two control conditions with one intervention condition, comparisons will be analysed separately with the sample size in the intervention condition halved [64].

Dealing with missing data. Study authors will be contacted to provide missing means and standard deviations of post-treatment measurement scores. Intention-to-treat principles will be followed as far as possible.

Assessment of Heterogeneity. Heterogeneity is anticipated due to variations in interventions the studies, participant characteristics, and methodological factors, therefore, a random effects model [65] will be adopted with Q and I^2 [66] reported alongside confidence intervals as measures of heterogeneity. If this assumption is not met in the Q and I^2 analysis, a fixed effects model will be used.

Sensitivity analysis. A sensitivity analysis regarding the overall effect size of the primary outcome measurement (depression) will be conducted by temporary removal of (1) each study individually from the overall analysis and the effect size recalculated; (2) small studies ($n \leq 20$ across conditions); and/or (3) studies with high attrition rates (≥30% in at least one arm).[58]

Funnel asymmetry. If there is a minimum of 10 studies in any meta-analysis, funnel plot asymmetry will be examined for sources of possible bias (e.g., publication bias, language bias, inclusion of small studies with poor methodological quality, and heterogeneity).[67,68] An

For peer review only - http://bmjopen.bmj.com/site/about/guidelines.xhtml

estimated effect size taking these biases into account will be calculated using the trim and fill procedure.[69] These will be analysed separately for each outcome.

Moderator analysis. If sufficient data are available moderator analysis will be undertaken to examine clinical and methodological components and participant characteristics of studies potentially associated with effectiveness. The following moderators will be examined:

- Risk of bias (e.g., high, unclear or low)
- Type of control condition used (e.g., no-treatment control, wait-list control, TAU, non-specific factors component control, specific factors component control and active comparator).[70]
- Length of follow up (e.g., short; post-treament-3 months, medium; 3-6 months, long; 7-11 months; extended; 12 months+)
- Severity of depression at baseline (e.g., severe, moderate or mild)
- Type of CBT intervention (e.g., CBT, BA or problem solving)
- Interventions including additional social components (e.g., peer support group or involvement of partner). Social components will be defined as interventions facilitating contact with peers or partners in a way that encourages nondirective (without specific psychological techniques) support, such as helping people to express their experiences and emotions and offering empathy.
- Interventions including parenting intervention components (e.g., video interaction guidance).[71] Parenting intervention components will be defined as interventions that

For peer review only

include specific support in relation to the parent infant relationship this could be via specific sessions with a therapist, video feedback or self-help materials.

- Method of delivery (e.g., face to face, group or internet-administered)
- Point of intervention (e.g., antepartum or postpartum period)
- Health professional delivering intervention (e.g., clinical psychologist or midwife)

With heterogeneity being anticipated, random effects will be adopted with Q and I^2 reported as measures of heterogeneity.

Qualitative data synthesis. Thematic synthesis will be used to analyse qualitative data.[72] In this review eligible qualitative data will be defined as all text labelled as 'results' or 'findings' in study reports.[72] Similar to analysis of primary qualitative datasets, thematic synthesis involves the systematic coding of data and generating of descriptive and analytical themes. This data will be analysed following a three step process. First, study data will be coded line by line by the first reviewer and checked by a second reviewer with a coding frame developed from codes derived from the data. Second, similar codes will be grouped together to form new ‘descriptive themes’. Disparities or discrepancies in coding and theme development will be resolved through discussion or in consultation with a third reviewer if necessary; with the coding frame adjusted accordingly. Once coding is complete the two reviewers will consider each theme and sub-theme and examine the text within them for consistency, code interrelations, and identify any potential conceptual hierarchies. Third, analytic themes will be developed to ‘go beyond’ the primary reported data by synthesising findings across studies and interpreting their meaning in relation to review objectives. Narrative descriptions of each theme will be provided, alongside supporting

For peer review only - http://bmjopen.bmj.com/site/about/guidelines.xhtml

quotations. All changes to codes, conceptual re-alignments, discussions and decisions will be documented as part of the review audit trail.

SUMMARY

This review represents the first review of interventions for PPD that includes mixed methods to examine the overall effectiveness of CBT (including its impact of secondary outcomes and potential moderators of the effect). The review also examines the acceptability of CBT interventions for PPD and adaptations to potentially improve acceptability of CBT for a PPD population. Conclusions will be drawn in the context of the limitations of the methodology adopted for this review. However the review will provide a necessary step in addressing the current state of the CBT evidence base for PPD and results will have the potential to inform future PPD intervention developments in line with phase I of the Medical Research Council framework for developing complex interventions. [73]

DISSEMINATION PLAN

Dissemination to relevant audiences is a necessary step towards impact. We will report review results through scientific conferences and publications, as well as publications for professional and lay audiences and meetings. We will also provide summaries of results and offer to present these for key parties including Health Insurance Directors and National and County Level Health Policy representatives. The authors will use social media and attend participatory involvement

For peer review only - http://bmjopen.bmj.com/site/about/guidelines.xhtml

events to promote the review results and patient representatives will be contacted to feature results on social media, blogs and vlogs.

REFERENCES

1 Howard LM, Molyneaux E, Dennis C-LL, *et al.* Non-psychotic mental disorders in the perinatal period. *Lancet* 2014;**384**:1775–88. doi:10.1016/S0140-6736(14)61276-9

2 Paulson JF, Bazemore SD, Prevalence HE, *et al.* Prenatal and postpartum depression in fathers. *Methods* 2010;**303**:1961–9. doi:10.1001/jama.2010.605.

3 Merkitch KG, Jonas KG, O'Hara MW. Modeling trait depression amplifies the effect of childbearing on postpartum depression. *J Affect Disord* 2017;**223**:69–75. doi:10.1016/j.jad.2017.07.017

4 Woolhouse H, Gartland D, Mensah F, *et al.* Maternal depression from early pregnancy to 4 years postpartum in a prospective pregnancy cohort study: Implications for primary health care. *BJOG An Int J Obstet Gynaecol* 2015;**122**:312–21. doi:10.1111/1471-0528.12837

5 Josefsson A, Sydsjö G. A follow-up study of postpartum depressed women: Recurrent maternal depressive symptoms and child behavior after four years. *Arch Womens Ment Health* 2007;**10**:141–5. doi:10.1007/s00737-007-0185-9

6 Bauer A, Knapp M, Parsonage M. Lifetime costs of perinatal anxiety and depression. *J Affect Disord* 2016;**192**:83–90. doi:10.1016/j.jad.2015.12.005

7 Stein A, Netsi E, Lawrence PJ, *et al.* Mitigating the effect of persistent postnatal depression on child outcomes through an intervention to treat depression and improve parenting: a randomised controlled trial. *Lancet Psychiatry* 2018;**5**:134–44. doi:10.1016/s2215-0366(18)30006-3

8 Stein A, Netsi E, Lawrence PJ, *et al.* Mitigating the effect of persistent postnatal depression on child outcomes through an intervention to treat depression and improve parenting: a randomised controlled trial. *The Lancet Psychiatry* 2018;**5**:134–44. doi:10.1016/S2215-0366(18)30006-3

9 Netsi E, Pearson RM, Murray L, *et al.* Association of persistent and severe postnatal depression with child outcomes. *JAMA Psychiatry* 2018;**75**:247–53. doi:10.1001/jamapsychiatry.2017.4363

10 Letourneau NL, Dennis CL, Benzies K, *et al.* Postpartum depression is a family affair: Addressing the impact on mothers, fathers, and children. *Issues Ment Health Nurs* 2012;**33**:445–57. doi:10.3109/01612840.2012.673054

11 Massoudi P, Hwang CP, Wickberg B. Fathers' depressive symptoms in the postnatal period: Prevalence and correlates in a population-based Swedish study. *Scand J Public Health* 2016;**44**:688–94. doi:10.1177/1403494816661652

12 Clarke K, King M, Prost A. Psychosocial interventions for perinatal common mental disorders delivered by providers who are not mental health specialists in low- and middle-income countries: A systematic review and meta-analysis. *PLoS Med* 2013;**10**:e1001541. doi:10.1371/journal.pmed.1001541

13 Cuijpers P, van Straten A, Andersson G, *et al.* Psychotherapy for depression in adults: A

For peer review only - http://bmjopen.bmj.com/site/about/guidelines.xhtml

meta-analysis of comparative outcome studies. *J Consult Clin Psychol* 2008;**76**:909–22. doi:10.1037/a0013075
14 Stamou G, García-palacios A, Botella C. Cognitive-Behavioural therapy and interpersonal psychotherapy for the treatment of post-natal depression : a narrative review. *BMC Psychol* 2018;**18**. doi:10.1186/s40359-018-0240-5
15 Stephens S, Ford E, Paudyal P, *et al.* Effectiveness of Psychological Interventions for Postnatal Depression in Primary Care: A Meta-Analysis. *Ann Fam Med* 2016;**14**:463–72. doi:10.1370/afm.1967
16 Dennis CL. Treatment of postpartum depression, part 2: a critical review of nonbiological interventions. *J Clin Psychiatry* 2004;**65**:1252–65. doi:10.4088/JCP.v65n0915
17 Dennis C-L, Dowswell T. Psychosocial and psychological interventions for preventing postpartum depression. *Cochrane Database Syst Rev* Published Online First: 28 February 2013. doi:10.1002/14651858.CD001134.pub3
18 Dennis C-L, Hodnett ED. Psychosocial and psychological interventions for treating postpartum depression. *Cochrane Database Syst Rev* Published Online First: 17 October 2007. doi:10.1002/14651858.CD006116.pub2
19 Leis JA, Mendelson T, Tandon SD, *et al.* A systematic review of home-based interventions to prevent and treat postpartum depression. *Arch Womens Ment Health* 2009;**12**:3–13. doi:http://0-dx.doi.org.lib.exeter.ac.uk/10.1007/s00737-008-0039-0
20 Morrell CJ, Sutcliffe P, Booth A, *et al.* A systematic review, evidence synthesis and meta-analysis of quantitative and qualitative studies evaluating the clinical effectiveness, the cost-effectiveness, safety and acceptability of interventions to prevent postnatal depression. *Health Technol Assess (Rockv)* 2016;**20**:1–414. doi:10.3310/hta20370
21 Nillni YI, Mehralizade A, Mayer L, *et al.* Treatment of depression, anxiety, and trauma-related disorders during the perinatal period: A systematic review. *Clin Psychol Rev* Published Online First: 2018. doi:10.1016/j.cpr.2018.06.004
22 Scope A, Leaviss J, Kaltenthaler E, *et al.* Is group cognitive behaviour therapy for postnatal depression evidence-based practice? A systematic review. *BMC Psychiatry* 2013;**13**:321. doi:10.1186/1471-244X-13-321
23 Sockol LE, Epperson CN, Barber JP. A meta-analysis of treatments for perinatal depression. *Clin Psychol Rev* 2011;**31**:839–49. doi:10.1016/j.cpr.2011.03.009
24 Tak CR, Job KM, Schoen-Gentry K, *et al.* The impact of exposure to antidepressant medications during pregnancy on neonatal outcomes: a review of retrospective database cohort studies. *Eur J Clin Pharmacol* 2017;**73**:1055–69. doi:10.1007/s00228-017-2269-4
25 Battle CL, Salisbury AL, Schofield CA, *et al.* Perinatal antidepressant use: understanding women's preferences and concerns. *J Psychiatr Pract* 2013;**19**:443–53. doi:10.1097/01.pra.0000438183.74359.46
26 Putnam KT, Wilcox M, Robertson-Blackmore E, *et al.* Clinical phenotypes of perinatal depression and time of symptom onset: analysis of data from an international consortium. *The Lancet Psychiatry* 2017;**4**:477–85. doi:10.1016/S2215-0366(17)30136-0
27 Biaggi A, Conroy S, Pawlby S, *et al.* Identifying the women at risk of antenatal anxiety and depression: A systematic review. J. Affect. Disord. 2016;**191**:62–77. doi:10.1016/j.jad.2015.11.014
28 Pechtel P, Murray LMM, Brumariu LE, *et al.* Reactivity, regulation, and reward responses to infant cues among mothers with and without psychopathology: an fMRI review. *Transl Dev Psychiatry* 2013;**1**:19673. doi:10.3402/tdp.v1i0.19673

For peer review only - http://bmjopen.bmj.com/site/about/guidelines.xhtml

29 Sanger C, Iles JE, Andrew CS, *et al.* Associations between postnatal maternal depression and psychological outcomes in adolescent offspring: a systematic review. *Arch Womens Ment Health* 2015;**18**:147–62. doi:10.1007/s00737-014-0463-2
30 Cox EQ, Sowa NA, Meltzer-Brody SE, *et al.* The perinatal depression treatment cascade. *J Clin Psychiatry* 2016;**77**:1189–200. doi:10.4088/JCP.15r10174
31 O'Mahen H, Fedock G, Henshaw E, *et al.* Modifying CBT for perinatal depression: what do women want?: a qualitative study. *Cogn Behav Pract* 2012;**19**:359–71. doi:10.1016/J.CBPRA.2011.05.005
32 Davis K, Minckas N, Bond V, *et al.* Beyond interviews and focus groups : a framework for integrating innovative qualitative methods into randomised controlled trials of complex public health interventions. 2019;**20**:1–16.
33 Moher D, Shamseer L, Clarke M, *et al.* Preferred reporting items for systematic review and meta-analysis protocols (PRISMA-P). *Syst Rev* 2015;**4**.
34 Higgins J., Green S. Cochrane handbook for systematic reviews of interventions version 5.1.0. *Cochrane Collab* 2011.
35 Moher D, Liberati A, Tetzlaff J, *et al.* Preferred reporting items for systematic reviews and meta-analyses: the PRISMA statement. *PLoS Med* 2009;**6**:e1000097. doi:10.1371/journal.pmed.1000097
36 Costantino G, Montano N, Casazza G. When should we change our clinical practice based on the results of a clinical study? Searching for evidence: PICOS and PubMed. *Intern Emerg Med* 2015;**10**:525–7. doi:10.1007/s11739-015-1225-5
37 American Psychiatric Association. *Diagnostic and statistical manual of mental disorders 4th edition : DSM-IV*. 1994.
38 American Psychiatric Association. *Diagnostic and statistical manual of mental disorders 5th edition: DSM-V*. Washington DC: American Psychaitric Association 2013.
39 Cox JL, Holden JM, Sagovsky R. Detection of postnatal depression. Development of the 10-item Edinburgh Postnatal Depression Scale. *Br J Psychiatry J Ment Sci* 1987;**150**:782–6.
40 Hopko DR, Lejuez CW, Ruggiero KJ, *et al.* Contemporary behavioral activation treatments for depression: procedures, principles, and progress. *Clin Psychol Rev* 2003;**23**:699–717. doi:10.1016/S0272-7358(03)00070-9
41 Mohr DC, Spring B, Freedland KE, *et al.* The selection and design of control conditions for randomized controlled trials of psychological interventions. *Psychother Psychosom* 2009;**78**:275–84. doi:10.1159/000228248
42 Evans SR. Fundamentals of clinical trial design. *J Exp Stroke Transl Med* 2010;**3**:19–27.
43 Pluye P, Nha Hong Q. Combining the power of stories and the power of numbers: mixed methods research and mixed studies reviews. *Annu Rev Public Health* 2014;**35**:29–45. doi:10.1146/annurev-publhealth-032013-182440
44 Beck AT, Steer RA, Brown GK. Manual for the Beck Depression Inventory. *San Antonio, TX Psychol Corp* 1996.
45 Fitzpatrick R, Davey C, Buxton MJ, *et al.* Evaluating patient-based outcome measures for use in clinical trials HTA Health Technology Assessment NHS R&D HTA Programme. *Health Technol Assess (Rockv)* 1998;**2**.
46 Beck AT, Epstein N, Brown G, *et al.* An inventory for measuring clinical anxiety: psychometric properties. *J Consult Clin Psychol* 1988;**56**:893–7.
47 Cohen S, Kamarck T, Mermelstein R. A global measure of perceived stress. *J Health Soc*

For peer review only

For peer review only - http://bmjopen.bmj.com/site/about/guidelines.xhtml

Behav 1983;**24**:385–96.
48 Abidin RR. Parenting stress index. *Psychol Assess Resour* 2012.
49 Brockington IF, Fraser C, Wilson D. The Postpartum Bonding Questionnaire: a validation. *Arch Womens Ment Health* 2006;**9**:233–42. doi:10.1007/s00737-006-0132-1
50 Zimet GD, Dahlem NW, Zimet SG, *et al.* The multidimensional scale of perceived social support. *J Pers Assess* 1988;**52**:30–41. doi:10.1207/s15327752jpa5201_2
51 Rogers H, Matthews J. The parenting sense of competence scale: Investigation of the factor structure, reliability, and validity for an Australian sample. *Aust Psychol* 2004;**39**:88–96. doi:10.1080/00050060410001660380
52 Meins E, Fernyhough C. Mind-mindedness coding manual, Version 2.0 . 2015;:1–22.
53 Milosevic I, Levy HC, Alcolado GM, *et al.* The treatment acceptability/adherence scale: moving beyond the assessment of treatment effectiveness. *Cogn Behav Ther* 2015;**44**:456–69. doi:10.1080/16506073.2015.1053407
54 Hariton E, Locascio JJ. Randomised controlled trials - the gold standard for effectiveness research: Study design: randomised controlled trials. *BJOG* 2018;**125**:1716. doi:10.1111/1471-0528.15199
55 Higgins JP, Savović J, Page MJ, *et al.* Revised Cochrane risk-of-bias tool for randomized trials (RoB 2). 9 October 2018. *Cochrane Database Syst Rev* Published Online First: 2018. doi:10.1002/14651858.CD201601
56 Mcgowan J, Sampson M, Salzwedel DM, *et al.* PRESS peer review explanation and strategies: 2015 guideline of electronic search elaboration. 2016.
57 Robinson KA, Whitlock EP, Oneil ME, *et al.* Integration of existing systematic reviews into new reviews: identification of guidance needs. *Syst Rev* 2014;**3**:60. doi:10.1186/2046-4053-3-60
58 Farrand P, Matthews J, Dickens C, *et al.* Psychological interventions to improve psychological well-being in people with dementia or mild cognitive impairment: systematic review and meta-analysis protocol. *BMJ Open* 2016;**6**:e009713. doi:10.1136/bmjopen-2015-009713
59 Centre for Reviews and Dissemination (CRD). Systematic reviews: CRD's guidance for undertaking reviews in health care. 2009. doi:10.1016/S1473-3099(10)70065-7
60 International Q. NVvio 10 for Windows. 2014.
61 Farrand P, Woodford J. Effectiveness of cognitive behavioural self-help for the treatment of depression and anxiety in people with long-term physical health conditions: a systematic review and meta-analysis of randomised controlled trials. *Ann Behav Med A Publ Soc Behav Med* 2015;**49**:579–93. doi:10.1007/s12160-015-9689-0
62 Tricco AC, Antony J, Soobiah C, *et al.* Knowledge synthesis methods for integrating qualitative and quantitative data: a scoping review reveals poor operationalization of the methodological steps. *J Clin Epidemiol* 2016;**73**:29–35. doi:10.1016/j.jclinepi.2015.12.011
63 Møller AM, Myles PS. What makes a good systematic review and meta-analysis? *Br J Anaesth* 2016;**117**:428–30. doi:10.1093/bja/aew264
64 Simblett S, Birch J, Matcham F, *et al.* A systematic review and meta-analysis of e-mental health interventions to treat symptoms of posttraumatic stress. *JMIR Ment Heal* 2017;**4**:e14. doi:10.2196/mental.5558
65 Higgins JPT, Thompson SG. Quantifying heterogeneity in a meta-analysis. *Stat Med* 2002;**21**:1539–58. doi:10.1002/sim.1186

For peer review only - http://bmjopen.bmj.com/site/about/guidelines.xhtml

66 Higgins JPT, Thompson SG, Deeks JJ, *et al.* Measuring inconsistency in meta-analyses. *BMJ Br Med J* 2003;**327**:557–60. doi:10.1136/bmj.327.7414.557
67 Egger M, Davey Smith G, Schneider M, *et al.* Bias in meta-analysis detected by a simple, graphical test. *BMJ* 1997;**315**:629–34. doi:10.1136/BMJ.315.7109.629
68 Sterne JA, Gavaghan D, Egger M. Publication and related bias in meta-analysis: power of statistical tests and prevalence in the literature. *J Clin Epidemiol* 2000;**53**:1119–29.
69 Duval S, Tweedie R. Trim and Fill: A Simple Funnel-Plot-Based Method. 2000;:455–63.
70 Mohr DC, Ho J, Hart TL, *et al.* Control condition design and implementation features in controlled trials: a meta-analysis of trials evaluating psychotherapy for depression. *Transl Behav Med* 2014;**4**:407–23. doi:10.1007/s13142-014-0262-3
71 Tooten A, Hoffenkamp HN, Hall RAS, *et al.* The effectiveness of video interaction guidance in parents of premature infants: A multicenter randomised controlled trial. *BMC Pediatr* 2012;**12**:1–9. doi:10.1186/1471-2431-12-76
72 Thomas J, Harden A. Methods for the thematic synthesis of qualitative research in systematic reviews. *BMC Med Res Methodol* 2008;**8**:45. doi:10.1186/1471-2288-8-45
73 Craig P, Dieppe P, Macintyre S, *et al.* Developing and evaluating complex interventions: The new Medical Research Council guidance. *Bmj* 2008;**337**:979–83. doi:10.1136/bmj.a1655

LIST OF ABBREVIATIONS

BA: Behavioural Activation
CBT: Cognitive Behavioural Therapy
CRD: Centre for Reviews and Dissemination
EPDS: Edinburgh Postnatal Depression Scale
DSM: Diagnostic and Statistical Manual of Mental Disorders
IPT: Interpersonal Therapy
MeSH: Medical Subject Headings
MRC: Medical Research Council
PDSS: Postpartum Depression Screening Scale
PPD: Peripartum Depression
RCT: Randomised controlled trial
TAU: Treatment as Usual

AUTHORS' CONTRIBUTIONS

DP, JW and HO conceptualised the study. DP drafted the protocol. DP, JW, HO, and ASS designed the study. DP, JW, and HO assisted with manuscript writing. LvE, CA and OB participated critical revision of the study design and manuscript. All authors read and approved the final manuscript.

For peer review only - http://bmjopen.bmj.com/site/about/guidelines.xhtml

ACKNOWLEDGEMENTS

Professor Alkistis Skalkidou from the Department of Women's and Children's Health at Uppsala University for providing peer review of the search strategy.

Agnes Kotka, Librarian at Uppsala University Library for assisting with the development of the electronic search strategy.

FUNDING STATEMENT

This work was supported by U-CARE, which is a strategic research environment funded by the Swedish Research Council (dnr 2009–1093). This funding source had no role in the design of this study and will not have any role during its execution, analyses, interpretation of the data or decision to submit results.

COMPETING INTERESTS STATEMENT

The authors declare that they have no competing interests.

For peer review only

For peer review only - http://bmjopen.bmj.com/site/about/guidelines.xhtml

PRISMA-P (Preferred Reporting Items for Systematic review and Meta-Analysis Protocols) 2015 checklist: recommended items to address in a systematic review protocol*

Section and topic	Item No	Checklist item	(Page No.#)
**ADMINISTRATIVE INFORMATION**			
Title:			
Identification	1a	Identify the report as a protocol of a systematic review	1
Update	1b	If the protocol is for an update of a previous systematic review, identify as such	n/a
Registration	2	If registered, provide the name of the registry (such as PROSPERO) and registration number	5 (following peer review)
Authors:			
Contact	3a	Provide name, institutional affiliation, e-mail address of all protocol authors; provide physical mailing address of corresponding author	1
Contributions	3b	Describe contributions of protocol authors and identify the guarantor of the review	15
Amendments	4	If the protocol represents an amendment of a previously completed or published protocol, identify as such and list changes; otherwise, state plan for documenting important protocol amendments	5
Support:			
Sources	5a	Indicate sources of financial or other support for the review	15-16
Sponsor	5b	Provide name for the review funder and/or sponsor	
Role of sponsor or funder	5c	Describe roles of funder(s), sponsor(s), and/or institution(s), if any, in developing the protocol	
**INTRODUCTION**			
Rationale	6	Describe the rationale for the review in the context of what is already known	3-4
Objectives	7	Provide an explicit statement of the question(s) the review will address with reference to participants, interventions, comparators, and outcomes (PICO)	4-6
**METHODS**			
Eligibility criteria	8	Specify the study characteristics (such as PICO, study design, setting, time frame) and report characteristics (such as years considered, language, publication status) to be used as criteria for eligibility for the review	5-6
Information sources	9	Describe all intended information sources (such as electronic databases, contact with study authors, trial registers or other grey literature sources) with planned dates of coverage	7
Search strategy	10	Present draft of search strategy to be used for at least one electronic database, including planned limits, such that it could be	7 and online

For peer review only

		repeated	supplementary appendix 3).
Study records:			
Data management	11a	Describe the mechanism(s) that will be used to manage records and data throughout the review	8
Selection process	11b	State the process that will be used for selecting studies (such as two independent reviewers) through each phase of the review (that is, screening, eligibility and inclusion in meta-analysis)	7-8
Data collection process	11c	Describe planned method of extracting data from reports (such as piloting forms, done independently, in duplicate), any processes for obtaining and confirming data from investigators	7-8 and online supplementary appendix 5
Data items	12	List and define all variables for which data will be sought (such as PICO items, funding sources), any pre-planned data assumptions and simplifications	online supplementary appendix 5
Outcomes and prioritization	13	List and define all outcomes for which data will be sought, including prioritization of main and additional outcomes, with rationale	6
Risk of bias in individual studies	14	Describe anticipated methods for assessing risk of bias of individual studies, including whether this will be done at the outcome or study level, or both; state how this information will be used in data synthesis	6, 9 and online supplementary appendix 2
Data synthesis	15a	Describe criteria under which study data will be quantitatively synthesised	9 and 10
	15b	If data are appropriate for quantitative synthesis, describe planned summary measures, methods of handling data and methods of combining data from studies, including any planned exploration of consistency (such as I^2, Kendall's τ)	9
	15c	Describe any proposed additional analyses (such as sensitivity or subgroup analyses, meta-regression)	9 and 10
	15d	If quantitative synthesis is not appropriate, describe the type of summary planned	n/a
Meta-bias(es)	16	Specify any planned assessment of meta-bias(es) (such as publication bias across studies, selective reporting within studies)	9
Confidence in cumulative evidence	17	Describe how the strength of the body of evidence will be assessed (such as GRADE)	8 and 9

*** It is strongly recommended that this checklist be read in conjunction with the PRISMA-P Explanation and Elaboration (cite when available) for important clarification on the items. Amendments to a review protocol should be tracked and dated. The copyright for PRISMA-P (including checklist) is held by the PRISMA-P Group and is distributed under a Creative Commons Attribution Licence 4.0.**

From: Shamseer L, Moher D, Clarke M, Ghersi D, Liberati A, Petticrew M, Shekelle P, Stewart L, PRISMA-P Group. Preferred reporting items for systematic review and meta-analysis protocols (PRISMA-P) 2015: elaboration and explanation. BMJ. 2015 Jan 2;349(jan02 1):g7647.

For peer review only

PICOS STATEMENT

Table 1: PICOS statement

PICOS	Eligibility Criteria	√
Population	Adult women (aged ≥ 16 years)	❑
	Antepartum or postpartum (up to 12 months)	❑
	Depressive disorder with peripartum onset<br><br>For example:<br>• A diagnosis of major depression with peripartum onset specifier in accordance with the Diagnostic and Statistical Manual of Mental Disorders (DSM) IV or V.[37,38]<br>• Reporting depression symptomatology with a peripartum onset using a validated tool e.g., Edinburgh Postnatal Depression Scale (EPDS).[39]	❑
	Exclusion criteria: intervention for mood disorders other than depression (e.g. bipolar affective disorder).	❑ Does not contain
	Exclusion criteria: interventions focussed on prevention of maternal psychopathology in at-risk, but not currently symptomatic mothers.	❑ Does not contain
Intervention	Interventions explicitly targeting an improvement in peripartum depression e.g., not targeting mother-infant interaction.<br><br>*Note:* no exclusions are placed upon:<br>• Professional group supporting the intervention<br>• Clinical setting of the intervention<br>• Delivery mode (self-guided, individual or group)<br>• Support methods (internet, face-to-face or telephone)	❑
	Intervention explicitly states the use of cognitive behavioural therapy (CBT), behavioural activation (BA) and/or problem-solving.<br><br>For example:<br>• CBT will be defined as interventions in which the focus is modifying a client's dysfunctional thoughts on current behaviour and future functioning.[13]<br>• BA will be defined as interventions targeting reductions in behavioural avoidance and increases in positively reinforcing activities, including interventions that focus on scheduling behaviours.[40]<br>• Problem-solving interventions will be defined as a psychological intervention including the following elements: definition of personal problems, generation of multiple solutions to each problem, selection of the best solution, developing	❑

For peer review only

	a systematic plan for this solution, and evaluating whether the solution has resolved the problem. [13]	
Comparison	Trial contains a suitable control condition<br><br>For example:<br><br>• no-treatment control<br>• wait-list control<br>• treatment-as-usual<br>• non-specific factors component control<br>• specific factors component control<br>• active comparator<br><br>*Note:* only designs that isolate the effects of CBT should be included. For example, a study comparing CBT alone versus medication alone would be excluded as it would not be possible to isolate the effect of the CBT.	❑
Outcomes	Depression or peripartum depression is the primary outcome measure (self-report, clinician or proxy administered measure of depression)	❑
	Quality of depression measure used has internal consistency and test-retest reliability with a Cronbach's alpha ≥0.70	❑
Study design	Only randomized controlled trials	❑
	Quality of randomization procedure based on Risk of Bias tool 2.0. [57]<br><br>*Exclusion criteria:* Non-random sequence generation (high risk of bias)<br><br>*Exclusion criteria:* Designs in which allocation sequence is not concealed (before participants are enrolled and assigned to interventions) (high risk of bias)	❑<br><br>❑

Overall decision		
Include	Exclude	Review
❑	❑	❑

Notes:

For peer review only

Table 2: Article allocation in Endnote

# Endnote	Allocation	Description
0	Include	Article in line with PICOS or does not detail enough information to warrant exclusion
1	Exclude	Article clearly not in line with PICOS
2	Systematic review	Article is a systematic review in line with the PICOS
3	Meta-analysis	Article is a meta-analysis in line with the PICOS
4	Review	Article is a review of relevant literature
5	Protocol	Article is a study protocol in line with PICOS
6	Qualitative	Article is a qualitative paper in line with PICOS
7	Other	Article is useful to researcher for another purpose

For peer review only

Search strategies – electronic databases

Medline via Pubmed 1996-present - Provider: United States National Library of Medicine (NLM) – Date:		
**#**	**Searches**	**Results**
**#1**	**postpartum [Title/Abstract] OR<br>post-partum [Title/Abstract] OR<br>antepartum [Title/Abstract] OR<br>ante-partum [Title/Abstract] OR<br>partum [Title/Abstract] OR<br>prepartum [Title/Abstract] OR<br>pre-partum [Title/Abstract] OR<br>intrapartum [Title/Abstract] OR<br>intra-partum [Title/Abstract] OR<br>peripartum [Title/Abstract] OR<br>peri-partum[Title/Abstract] OR<br>postnatal [Title/Abstract] OR<br>post-natal [Title/Abstract] OR<br>perinatal [Title/Abstract] OR<br>peri-natal[Title/Abstract] OR<br>antenatal [Title/Abstract] OR<br>ante-natal [Title/Abstract] OR<br>prenatal [Title/Abstract] OR<br>pre-natal [Title/Abstract] OR<br>pregnant [Title/Abstract] OR<br>pregnancy [Title/Abstract] OR<br>pregnancies [Title/Abstract] OR<br>puerper*[Title/Abstract] OR<br>maternal [Title/Abstract] OR<br>trimester [Title/Abstract] OR<br>impregnated [Title/Abstract] OR<br>gravid* [Title/Abstract] OR<br>multigravid* [Title/Abstract] OR<br>primigravid* [Title/Abstract] OR<br>parity [Title/Abstract] OR<br>obstetric [Title/Abstract] OR<br>gestation [Title/Abstract] OR<br>“in utero” [Title/Abstract] OR<br>maternity [Title/Abstract] OR<br>partus [Title/Abstract] OR<br>obstetrical [Title/Abstract]**	
**#2**	**pregnancy [Mesh] OR<br>postpartum period [Mesh]**	
**#3**	**#1 OR #2**	
**#4**	**depression [Title/Abstract] OR<br>depressed [Title/Abstract] OR<br>depressive [Title/Abstract] OR<br>“low mood” [Title/Abstract] OR<br>mood [Title/Abstract] OR**	

For peer review only

Search strategies – electronic databases

	distress [Title/Abstract] OR<br>wellbeing [Title/Abstract] OR<br>“well being” [Title/Abstract] OR<br>emotion [Title/Abstract] OR<br>emotional [Title/Abstract] OR<br>melanchol* [Title/Abstract] OR<br>affect [Title/Abstract] OR<br>affective [Title/Abstract] OR<br>dysphori* [Title/Abstract] OR<br>dysthymia [Title/Abstract] OR<br>alexithymia [Title/Abstract]	
#5	affect [MeSH Terms] OR<br>mood disorders [MeSH Terms] OR<br>depression [MeSH Terms] OR<br>depressive disorder[MeSH Terms] OR<br>affective symptoms [MeSH Terms] OR<br>affective disorder [MeSH Terms] OR<br>depression, postpartum [MeSH Terms] OR<br>prenatal Care/psychology [MeSH Terms] OR<br>perinatal Care/psychology [MeSH Terms]) OR<br>postnatal Care/psychology [MeSH Terms] OR<br>pregnancy Complications/psychology [MeSH Terms] OR<br>pregnancy Complications/therapy [MeSH Terms]	
#6	#4 OR #5	
#7	cognitive [Title/Abstract] OR<br>behaviour [Title/Abstract] OR<br>behavioural [Title/Abstract] OR<br>behaviour [Title/Abstract] OR<br>behavioural [Title/Abstract] OR<br>cognitive behavio* [Title/Abstract] OR<br>“behavioural activation” [Title/Abstract] OR<br>“behavioral activation” [Title/Abstract] OR<br>“problem solving” [Title/Abstract] OR<br>ccbt [Title/Abstract] OR<br>icbt [Title/Abstract] OR<br>“cognitive restructuring” [Title/Abstract] OR<br>“cognitive reframing” [Title/Abstract] OR<br>“activity scheduling” [Title/Abstract]	
#8	behavior therapy [MeSH Terms] OR<br>cognitive therapy [MeSH Terms] OR<br>behavior therapies, cognitive [MeSH Terms]	
#9	#7 OR #8	
#10	therapy [Title/Abstract] OR<br>therapies [Title/Abstract] OR<br>psychotherapy [Title/Abstract] OR<br>intervention [Title/Abstract] OR<br>management [Title/Abstract] OR	

For peer review only

Search strategies – electronic databases

	"program evaluation" [Title/Abstract] OR<br>program [Title/Abstract] OR<br>programs [Title/Abstract] OR<br>programme [Title/Abstract] OR<br>programmes [Title/Abstract] OR<br>group [Title/Abstract] OR<br>course [Title/Abstract] OR<br>online [Title/Abstract] OR<br>internet [Title/Abstract] OR<br>web [Title/Abstract] OR<br>"web based" [Title/Abstract] OR<br>phone [Title/Abstract] OR<br>telephone [Title/Abstract] OR<br>skype [Title/Abstract] OR<br>e-therapy [Title/Abstract] OR<br>etherapy [Title/Abstract] OR<br>"computer assisted" [Title/Abstract]OR<br>"internet intervention" [Title/Abstract] OR<br>computer [Title/Abstract] OR<br>computerised [Title/Abstract] OR<br>computerized [Title/Abstract] OR<br>mobile [Title/Abstract] OR<br>tablet [Title/Abstract] OR<br>smartphone [Title/Abstract] OR<br>"internet administered" [Title/Abstract] OR<br>"e-mental health" [Title/Abstract] OR<br>"m-mental health" [Title/Abstract] OR<br>Ehealth [Title/Abstract] OR<br>"e-health" [Title/Abstract] OR<br>"e-intervention" [Title/Abstract]	
#11	prenatal Care/methods [Mesh]<br>perinatal Care/methods [Mesh]<br>postnatal Care/methods [Mesh]	
#12	#10 OR #11	
#13	"randomized controlled trial" [Title/Abstract] OR<br>"randomized control trial" [Title/Abstract] OR<br>RCT [Title/Abstract] OR<br>controlled [Title/Abstract] OR<br>randomised [Title/Abstract] OR<br>randomized [Title/Abstract] OR<br>randomisation [Title/Abstract] OR<br>randomization [Title/Abstract] OR<br>"random assignment" [Title/Abstract] OR<br>"random allocation"[Title/Abstract] OR<br>random [Title/Abstract] OR<br>randomly [Title/Abstract] OR<br>control [Title/Abstract] OR	

For peer review only

Search strategies – electronic databases

	feasibility [Title/Abstract] OR<br>pilot [Title/Abstract] OR<br>“comparative study” [Title/Abstract] OR<br>“follow up” [Title/Abstract] OR<br>meta-analysis [Title/Abstract] OR<br>“meta analysis” [Title/Abstract] OR<br>review [Title/Abstract]	
#14	allocation, random [MeSH Terms] OR<br>randomized controlled trial [MeSH Terms] OR<br>meta analysis [MeSH Terms] OR<br>randomized controlled trials as topic [MeSH Terms] OR<br>meta analysis as topic [MeSH Terms] OR<br>review, systematic [MeSH Terms]	
#15	randomized controlled trial [Publication Type] OR systematic review [Publication Type] OR<br>controlled clinical trial [Publication Type] OR<br>meta-analysis [Publication Type]	
#16	#13 OR #14 OR #15	
#17	#3 AND #6 AND #9 AND #12 AND #16	
#17 syntax	((postpartum[Title/Abstract]) OR post-partum[Title/Abstract]) OR antepartum[Title/Abstract]) OR ante-partum[Title/Abstract]) OR partum[Title/Abstract]) OR prepartum[Title/Abstract]) OR pre-partum[Title/Abstract]) OR intrapartum[Title/Abstract]) OR intra-partum[Title/Abstract]) OR peripartum[Title/Abstract]) OR peri-partum[Title/Abstract]) OR postnatal[Title/Abstract]) OR post-natal[Title/Abstract]) OR perinatal[Title/Abstract]) OR peri-natal[Title/Abstract]) OR antenatal[Title/Abstract]) OR ante-natal[Title/Abstract]) OR prenatal[Title/Abstract]) OR pre-natal[Title/Abstract]) OR pregnant[Title/Abstract]) OR pregnancy[Title/Abstract]) OR pregnancies[Title/Abstract]) OR puerper*[Title/Abstract]) OR maternal[Title/Abstract]) OR trimester[Title/Abstract]) OR impregnated[Title/Abstract]) OR gravid*[Title/Abstract]) OR multigravid*[Title/Abstract]) OR primigravid*[Title/Abstract]) OR parity[Title/Abstract]) OR obstetric[Title/Abstract]) OR gestation[Title/Abstract]) OR "in utero"[Title/Abstract]) OR maternity[Title/Abstract]) OR partus[Title/Abstract]) OR obstetrical[Title/Abstract])) OR ((Pregnancy[MeSH Terms]) OR postpartum period[MeSH Terms])))) AND ((((((((((((((((((depression[Title/Abstract]) OR depressed[Title/Abstract]) OR depressive[Title/Abstract]) OR "low mood"[Title/Abstract]) OR mood[Title/Abstract]) OR distress[Title/Abstract]) OR wellbeing[Title/Abstract]) OR "well being"[Title/Abstract]) OR emotion[Title/Abstract]) OR emotional[Title/Abstract]) OR melanchol*[Title/Abstract]) OR affect[Title/Abstract]) OR affective[Title/Abstract]) OR dysphori*[Title/Abstract]) OR dysthymia[Title/Abstract]) OR	TOTAL:

Search strategies – electronic databases

	**alexithymia[Title/Abstract])) OR ((((((((((((affect[MeSH Terms]) OR mood disorders[MeSH Terms]) OR depression[MeSH Terms]) OR depressive disorder[MeSH Terms]) OR affective symptoms[MeSH Terms]) OR affective disorder[MeSH Terms]) OR depression, postpartum[MeSH Terms]) OR Prenatal Care/psychology[MeSH Terms]) OR Perinatal Care/psychology[MeSH Terms]) OR Postnatal Care/psychology[MeSH Terms]) OR Pregnancy Complications/psychology[MeSH Terms]) OR Pregnancy Complications/therapy[MeSH Terms])))) AND (((((((((((((((cognitive[Title/Abstract]) OR behavior[Title/Abstract]) OR behavioral[Title/Abstract]) OR behaviour[Title/Abstract]) OR behavioural[Title/Abstract]) OR cognitive behavio*[Title/Abstract]) OR "behavioural activation"[Title/Abstract]) OR "behavioral activation"[Title/Abstract]) OR "problem solving"[Title/Abstract]) OR ccbt[Title/Abstract]) OR icbt[Title/Abstract]) OR "cognitive restructuring"[Title/Abstract]) OR "cognitive reframing"[Title/Abstract]) OR "activity scheduling"[Title/Abstract])) OR (((behavior therapy[MeSH Terms]) OR cognitive therapy[MeSH Terms]) OR behavior therapies, cognitive[MeSH Terms])))) AND ((((((((((((((((((((((((((((((((((((therapy[Title/Abstract]) OR therapies[Title/Abstract]) OR psychotherapy[Title/Abstract]) OR intervention[Title/Abstract]) OR Management[Title/Abstract]) OR "program evaluation"[Title/Abstract]) OR program[Title/Abstract]) OR programs[Title/Abstract]) OR programme[Title/Abstract]) OR programmes[Title/Abstract]) OR group[Title/Abstract]) OR course[Title/Abstract]) OR online[Title/Abstract]) OR Internet[Title/Abstract]) OR web[Title/Abstract]) OR "web based"[Title/Abstract]) OR phone[Title/Abstract]) OR telephone[Title/Abstract]) OR skype[Title/Abstract]) OR e-therapy[Title/Abstract]) OR etherapy[Title/Abstract]) OR "computer assisted"[Title/Abstract]) OR "internet intervention"[Title/Abstract]) OR computer[Title/Abstract]) OR computerised[Title/Abstract]) OR computerized[Title/Abstract]) OR mobile[Title/Abstract]) OR tablet[Title/Abstract]) OR smartphone[Title/Abstract]) OR "internet administered"[Title/Abstract]) OR e-mental health[Title/Abstract]) OR m-mental health[Title/Abstract]) OR Ehealth[Title/Abstract]) OR e-health[Title/Abstract])) OR e-intervention[Title/Abstract])) OR (((Prenatal Care/methods[MeSH Terms]) OR Perinatal Care/methods[MeSH Terms]) OR Postnatal Care/methods[MeSH Terms]))) AND (((((((randomized controlled trial[Publication Type]) OR systematic review[Publication Type]) OR controlled clinical trial[Publication Type]) OR meta-analysis[Publication Type])) OR ((((((((((((((((((("randomized controlled trial"[Title/Abstract]) OR "randomized control trial"[Title/Abstract]) OR RCT[Title/Abstract]) OR controlled[Title/Abstract]) OR randomised[Title/Abstract]) OR**	

Search strategies – electronic databases

	**randomized[Title/Abstract]) OR randomisation[Title/Abstract]) OR randomization[Title/Abstract]) OR "random assignment"[Title/Abstract]) OR "random allocation"[Title/Abstract]) OR random[Title/Abstract]) OR randomly[Title/Abstract]) OR control[Title/Abstract]) OR feasibility[Title/Abstract]) OR pilot[Title/Abstract]) OR "comparative study"[Title/Abstract]) OR "follow up"[Title/Abstract]) OR meta-analysis[Title/Abstract]) OR "meta analysis"[Title/Abstract]) OR Review[Title/Abstract])) OR ((((((allocation, random[MeSH Terms]) OR randomized controlled trial[MeSH Terms]) OR meta analysis[MeSH Terms]) OR randomized controlled trials as topic[MeSH Terms]) OR meta analysis as topic[MeSH Terms]) OR review, systematic[MeSH Terms]))**	

ISI Web of Science Core Collection (1900-present). Including Conference Proceedings Citation Index- Science (CPCI-S) -1990-present Conference Proceedings Citation Index- Social Science & Humanities (CPCI-SSH) -1990-Present via Clarivate Analytics- Date:	
**Search**	**Results**
TI=(postpartum OR post-partum OR antepartum OR ante-partum OR partum OR prepartum OR pre-partum OR intrapartum OR intra-partum OR peripartum OR peri-partum OR postnatal OR post-natal OR perinatal OR peri-natal OR antenatal OR ante-natal OR prenatal OR pre-natal OR pregnant OR pregnancy OR pregnancies OR puerper* OR maternal OR trimester OR impregnated OR gravid* OR multigravid* OR primigravid* OR parity OR obstetric OR gestation OR “in utero” OR maternity OR partus OR obstetrical) AND TI=(depression OR depressed OR depressive OR “low mood” OR mood OR distress OR wellbeing OR well-being OR emotion OR emotional OR melanchol* OR affect OR affective OR dysphori* OR dysthymia OR alexithymia) AND TI=(cognitive OR behaviour OR behavioural OR behaviour OR behavioural OR “cognitive behavio*” OR “behavioural activation” OR “behavioral activation” OR “problem solving” OR ccbt OR icbt OR “cognitive restructuring” OR “cognitive reframing” OR “activity scheduling”) AND TI=(therapy OR therapies OR psychotherapy OR intervention OR management OR “program evaluation” OR program OR programs OR programme OR programmes OR group OR course OR online OR internet OR web OR web-based OR phone OR telephone OR skype OR e-therapy OR etherapy OR “computer assisted” OR “internet intervention” OR computer OR computerised OR computerized OR mobile OR tablet OR smartphone OR “internet administered” OR “e-mental health” OR “m-mental health” OR Ehealth OR e-health OR e-intervention) AND TI=(“randomized controlled trial” OR “randomized control trial” OR RCT OR controlled OR randomised OR randomized OR randomisation OR randomization OR “random assignment” OR “random allocation” OR random OR randomly OR control OR feasibility OR pilot OR “comparative study” OR “follow up” OR meta-analysis OR “meta analysis” OR review) OR AB=(postpartum OR post-partum OR antepartum OR	

For peer review only

Search strategies – electronic databases

ante-partum OR partum OR prepartum OR pre-partum OR intrapartum OR intra-partum OR peripartum OR peri-partum OR postnatal OR post-natal OR perinatal OR peri-natal OR antenatal OR ante-natal OR prenatal OR pre-natal OR pregnant OR pregnancy OR pregnancies OR puerper* OR maternal OR trimester OR impregnated OR gravid* OR multigravid* OR primigravid* OR parity OR obstetric OR gestation OR “in utero” OR maternity OR partus OR obstetrical) AND AB=(depression OR depressed OR depressive OR “low mood” OR mood OR distress OR wellbeing OR well-being OR emotion OR emotional OR melanchol* OR affect OR affective OR dysphori* OR dysthymia OR alexithymia) AND AB=(cognitive OR behaviour OR behavioural OR behaviour OR behavioural OR “cognitive behavio*” OR “behavioural activation” OR “behavioral activation” OR “problem solving” OR ccbt OR icbt OR “cognitive restructuring” OR “cognitive reframing” OR “activity scheduling”) AND AB=(therapy OR therapies OR psychotherapy OR intervention OR management OR “program evaluation” OR program OR programs OR programme OR programmes OR group OR course OR online OR internet OR web OR web-based OR phone OR telephone OR skype OR e-therapy OR etherapy OR “computer assisted” OR “internet intervention” OR computer OR computerised OR computerized OR mobile OR tablet OR smartphone OR “internet administered” OR “e-mental health” OR “m-mental health” OR Ehealth OR e-health OR e-intervention) AND AB=(“randomized controlled trial” OR “randomized control trial” OR RCT OR controlled OR randomised OR randomized OR randomisation OR randomization OR “random assignment” OR “random allocation” OR random OR randomly OR control OR feasibility OR pilot OR “comparative study” OR “follow up” OR meta-analysis OR “meta analysis” OR review)<br>*Indexes=SCI-EXPANDED, SSCI, A&HCI, CPCI-S, CPCI-SSH, ESCI Timespan=All years*	

Cumulative Index to Nursing and Allied Health Literature (CINAHL) via EBSCO host - 1981-Present - Date:	
**Search**	**Results**
TI (postpartum OR post-partum OR antepartum OR ante-partum OR partum OR prepartum OR pre-partum OR intrapartum OR intra-partum OR peripartum OR peri-partum OR postnatal OR post-natal OR perinatal OR peri-natal OR antenatal OR ante-natal OR prenatal OR pre-natal OR pregnant OR pregnancy OR pregnancies OR puerper* OR maternal OR trimester OR impregnated OR gravid* OR multigravid* OR primigravid* OR parity OR obstetric OR gestation OR “in utero” OR maternity OR partus OR obstetrical OR MH "Pregnancy") AND TI (depression OR depressed OR depressive OR “low mood” OR mood OR distress OR wellbeing OR “well being” OR emotion OR emotional OR melanchol* OR affect OR affective OR dysphori* OR dysthymia OR alexithymia OR MH "Depression, Postpartum" OR MH "Depression") AND TI (cognitive OR behaviour OR behavioural OR behaviour OR behavioural OR cognitive W2 behavio* OR “behavioural activation” OR “behavioral activation” OR “problem solving” OR ccbt OR icbt OR “cognitive restructuring” OR “cognitive reframing” OR “activity scheduling” OR MH	

For peer review only

Search strategies – electronic databases

"Cognitive Therapy" OR MH "Behavior Therapy") AND TI (therapy OR therapies OR psychotherapy OR intervention OR management OR “program evaluation” OR program OR programs OR programme OR programmes OR group OR course OR online OR internet OR web OR “web based” OR phone OR telephone OR skype OR e-therapy OR etherapy OR “computer assisted” OR “internet intervention” OR computer OR computerised OR computerized OR mobile OR tablet OR smartphone OR “internet administered” OR “e-mental health” OR “m-mental health” OR Ehealth OR “e-health” OR “e-intervention”) AND TI (“randomized controlled trial” OR “randomized control trial” OR RCT OR controlled OR randomised OR randomized OR randomisation OR randomization OR “random assignment” OR “random allocation” OR random OR randomly OR control OR feasibility OR pilot OR “comparative study” OR “follow up” OR meta-analysis OR “meta analysis” OR review OR MH "Randomized Controlled Trials" OR MH "Pilot Studies" OR MH "Meta Analysis" OR MH "Systematic Review") OR AB (postpartum OR post-partum OR antepartum OR ante-partum OR partum OR prepartum OR pre-partum OR intrapartum OR intra-partum OR peripartum OR peri-partum OR postnatal OR post-natal OR perinatal OR peri-natal OR antenatal OR ante-natal OR prenatal OR pre-natal OR pregnant OR pregnancy OR pregnancies OR puerper* OR maternal OR trimester OR impregnated OR gravid* OR multigravid* OR primigravid* OR parity OR obstetric OR gestation OR “in utero” OR maternity OR partus OR obstetrical OR MH "Pregnancy") AND AB (depression OR depressed OR depressive OR “low mood” OR mood OR distress OR wellbeing OR “well being” OR emotion OR emotional OR melanchol* OR affect OR affective OR dysphori* OR dysthymia OR alexithymia OR MH "Depression, Postpartum" OR MH "Depression") AND AB (cognitive OR behaviour OR behavioural OR behaviour OR behavioural OR cognitive W2 behavio* OR “behavioural activation” OR “behavioral activation” OR “problem solving” OR ccbt OR icbt OR “cognitive restructuring” OR “cognitive reframing” OR “activity scheduling” OR MH "Cognitive Therapy" OR MH "Behavior Therapy") AND AB (therapy OR therapies OR psychotherapy OR intervention OR management OR “program evaluation” OR program OR programs OR programme OR programmes OR group OR course OR online OR internet OR web OR “web based” OR phone OR telephone OR skype OR e-therapy OR etherapy OR “computer assisted” OR “internet intervention” OR computer OR computerised OR computerized OR mobile OR tablet OR smartphone OR “internet administered” OR “e-mental health” OR “m-mental health” OR Ehealth OR “e-health” OR “e-intervention”) AND AB (“randomized controlled trial” OR “randomized control trial” OR RCT OR controlled OR randomised OR randomized OR randomisation OR randomization OR “random assignment” OR “random allocation” OR random OR randomly OR control OR feasibility OR pilot OR “comparative study” OR “follow up” OR meta-analysis OR “meta analysis” OR review OR MH "Randomized Controlled Trials" OR MH "Pilot Studies" OR MH "Meta Analysis" OR MH "Systematic Review")	

For peer review only

Search strategies – electronic databases

Cochrane Central Register of Controlled Trials (CENTRAL) 1996-present – Date:		
**#**	**Searches**	**Results**
#1	(postpartum OR post-partum OR antepartum OR ante-partum OR partum OR prepartum OR pre-partum OR intrapartum OR intra-partum OR peripartum OR peri-partum OR postnatal OR post-natal OR perinatal OR peri-natal OR antenatal OR ante-natal OR prenatal OR pre-natal OR pregnant OR pregnancy OR pregnancies OR puerper* OR maternal OR trimester OR impregnated OR gravid* OR multigravid* OR primigravid* OR parity OR obstetric OR gestation OR "in utero" OR maternity OR partus OR obstetrical):ti,ab,kw	
#2	MeSH descriptor: [Pregnancy] explode all trees	
#3	MeSH descriptor: [Postpartum Period] explode all trees	
#4	#1 OR #2 OR #3	
#5	(depression OR depressed OR depressive OR "low mood" OR mood OR distress OR wellbeing OR "well-being" OR emotion OR emotional OR melanchol* OR affect OR affective OR dysphori* OR dysthymia OR alexithymia):ti,ab,kw	
#6	MeSH descriptor: [Mood Disorders] explode all trees	
#7	MeSH descriptor: [Depression] explode all trees	
#8	MeSH descriptor: [Depressive Disorder] explode all trees	
#9	MeSH descriptor: [Affective Symptoms] explode all trees	
#10	MeSH descriptor: [Depression, Postpartum] explode all trees	
#11	#5 OR #6 OR #7 OR #8 OR #9 OR #10	
#12	(cognitive OR behaviour OR behavioural OR behaviour OR behavioural OR cognitive NEAR/2 behavio* OR "behavioural activation" OR "behavioral activation" OR "problem solving" OR ccbt OR icbt OR "cognitive restructuring" OR "cognitive reframing" OR "activity scheduling"):ti,ab,kw	
#13	MeSH descriptor: [Behavior Therapy] explode all trees	
#14	MeSH descriptor: [Cognitive Therapy] explode all trees	
#15	#12 OR #13 OR #14	
#16	(therapy OR therapies OR psychotherapy OR intervention OR management OR "program evaluation" OR program OR programs OR programme OR programmes OR group OR course OR online OR internet OR web OR "web based" OR phone OR telephone OR skype OR e-therapy OR etherapy OR "computer assisted" OR "internet intervention" OR computer OR computerised OR computerized OR mobile OR tablet OR smartphone OR	

For peer review only

Search strategies – electronic databases

	"internet administered" OR "e-mental health" OR "m-mental health" OR Ehealth OR "e-health" OR "e-intervention"):ti,ab,kw	
#17	("randomized controlled trial" OR "randomized control trial" OR RCT OR controlled OR randomised OR randomized OR randomisation OR randomization OR "random assignment" OR "random allocation" OR random OR randomly OR control OR feasibility OR pilot OR "comparative study" OR "follow up" OR meta-analysis OR "meta analysis" OR review):ti,ab,kw	
#18	MeSH descriptor: [Random Allocation] explode all trees	
#19	MeSH descriptor: [Randomized Controlled Trial] explode all trees	
#20	MeSH descriptor: [Meta-Analysis as Topic] explode all trees	
#21	MeSH descriptor: [Randomized Controlled Trials as Topic] explode all trees	
#22	#17 OR #18 OR #19 OR #20 OR #21	
#23	#4 AND #11 AND #15 AND #16 AND #22	

PROSPERO: International prospective register of systematic reviews – 2011-present Date:	
**Search**	**Results**
postpartum OR post-partum OR antepartum OR ante-partum OR partum OR prepartum OR pre-partum OR intrapartum OR intra-partum OR peripartum OR peri-partum OR postnatal OR post-natal OR perinatal OR peri-natal OR antenatal OR ante-natal OR prenatal OR pre-natal OR pregnant OR pregnancy OR pregnancies OR puerper* OR maternal OR trimester OR impregnated OR gravid* OR multigravid* OR primigravid* OR parity OR obstetric OR gestation OR in utero OR maternity OR partus OR obstetrical AND depression OR depressed OR depressive OR low mood OR mood OR distress OR wellbeing OR well-being OR emotion OR emotional OR melanchol* OR affect OR affective OR dysphori* OR dysthymia OR alexithymia AND cognitive OR behaviour OR behavioural OR behaviour OR behavioural OR cognitive behavio* OR behavioural activation OR behavioral activation OR problem solving OR ccbt OR icbt OR cognitive restructuring OR cognitive reframing OR activity scheduling AND therapy OR therapies OR psychotherapy OR intervention OR management OR program evaluation OR program OR programs OR programme OR programmes OR group OR course OR online OR internet OR web OR web-based OR phone OR telephone OR skype OR e-therapy OR etherapy OR computer-assisted OR internet intervention OR computer OR computerised OR computerized OR mobile OR tablet OR smartphone OR internet-administered OR e-mental health OR m-mental health OR Ehealth OR e-health OR e-intervention AND randomized controlled trial OR randomized control trial OR RCT OR controlled OR randomised OR randomized OR randomisation OR randomization OR random assignment OR	

For peer review only

Search strategies – electronic databases

random allocation OR random OR randomly OR control OR feasibility OR pilot OR comparative study OR follow up:HA	

Excerpta Medica database (Embase) via OVID 1947-present – Date:		
**#**	**Searches**	**Results**
#1	(postpartum OR post-partum OR antepartum OR ante-partum OR partum OR prepartum OR pre-partum OR intrapartum OR intra-partum OR peripartum OR peri-partum OR postnatal OR post-natal OR perinatal OR peri-natal OR antenatal OR ante-natal OR prenatal OR pre-natal OR pregnant OR pregnancy OR pregnancies OR puerper* OR maternal OR trimester OR impregnated OR gravid* OR multigravid* OR primigravid* OR parity OR obstetric OR gestation OR in utero OR maternity OR partus OR obstetrical).ti,ab.	
#2	exp pregnancy/	
#3	postpartum period.mp.	
#4	puerperium/	
#5	#1 OR #2 OR #3 OR #4	
#6	(depression OR depressed OR depressive OR low mood OR mood OR distress OR wellbeing OR well-being OR emotion OR emotional OR melanchol* OR affect OR affective OR dysphori* OR dysthymia OR alexithymia).ti,ab.	
#7	exp mood disorder/	
#8	exp depression/	
#9	exp postnatal depression/	
#10	#6 OR #7 OR #8 OR #9	
#11	(cognitive OR behaviour OR behavioural OR behaviour OR behavioural OR cognitive ADJ2 behavio* OR behavioural activation OR behavioral activation OR problem solving OR ccbt OR icbt OR cognitive restructuring OR cognitive reframing OR activity scheduling).ti,ab.	
#12	exp cognitive behavioral therapy/	
#13	#12 OR #13	
#16	(therapy OR therapies OR psychotherapy OR intervention OR management OR program evaluation OR program OR programs OR programme OR programmes OR group OR	

For peer review only

Search strategies – electronic databases

	course OR online OR internet OR web OR web based OR phone OR telephone OR skype OR e-therapy OR etherapy OR computer assisted OR internet intervention OR computer OR computerised OR computerized OR mobile OR tablet OR smartphone OR internet administered OR e-mental health OR m-mental health OR Ehealth OR e-health OR e-intervention).ti,ab.	
#17	(randomized controlled trial OR randomized control trial OR RCT OR controlled OR randomised OR randomized OR randomisation OR randomization OR random assignment OR random allocation OR random OR randomly OR control OR feasibility OR pilot OR comparative study OR follow up OR meta-analysis OR meta analysis OR review).ti,ab.	
#18	exp randomization/	
#19	controlled clinical trial/	
#20	exp "systematic review"/	
#22	#17 OR #18 OR #19 OR #20 OR #21	
#23	#4 AND #11 AND #15 AND #16 AND #22	

Applied Social Sciences Index & Abstracts (ASSIA) via ProQuest 1987-present – Date:	
**Search**	**Results**
AB("postpartum" OR "post-partum" OR "antepartum" OR "ante-partum" OR "partum" OR "prepartum" OR "pre-partum" OR "intrapartum" OR "intra-partum" OR "peripartum" OR "peri-partum" OR "postnatal" OR "post-natal" OR "perinatal" OR "peri-natal" OR "antenatal" OR "ante-natal" OR "prenatal" OR "pre-natal" OR "pregnant" OR "pregnancy" OR "pregnancies" OR "puerper*" OR "maternal" OR "trimester" OR "impregnated" OR "gravid*" OR "multigravid*" OR "primigravid*" OR "parity" OR "obstetric" OR "gestation" OR “in utero” OR "maternity" OR "partus" OR "obstetrical")OR TI("postpartum" OR "post-partum" OR"antepartum" OR "ante-partum" OR "partum" OR "prepartum" OR "pre-partum" OR "intrapartum" OR "intra-partum" OR "peripartum" OR "peri-partum" OR "postnatal" OR "post-natal" OR "perinatal" OR "peri-natal" OR "antenatal" OR "ante-natal" OR "prenatal" OR "pre-natal" OR "pregnant" OR "pregnancy" OR "pregnancies" OR "puerper*" OR "maternal" OR "trimester" OR "impregnated" OR "gravid*" OR "multigravid*" OR "primigravid*" OR "parity" OR "obstetric" OR "gestation" OR “in utero” OR "maternity" OR "partus" OR "obstetrical") OR MAINSUBJECT.EXACT.EXPLODE("Pregnancy") OR MAINSUBJECT.EXACT.EXPLODE("Postpartum women") AND AB("depression” OR “depressed” OR “depressive” OR “low mood” OR “mood” OR “distress” OR “wellbeing” OR “well-being” OR “emotion” OR “emotional” OR “melanchol*” OR “affect” OR “affective” OR “dysphori*” OR	

For peer review only

Search strategies – electronic databases

"dysthymia" OR "alexithymia") OR TI("depression" OR "depressed" OR "depressive" OR "low mood" OR "mood" OR "distress" OR "wellbeing" OR "well-being" OR "emotion" OR "emotional" OR "melanchol*" OR "affect" OR "affective" OR "dysphori*" OR "dysthymia" OR "alexithymia") AND AB("cognitive" OR "behaviour" OR "behavioural" OR "behaviour" OR "behavioural" OR "cognitive behavio*" OR "behavioural activation" OR "behavioral activation" OR "problem solving" OR "ccbt" OR "icbt" OR "cognitive restructuring" OR "cognitive reframing" OR "activity scheduling") OR TI("cognitive" OR "behaviour" OR "behavioural" OR "behaviour" OR "behavioural" OR "cognitive behavio*" OR "behavioural activation" OR "behavioral activation" OR "problem solving" OR "ccbt" OR "icbt" OR "cognitive restructuring" OR "cognitive reframing" OR "activity scheduling") OR MAINSUBJECT.EXACT.EXPLODE("Cognitive behaviour therapy") AND AB("therapy" OR "therapies" OR "psychotherapy" OR "intervention" OR "management" OR "program evaluation" OR "program" OR "programs" OR "programme" OR "programmes" OR "group" OR "course" OR "online" OR "internet" OR "web" OR "web-based" OR "phone" OR "telephone" OR "skype" OR "e-therapy" OR "etherapy" OR "computer-assisted" OR "internet intervention" OR "computer" OR "computerised" OR "computerized" OR "mobile" OR "tablet" OR "smartphone" OR "internet-administered" OR "e-mental health" OR "m-mental health" OR "Ehealth" OR "e-health" OR "e-intervention") OR TI("therapy" OR "therapies" OR "psychotherapy" OR "intervention" OR "management" OR "program evaluation" OR "program" OR "programs" OR "programme" OR "programmes" OR "group" OR "course" OR "online" OR "internet" OR "web" OR "web-based" OR "phone" OR "telephone" OR "skype" OR "e-therapy" OR "etherapy" OR "computer-assisted" OR "internet intervention" OR "computer" OR "computerised" OR "computerized" OR "mobile" OR "tablet" OR "smartphone" OR "internet-administered" OR "e-mental health" OR "m-mental health" OR "Ehealth" OR "e-health" OR "e-intervention") AND AB("randomized controlled trial" OR "randomized control trial" OR "RCT" OR "controlled" OR "randomised" OR "randomized" OR "randomisation" OR "randomization" OR "random assignment" OR "random allocation" OR "random" OR "randomly" OR "control" OR "feasibility" OR "pilot" OR "comparative study" OR "follow up" OR "meta-analysis" OR "metaanalysis" OR "review") OR TI("randomized controlled trial" OR "randomized control trial" OR "RCT" OR "controlled" OR "randomised" OR "randomized" OR "randomisation" OR "randomization" OR "random assignment" OR "random allocation" OR "random" OR "randomly" OR "control" OR "feasibility" OR "pilot" OR "comparative study" OR "follow up" OR "meta-analysis" OR "metaanalysis" OR "review") OR MAINSUBJECT.EXACT("Randomized controlled trials") OR MAINSUBJECT.EXACT.EXPLODE("Pilot studies") OR MAINSUBJECT.EXACT.EXPLODE("Followup") OR MAINSUBJECT.EXACT.EXPLODE("Meta-analysis")	

For peer review only

Search strategies – electronic databases

Psychinfo via EBSCO host -1967-present Date: check screenshot		
**#**	**Searches**	**Results**
1	(postpartum OR post-partum OR antepartum OR ante-partum OR partum OR prepartum OR pre-partum OR intrapartum OR intra-partum OR peripartum OR peri-partum OR postnatal OR post-natal OR perinatal OR peri-natal OR antenatal OR ante-natal OR prenatal OR pre-natal OR pregnant OR pregnancy OR pregnancies OR puerper* OR maternal OR trimester OR impregnated OR gravid* OR multigravid* OR primigravid* OR parity OR obstetric OR gestation OR in utero OR maternity OR partus OR obstetrical).ti,ab.	
2	*Pregnancy/	
3	1 OR 2	
4	(depression OR depressed OR depressive OR low mood OR mood OR distress OR wellbeing OR well-being OR emotion OR emotional OR melanchol* OR affect OR affective OR dysphori* OR dysthymia OR alexithymia).ti,ab.	
5	*Major Depression/	
6	*Postpartum Depression/	
7	4 OR 5 OR 6	
8	(cognitive OR behaviour OR behavioural OR behaviour OR behavioural OR cognitive behavio* OR behavioural activation OR behavioral activation OR problem solving OR ccbt OR icbt OR cognitive restructuring OR cognitive reframing OR activity scheduling).ti,ab.	
9	*Cognitive Behavior Therapy/	
10	8 OR 9	
11	(therapy OR therapies OR psychotherapy OR intervention OR management OR program evaluation OR program OR programs OR programme OR programmes OR group OR course OR online OR internet OR web OR web-based OR phone OR telephone OR skype OR e-therapy OR etherapy OR computer-assisted OR internet intervention OR computer OR computerised OR computerized OR mobile OR tablet OR smartphone OR internet-administered OR e-mental health OR m-mental health OR Ehealth OR e-health OR e-intervention).ti,ab.	
12	(randomized controlled trial OR randomized control trial OR RCT OR controlled OR randomised OR randomized OR randomisation OR randomization OR random assignment	

For peer review only

Search strategies – electronic databases

	OR random allocation OR random OR randomly OR control OR feasibility OR pilot OR comparative study OR follow up OR meta-analysis OR meta analysis OR review).ti,ab.	
13	3 AND 7 AND 10 AND 11 AND 12	

Scopus 1966-present – Date:	
**Search**	**Results**
TITLE-ABS (postpartum OR post-partum OR antepartum OR ante-partum OR partum OR prepartum OR pre-partum OR intrapartum OR intra-partum OR peripartum OR peri-partum OR postnatal OR post-natal OR perinatal OR peri-natal OR antenatal OR ante-natal OR prenatal OR pre-natal OR pregnant OR pregnancy OR pregnancies OR puerper* OR maternal OR trimester OR impregnated OR gravid* OR multigravid* OR primigravid* OR parity OR obstetric OR gestation OR "in utero" OR maternity OR partus OR obstetrical) AND TITLE-ABS (depression OR depressed OR depressive OR "low mood" OR mood OR distress OR wellbeing OR "well-being" OR emotion OR emotional OR melanchol* OR affect OR affective OR dysphori* OR dysthymia OR alexithymia) AND TITLE-ABS (cognitive OR behaviour OR behavioural OR behaviour OR behavioural OR cognitive W/2 behavio* OR "behavioural activation" OR "behavioral activation" OR "problem solving" OR ccbt OR icbt OR "cognitive restructuring" OR "cognitive reframing" OR "activity scheduling") AND TITLE-ABS (therapy OR therapies OR psychotherapy OR intervention OR management OR "program evaluation" OR program OR programs OR programme OR programmes OR group OR course OR online OR internet OR web OR "web-based" OR phone OR telephone OR skype OR "e-therapy" OR etherapy OR "computer assisted" OR "internet intervention" OR computer OR computerised OR computerized OR mobile OR tablet OR smartphone OR "internet administered" OR "e-mental health" OR "m-mental health" OR Ehealth OR "e-health" OR "e-intervention") AND TITLE-ABS ("randomized controlled trial" OR "randomized control trial" OR RCT OR controlled OR randomised OR randomized OR randomisation OR randomization OR "random assignment" OR "random allocation" OR random OR randomly OR control OR feasibility OR pilot OR "comparative study" OR "follow up" OR meta-analysis OR "meta analysis" OR review)	

Svemed+ via Karolinska Institutet - Date:	
**Search**	**Results**
(postpartum OR post-partum OR antepartum OR ante-partum OR partum OR prepartum OR pre-partum OR intrapartum OR intra-partum OR peripartum OR peri-partum OR postnatal OR post-natal OR perinatal OR peri-natal OR antenatal OR ante-natal OR prenatal OR pre-natal OR pregnant OR pregnancy OR pregnancies OR puerper* OR maternal OR trimester OR impregnated OR gravid* OR multigravid* OR primigravid* OR parity OR obstetric OR gestation OR "in utero" OR maternity OR partus OR obstetrical OR exp: “pregnancy” OR exp: postpartum period”) AND (depression OR depressed OR depressive OR	

For peer review only - http://bmjopen.bmj.com/site/about/guidelines.xhtml

Search strategies – electronic databases

"low mood" OR mood OR distress OR wellbeing OR "well-being" OR emotion OR emotional OR melanchol* OR affect OR affective OR dysphori* OR dysthymia OR alexithymia OR exp: "affect" OR exp: "mood disorders" OR exp: "depression" OR exp: "depressive disorder" OR exp: "affective symptoms" OR exp: "affective disorder" OR exp: "depression, postpartum" OR exp: "prenatal Care/psychology" OR exp: "perinatal Care/psychology" OR exp: "postnatal Care/psychology" OR exp: "pregnancy Complications/psychology" OR exp: "pregnancy Complications/therapy") AND (cognitive OR behaviour OR behavioural OR behaviour OR behavioural OR cognitive behavio* OR "behavioural activation" OR "behavioral activation" OR "problem solving" OR ccbt OR icbt OR "cognitive restructuring" OR "cognitive reframing" OR "activity scheduling" OR exp: "behavior therapy" OR exp: "cognitive therapy" OR exp: "behavior therapies, cognitive")	

For peer review only

PRESS Guideline — Search Submission & Peer Review Assessment

SEARCH SUBMISSION: *THIS SECTION TO BE FILLED IN BY THE SEARCHER*

Searcher: Danelle Pettman **Email:** Danelle.pettman@kbh.uu.se
Reviewer: Professor Alkistis Skalkidou
Date submitted: 12.11.2018 **Date requested by**: 19.11.2018

Systematic Review Title:

Effectiveness and acceptability of cognitive behavioural therapy based interventions for maternal peripartum depression: A systematic review, meta-analysis and thematic synthesis.

This search strategy is …

X	My PRIMARY (core) database strategy — First time submitting a strategy for search question and database
	My PRIMARY (core) strategy — Follow-up review NOT the first time submitting a strategy for search question and database. If this is a response to peer review, itemize the changes made to the review suggestions
	SECONDARY search strategy— First time submitting a strategy for search question and database
	SECONDARY search strategy — NOT the first time submitting a strategy for search question and database. If this is a response to peer review, itemize the changes made to the review suggestions

Database

(i.e., MEDLINE,CINAHL…): *[mandatory]*

Medline

Interface

(i.e., Ovid, EBSCO…): *[mandatory]*

Pubmed

For peer review only - http://bmjopen.bmj.com/site/about/guidelines.xhtml

Additional database searches

Once the primary search strategy is confirmed we will also search; ISI Web of Science; Cumulative Index to Nursing and Allied Health Literature (CINAHL); Cochrane Central Register of Controlled Trials (CENTRAL); Prospero; Excerpta Medica DataBase (EMBASE); Applied Social Sciences Index and Abstracts (ASSIA); Psychinfo; SCOPUS; and SweMed+

Research Question

(Describe the purpose of the search) *[mandatory]*

(1) to examine the effectiveness of CBT based interventions for PPD on symptoms of depression; (2) to examine the effectiveness of CBT based interventions for PPD on secondary outcome measures including; anxiety, stress (individual and perceived parenting stress), parenting (e.g., sensitivity/responsiveness) perceived social support and perceived parental competence; (3) to investigate clinical and methodological moderators potentially associated with effectiveness; and (4) to describe the acceptability of CBT based interventions for PPD including (but not limited to) mothers' experiences and perceptions, satisfaction, barriers and facilitators to intervention use, intervention relevance to mothers' situations and suggestions for improvements; and (5) to identify adaptations to CBT based interventions associated with acceptability.

PICO Format

(Outline the PICOs for your question — i.e., Patient, Intervention, Comparison, Outcome, and Study Design — as applicable)

**P**	Mothers from conception to 1 year postpartum suffering with depression or low mood
**I**	All variants of behavioural, cognitive and cognitive-behavioural therapy
**C**	All comparators will be considered
**O**	Measures of depression or peripartum depression
**S**	Randomised control trials

For peer review only - http://bmjopen.bmj.com/site/about/guidelines.xhtml

Inclusion Criteria

(List criteria such as age groups, study designs, etc., to be included) *[optional]*

Participants. Adult women with either a diagnosis of PDD, or reporting some level of depression symptomatology with a peripartum onset using a validated tool (e.g., Edinburgh Postnatal Depression Scale (EPDS).

Interventions. CBT, behavioural activation (BA) and problem-solving interventions explicitly targeting an improvement in peripartum depression as a primary outcome.

Comparators. (1) no-treatment control; (2) wait-list control; (3) treatment-as-usual (TAU); (4) non-specific factors component control; (5) specific factors component control; and (6) active comparator

Outcomes: Primary outcomes of interest are self-report, clinician or proxy administered standardised measurement of depression or peripartum depession. Secondary outcomes of interest are standardised measures of (1) anxiety; (2) individual stress; (3) perceived parental stress; (4) self-report parenting; (5) perceived social support; and (6) parental competence.

Study design. Only randomized control trials will be included.

Exclusion Criteria

(List criteria such as study designs, date limits, etc., to be excluded) *[optional]*

Participants. (a) the treatment of mood disorders other than depression (e.g. bipolar affective disorder); and (b) interventions focussed on prevention of maternal psychopathology in at-risk, but not currently symptomatic mothers.

Only studies in English or Swedish will be included.

Was a search filter applied?

No ■ Yes □

If YES, which one(s) (e.g., Cochrane RCT filter, PubMed Clinical Queries filter)? Provide the source if this is a published filter. *[mandatory if YES to previous question — textbox]*

For peer review only - http://bmjopen.bmj.com/site/about/guidelines.xhtml

Other notes or comments you feel would be useful for the peer reviewer? ***[optional]***

We had considered not including the concept 4 therapy terms, however this reduces the specificity of the search.

Please copy and paste your search strategy here, exactly as run, including the number of hits per line. ***[mandatory]***

For peer review only

For peer review only - http://bmjopen.bmj.com/site/about/guidelines.xhtml

Concept 1	Concept 2	Concept 3	Concept 4	Concept 5
**Perinatal**	**Depression terms**	**CBT**	**Therapy terms**	**RCT**
**Title & Abstract**<br>Postpartum 47725<br>post-partum 11003<br>antepartum 5374<br>ante-partum 423<br>partum 12020<br>prepartum 2077<br>pre-partum 329<br>intrapartum 7905<br>intra-partum 319<br>peripartum 4151<br>peri-partum 160<br>postnatal 96485<br>post-natal 6733<br>Perinatal 65465<br>peri-natal 177<br>antenatal 31533<br>ante-natal 511<br>prenatal 87822<br>pre-natal 1028<br>pregnan* 480609<br>puerper* 14147<br>maternal 231206<br>trimester 49419<br>impregnated 10808<br>gravid* 12698<br>multigravid* 1185<br>primigravid* 4766<br>parity 29634<br>obstetric 39258<br>gestation 109623<br>before birth 3679<br>in utero 25721<br>**Mesh terms**<br>Pregnancy 844017	**Title & Abstract**<br>depress* 416497<br>depressive 102129<br>low mood 621<br>mood 66046<br>distress 99553<br>wellbeing 12032<br>well-being 63718<br>emotion 33746<br>emotional 133450<br>melanchol* 2906<br>affect 589721<br>affective 51447<br>affective symptoms 1647<br>negative affect 8508<br>dysphori*5153<br>affective disorder 6830<br>**Mesh terms**<br>affect 30820<br>mood disorders 112524<br>depression 195007<br>depressive disorder 100595<br>affective symptoms 12245<br>affective disorder 112524	**Title & Abstract**<br>cognitive 306140<br>cognitive behavio* 23129<br>behavio* 1122596<br>CBT 9128<br>BA 25470<br>problem solving 16422<br>ccbt 155<br>icbt 551<br>cognitive restructuring 785<br>cognitive reframing 84<br>activity scheduling 39<br>**Mesh terms**<br>behavior therapy 67509<br>cognitive therapy 24435<br>behavior therapies, cognitive 24435	**Title & Abstract**<br>Therapy 1749047<br>Therapies 248514<br>Psychotherapy 35578<br>Intervention 515758<br>Management 983110<br>program evaluation 6170<br>program 400451<br>programs 255468<br>programme 86763<br>programmes 55208<br>group 2335915<br>course 515471<br>online 86832<br>Internet 45003<br>Web 89875<br>web-based 25325<br>Phone 18507<br>Telephone 52303<br>Skype 273<br>e-therapy 452<br>etherapy 9<br>computer-assisted 24439<br>computerized 67756<br>internet intervention* 462<br>computer* 281642<br>mobile 85365<br>tablet* 48173<br>smartphone* 6968<br>internet-administered 65<br>ementalhealth 26	**Publication type**<br>Randomized controlled trial 470367<br>systematic review 222096<br>Clinical trial 809548<br>controlled clinical trial 558223<br>meta-analysis 93273<br>**Title & Abstract**<br>Randomized controlled trial 57325<br>Randomized control trial 3210<br>RCT 18011<br>Controlled 684318<br>randomi* 557922<br>random assignment 2191<br>random allocation 1516<br>random 239928<br>randomly 299405<br>control 2340336<br>controls 775814<br>feasibility 154608<br>pilot 132357<br>comparative study 72223<br>follow up 852728<br>meta-analysis 116966<br>metaanalysis 1531<br>Review 1392398<br>**Mesh terms**<br>allocation, random 96464<br>randomized controlled trial 121644<br>meta analysis 17054<br>randomized controlled trials as topic 121644<br>meta analysis as topic 17054<br>review, systematic 0

For peer review only - http://bmjopen.bmj.com/site/about/guidelines.xhtml

postpartum period 59467<br>prenatal care 24767<br>perinatal care 8957<br>care, postnatal 5085<br>pregnancy complications 400382<br>depression, postpartum 4780			e-mental health 175<br>m-mental health 3<br>Mmentalhealth 1<br>Ehealth 2629<br>e-health 2356	
**Total for concept 1: 1230102**	**Total for concept 2: 1670493**	**Total for concept 3: 1414869**	**Total for concept 4: 6226286**	**Total for concept 5: 6135718**
				**Total for all: 3917**

Syntax:

(((((((((((((((((((((((((((((((((((((postpartum[Title/Abstract]) OR post-partum antepartum[Title/Abstract]) OR ante-partum partum[Title/Abstract]) OR prepartum[Title/Abstract]) OR pre-partum[Title/Abstract]) OR intrapartum[Title/Abstract]) OR intra-partum[Title/Abstract]) OR peripartum[Title/Abstract]) OR peri-partum[Title/Abstract]) OR postnatal[Title/Abstract]) OR post-natal[Title/Abstract]) OR Perinatal[Title/Abstract]) OR peri-natal[Title/Abstract]) OR antenatal[Title/Abstract]) OR ante-natal[Title/Abstract]) OR prenatal[Title/Abstract]) OR pre-natal[Title/Abstract]) OR pregnan*[Title/Abstract]) OR puerper[Title/Abstract]) OR puerper*[Title/Abstract]) OR maternal[Title/Abstract]) OR trimester[Title/Abstract]) OR impregnated[Title/Abstract]) OR gravid*[Title/Abstract]) OR multigravid*[Title/Abstract]) OR primigravid*[Title/Abstract]) OR parity[Title/Abstract]) OR obstetric[Title/Abstract]) OR gestation[Title/Abstract]) OR before birth[Title/Abstract]) OR in utero[Title/Abstract])) OR (((((((pregnancy[MeSH Terms]) OR postpartum period[MeSH Terms]) OR prenatal care[MeSH Terms]) OR perinatal care[MeSH Terms]) OR care, postnatal[MeSH Terms]) OR pregnancy complications[MeSH Terms]) OR depression, postpartum[MeSH Terms])))) AND ((((((((((((((((((depress*[Title/Abstract]) OR depressive[Title/Abstract]) OR low mood[Title/Abstract]) OR mood[Title/Abstract]) OR distress[Title/Abstract]) OR wellbeing[Title/Abstract]) OR well being[Title/Abstract]) OR emotion[Title/Abstract]) OR emotional[Title/Abstract]) OR melanchol*[Title/Abstract]) OR affect[Title/Abstract]) OR affective[Title/Abstract]) OR affective symptoms[Title/Abstract]) OR negative affect[Title/Abstract]) OR dysphori*[Title/Abstract]) OR affective disorder[Title/Abstract])) OR ((((((affect[MeSH Terms]) OR mood disorders[MeSH Terms]) OR depression[MeSH Terms]) OR depressive disorder[MeSH Terms]) OR affective symptoms[MeSH Terms]) OR affective disorder[MeSH Terms]))) AND ((((((((((((cognitive[Title/Abstract]) OR cognitive behavio*[Title/Abstract]) OR behavio*[Title/Abstract]) OR CBT[Title/Abstract]) OR BA[Title/Abstract]) OR problem solving[Title/Abstract]) OR ccbt[Title/Abstract]) OR icbt[Title/Abstract]) OR cognitive

restructuring[Title/Abstract]) OR cognitive reframing[Title/Abstract]) OR activity scheduling[Title/Abstract])) OR (((cognitive behavior therapy[MeSH Terms]) OR behavior therapy[MeSH Terms]) OR cognitive therapy[MeSH Terms]))) AND (((((((((((((((((((((((((((((((((((therapy[Title/Abstract]) OR therapies[Title/Abstract]) OR psychotherapy[Title/Abstract]) OR intervention[Title/Abstract]) OR management[Title/Abstract]) OR program evaluation[Title/Abstract]) OR program[Title/Abstract]) OR programs[Title/Abstract]) OR programme[Title/Abstract]) OR programmes[Title/Abstract]) OR group[Title/Abstract]) OR course[Title/Abstract]) OR online[Title/Abstract]) OR Internet[Title/Abstract]) OR Web[Title/Abstract]) OR web-based[Title/Abstract]) OR Phone[Title/Abstract]) OR Telephone[Title/Abstract]) OR Skype[Title/Abstract]) OR e-therapy[Title/Abstract]) OR Etherapy[Title/Abstract]) OR computer-assisted[Title/Abstract]) OR computerized[Title/Abstract]) OR internet intervention*[Title/Abstract]) OR computer*[Title/Abstract]) OR mobile[Title/Abstract]) OR tablet*[Title/Abstract]) OR smartphone*[Title/Abstract]) OR internet-administered[Title/Abstract]) OR ementalhealth[Title/Abstract]) OR e-mental health[Title/Abstract]) OR m-mental health[Title/Abstract]) OR Mmentalhealth[Title/Abstract]) OR Ehealth[Title/Abstract]) OR e-health[Title/Abstract])) AND (((((((Randomized controlled trial[Publication Type]) OR systematic review[Publication Type]) OR controlled clinical trial[Publication Type]) OR meta-analysis[Publication Type])) OR ((((((((((((((((((Randomized controlled trial[Title/Abstract]) OR Randomized control trial[Title/Abstract]) OR RCT[Title/Abstract]) OR Controlled[Title/Abstract]) OR randomi*[Title/Abstract]) OR random assignment[Title/Abstract]) OR random allocation[Title/Abstract]) OR random[Title/Abstract]) OR randomly[Title/Abstract]) OR control[Title/Abstract]) OR controls[Title/Abstract]) OR feasibility[Title/Abstract]) OR pilot[Title/Abstract]) OR comparative study[Title/Abstract]) OR follow up[Title/Abstract]) OR meta-analysis[Title/Abstract]) OR metaanalysis[Title/Abstract]) OR Review[Title/Abstract])) OR ((((((allocation, random[MeSH Terms]) OR randomized controlled trial[MeSH Terms]) OR meta analysis[MeSH Terms]) OR randomized controlled trials as topic[MeSH Terms]) OR meta analysis as topic[MeSH Terms]) OR review, systematic[MeSH Terms]))

(Add more space, as necessary.)

For peer review only - http://bmjopen.bmj.com/site/about/guidelines.xhtml

PEER REVIEW ASSESSMENT: *THIS SECTION TO BE FILLED IN BY THE REVIEWER*

Reviewer: Professor Alkistis Skalkidou
Date completed: 12/12/2018

1. TRANSLATION

A ---No revisions	☐
B --- Revision(s) suggested	■
C --- Revision(s) required	☐

If "B" or "C," please provide an explanation or example:

Does the search strategy match the research question/PICO?
Yes
Are the search concepts clear?
Yes
Are there too many or too few PICO elements included?
Adequate- but I wonder if the algorithm will work well when you require CBT AND therapy- will it catch something that only has CBT in the text? The word CBT includes the concept of therapy
Same goes for MESH term depression, postpartum AND depression…
Are the search concepts too narrow or too broad?
Ok but sometimes they include each other, see above- Maybe this is not a problem but should be checked.
Does the search retrieve too many or too few records? (Please show number of hits per line.)
As expected
Are unconventional or complex strategies explained?
Yes

2. BOOLEAN AND PROXIMITY OPERATORS

A ---No revisions	☐
B --- Revision(s) suggested	■
C --- Revision(s) required	☐

If "B" or "C," please provide an explanation or example:

For peer review only - http://bmjopen.bmj.com/site/about/guidelines.xhtml

Are Boolean or proximity operators used correctly?
Yes
Is the use of nesting with brackets appropriate and effective for the search?
Yes
If NOT is used, is this likely to result in any unintended exclusions?
-
Could precision be improved by using proximity operators (eg, adjacent, near, within) or phrase searching instead of AND?
Probably, should be checked
Is the width of proximity operators suitable (eg, might adj5 pick up more variants than adj2)?
Not used

3. SUBJECT HEADINGS

A ---No revisions	■
B --- Revision(s) suggested	☐
C --- Revision(s) required	☐

If "B" or "C," please provide an explanation or example:

Are the subject headings relevant?
Yes
Are any subject headings too broad or too narrow?
No but somethings they intercept
Are subject headings exploded where necessary and vice versa?
Yes
Are major headings ("starring" or restrict to focus) used? If so, is there adequate justification?
-
Are subheadings missing?
No
Are subheadings attached to subject headings? (Floating subheadings may be preferred.)
Are not
Are floating subheadings relevant and used appropriately?
Have not seen any used (Subheading [sh:noexp])
Are both subject headings and terms in free text (see the following) used for each concept?
Yes

For peer review only - http://bmjopen.bmj.com/site/about/guidelines.xhtml

4. TEXT WORD SEARCHING

A ---No revisions	■
B --- Revision(s)suggested	☐
C --- Revision(s) required	☐

If "B" or "C," please provide an explanation or example:

Does the search include all spelling variants in free text (eg, UK vs. US spelling)?
Yes
Does the search include all synonyms or antonyms (eg, opposites)?
Perinatal concept include: "Maternity", "Partus" and "Obstetrical"
Depression concept include: "dysthymia" and "alexithymia"
Does the search capture relevant truncation (ie, is truncation at the correct place)?
Yes
Is the truncation too broad or too narrow?
Adequate
Are acronyms or abbreviations used appropriately? Do they capture irrelevant material? Are the full terms also included?
Yes
Have the appropriate fields been searched; for example, is the choice of the text word fields (.tw.) or all fields (.af.) appropriate? Are there any other fields to be included or excluded (database specific)?
OK
Should any long strings be broken into several shorter search statements?

5. SPELLING, SYNTAX, AND LINE NUMBERS

A ---No revisions	■
B --- Revision(s)suggested	☐
C --- Revision(s) required	☐

If "B" or "C," please provide an explanation or example:

Are there any spelling errors?
I have not identified any.
Are there any errors in system syntax; for example, the use of a truncation symbol from a different search interface?
No
Are there incorrect line combinations or orphan lines (ie, lines that are not referred to in the final summation that could indicate an error in an AND or OR statement)?
No

For peer review only - http://bmjopen.bmj.com/site/about/guidelines.xhtml

6. LIMITS AND FILTERS

A ---No revisions	■
B --- Revision(s) suggested	☐
C --- Revision(s) required	☐

If "B" or "C," please provide an explanation or example:

OVERALL EVALUATION (Note: If one or more "revision required" is noted above, the response below must be "revisions required".)

A ---No revisions	☐
B --- Revision(s) suggested	■
C --- Revision(s) required	☐

Additional comments:

Some more comments- look at my review on ART and endometrial cancer, Cochrane, on how we looked at grey literature (additional databases).
I also thought that you have the whole perinatal period as one, and it could be very different effects of interventions for those depressed during and/or after pregnancy- I do not know if you want to already now plan for sensitivity analyses according to timing of intervention and/or maternal depression. Outcome has the same problem- at which time is depression measured? Just a thought.

For peer review only

For peer review only - http://bmjopen.bmj.com/site/about/guidelines.xhtml

Data Extraction Form

Reviewer		
**Study Identification Features**		
Unique Study Identifier		
Title		
Authors		
Year of publication		
Publication type		
Journal name		
Full text or just abstract		
Linked papers		
Contact author information		
Geographical setting		
Funding source		
Language		
**Study Characteristics**		
Aims and objectives		
Design		
Inclusion/exclusion criteria		
Recruitment		
Randomisation	Sequence generation	
	Type	
	Allocation concealment	
	Implementation	
Blinding	Data collectors	
	Data analysts	
**Participant characteristics**		
Age of mother (Mean, SD)		
Age of infant (Mean, SD)		
Point of intervention (e.g., antepartum or postpartum period) ***Moderator***		
Ethnicity		
Relationship status		
Level of educational attainment		
Mothers First child (%Yes/No)		
Average household income		
Breastfeeding (% Yes/No)		
Level of depression severity at baseline (if clinical cut offs available)		
Depression Chronicity years (Mean, SD)		

For peer review only

Intervention Components	
Type of CBT intervention (CBT, BA or problem solving) ***Moderator***	
Inclusion of social components (e.g., peer support group or involvement of partner) -Yes/No ***Moderator***	
Inclusion of parenting intervention components (e.g., video interaction guidance - Yes/No ***Moderator***	
Treatment manual (yes/no)	
Measurement of treatment adherence (yes/no)	
Method of delivery (e.g. face to face, group or internet-administered) ***Moderator***	
Treatment setting (for example, primary care, inpatient)	
Health professional delivering intervention (e.g., clinical psychologist or midwife) ***Moderator***	
Clinicians previous training level (Years; Mean, SD)	
Study specific training (Yes/No & Hours; Mean, SD)	
Duration of treatment (Weeks; Mean, SD)	
Number of sessions (Mean, SD)	
Length of sessions (Minutes; Mean, SD)	
Group size (group based intervention, Mean, SD)	
Type of control condition used (e.g., no-treatment control, wait-list control, TAU, non- specific factors component control, specific factors component control and active comparator) ***Moderator***	
**Outcome Measurements**	
Primary outcome measure	
Quality of primary outcome measure - Cronbach's alpha for internal consistency and test-retest reliability (may need original validation paper)	

Length of follow up (weeks; Mean, SD)	
Length of follow up categorical (e.g., short; post-treament-3 months, medium; 3-6 months, long; 7-11 months; extended; 12 months+) ***Moderator***	
Method of assessment of **anxiety** – (e.g., the Beck Anxiety Inventory)	
Level of **anxiety** severity at baseline (if clinical cut offs available)	
Length of follow up (weeks; Mean, SD)	
Method of assessment of **individual stress** - (e.g., Percieved stress scale)	
Level of **individual stress** severity at baseline (if clinical cut offs available)	
Length of follow up (weeks; Mean, SD)	
Method of assessment of **perceived parental stress** - (e.g., Parenting Stress Index)	
Severity of **perceived parental stress** at Baseline (if clinical cut offs available)	
Length of follow up (weeks; Mean, SD)	
Method of assessment of **self-report parenting** (e.g., sensitivity/responsiveness such as; Postpartum Bonding Questionnaire)	
Severity of **self-report parenting** at Baseline (if clinical cut offs available)	
Length of follow up (weeks; Mean, SD)	
Method of assessment of **perceived social support** (e.g., Multidimensional Scale of Perceived Social Support)	
Severity of **perceived social support** at Baseline (if clinical cut offs available)	
Length of follow up (weeks; Mean, SD)	

For peer review only

Method of assessment of **parental competence - competence** (e.g., Parenting Sense of Competence Scale)	
Severity of **parental competence - competence** at Baseline (if clinical cut offs available)	
Length of follow up (weeks; Mean, SD)	
Method of assessment of **observational parenting** (e.g., sensitivity/responsiveness) measures (e.g., video tapes assessed with mind-mindedness coding manual	
Severity of **observational parenting** at Baseline (if clinical cut offs available)	
Length of follow up (weeks; Mean, SD)	
**Statistical Techniques**	
Power calculation	
Method of dealing with missing data	
Baseline comparability	
**Participant Flow**	
Randomised to intervention	
Randomised to control	
Lost to follow-up intervention (at each time point measured)	
Lost to follow-up control (at each time point measured)	
Analysed intervention (at each time point measured)	
Analysed control (at each time point measured)	
Attrition rate	

Results (for each primary and secondary outcome measurement at each time point)
Where multiple time points are reported, a primary end point ≤6 months post-treatment will be adopted. However, outcomes for all time points reported will be examined to enable a potential moderator analysis on length of follow-up.

Intervention	Outcome measurement	
	Pre-Treatment Means	
	Pre-Treatment SD	
	Pre-Treatment Number Analysed	
	Post-Treatment Means	
	Post-Treatment SD	

For peer review only - http://bmjopen.bmj.com/site/about/guidelines.xhtml

	Post-Treatment Number Analysed	
Control	Outcome measurement	
	Pre-Treatment Means	
	Pre-Treatment SD	
	Pre-Treatment Number Analysed	
	Post-Treatment Means	
	Post-Treatment SD	
**Additional comments**		

For peer review only

BMJ Open

Effectiveness and acceptability of cognitive behavioural therapy based interventions for maternal peripartum depression: a systematic review, meta-analysis and thematic synthesis protocol.

Journal:	*BMJ Open*
Manuscript ID	bmjopen-2019-032659.R1
Article Type:	Protocol
Date Submitted by the Author:	30-Oct-2019

Complete List of Authors:	Pettman, Danelle; Uppsala University, 751 85 Uppsala, Sweden, Clinical Psychology in Healthcare, Department of Women's and Children's Health<br>O'Mahen, Heather; University of Exeter, Perry Road, Devon, EX4 4QG, United Kingdom, Mood Disorders Centre, School of Psychology<br>Svanberg, Agneta ; Uppsala University, 751 85 Uppsala, Sweden, Reproductive Health, Department of Women's and Children's Health<br>von Essen, Louise; Uppsala University, 751 85 Uppsala, Sweden, Clinical Psychology in Healthcare, Department of Women's and Children's Health<br>Axfors, Cathrine; Uppsala University, 751 85 Uppsala, Sweden, Reproductive Health, Department of Women's and Children's Health,<br>Blomberg, Oscar; Uppsala University, 751 85 Uppsala, Sweden, Clinical Psychology in Healthcare, Department of Women's and Children's Health<br>Woodford, Joanne; Uppsala University, 751 85 Uppsala, Sweden, Clinical Psychology in Healthcare, Department of Women's and Children's Health

**Primary Subject Heading**:	Mental health
Secondary Subject Heading:	Mental health, Reproductive medicine
Keywords:	Depression & mood disorders < PSYCHIATRY, PERINATOLOGY, PSYCHIATRY

SCHOLARONE™
Manuscripts

Effectiveness and acceptability of cognitive behavioural therapy based interventions for maternal peripartum depression: a systematic review, meta-analysis and thematic synthesis protocol.

Danelle PETTMAN[1] (danelle.pettman@kbh.uu.se), Heather O'MAHEN[2] (H.OMahen@exeter.ac.uk), Agneta SKOOG SVANBERG[3] (Agneta.Skoog_Svanberg@kbh.uu.se), Louise VON ESSEN[1] (louise-von.essen@kbh.uu.se), Cathrine AXFORS[3] (cathrine.axfors@neuro.uu.se), Oscar BLOMBERG[1], (oscar.blomberg@kbh.uu.se), Joanne WOODFORD[1] (joanne.woodford@kbh.uu.se)

Corresponding author

Dr Danelle Pettman, Clinical Psychology in Healthcare, Department of Women's and Children's Health, Uppsala University, 751 23 Uppsala, Sweden. E-mail: danelle.pettman@kbh.uu.se. Telephone: 0046 18-471 62 58

Affiliations

[1]Clinical Psychology in Healthcare, Department of Women's and Children's Health, Uppsala University, 751 85 Uppsala, Sweden

[2]Mood Disorders Centre, School of Psychology, University of Exeter, Perry Road, Devon, EX4 4QG, United Kingdom,

[3]Reproductive Health, Department of Women's and Children's Health, Uppsala University, 751 85 Uppsala, Sweden

Word Count

(5109 word limit)

For peer review only - http://bmjopen.bmj.com/site/about/guidelines.xhtml

ABSTRACT

Introduction: Peripartum depression is a common mental health difficulty associated with a range of negative impacts for the mother, infant and wider family. This review will examine the effectiveness of CBT based interventions for peripartum depression. Secondary aims are to explore the effect of CBT based interventions targeted at peripartum depression on novel secondary outcomes and moderators potentially associated with effectiveness. To date, there has been little examination of effect on important secondary outcomes (e.g., anxiety, stress and parenting), nor clinical and methodological moderators. Further, this review aims to explore the acceptability of CBT based interventions for women with peripartum depression and examine important adaptations for this population.

Methods and analysis: Electronic databases will be systematically searched. Database searches will be supplemented by expert contact, reference and citation checking, and grey literature. Primary outcomes of interest will be validated measures of symptoms of depression. A proposed meta-analysis will examine: (1) the overall effectiveness of psychological interventions in improving symptoms of depression (both self-reported and diagnosed major depression) in the peripartum period; (2) the impact of interventions on secondary outcomes (e.g., anxiety, stress and parenting); (3) clinical and methodological moderators associated with effectiveness. A thematic synthesis will be conducted on qualitative data exploring the acceptability of CBT based intervention for postpartum depression including participants' experience and perspectives of the interventions, satisfaction, barriers and facilitators to intervention use, intervention relevance to mothers' situations and suggestions for improvements to tailor interventions to the peripartum client group.

For peer review only - http://bmjopen.bmj.com/site/about/guidelines.xhtml

Ethics and dissemination: Formal ethical approval is not required by the National Ethical Review Board in Sweden as primary data will not be collected. The results will be disseminated through a peer-reviewed publication and inform the development of a new psychological intervention for peripartum depression. This study including protocol development will run from March 2019 – March 2020.

STRENGTHS AND LIMITATION OF THIS STUDY

- Systematic review adopts quality standards informed by the Centre of Reviews and Dissemination guidance and PRISMA-P guidelines such as; independent study selection, data extraction and risk of bias assessments by two researchers.
- The review examines the effectiveness of CBT based peripartum depression interventions on secondary outcomes (e.g., anxiety, stress and parenting) as well as clinical and methodological moderators of effectiveness of CBT based interventions for peripartum depression.
- The review is the first to synthesise qualitative data regarding participants experience and perspectives of CBT based interventions for peripartum depression.
- High levels of clinical heterogeneity may exist as a consequence of included studies defining and delivering CBT based interventions in a variety of ways.
- Due to resource limitations, selected studies are only those publically available in the English or Swedish language; therefore, language bias may be present.

For peer review only - http://bmjopen.bmj.com/site/about/guidelines.xhtml

For peer review only

INTRODUCTION

Peripartum depression (PPD) is a common mental health difficulty characterised by low mood during pregnancy (antepartum period) and/or after childbirth (postpartum period).[1] Whilst it has been argued depression occurring during pregnancy is distinctly different to postpartum depression [2] in terms of epidemiological, [3] hormonal sensitivity [4] and immune system functioning [5] for the purposes of this review we will use the more inclusive "peripartum" definition and examine time point of intervention (e.g., antepartum or postpartum period) as a potential moderator of effect.

Prevalence rates of PPD vary dependent on whether measured via screening scales or standardised diagnostic tools, [6] with the former at a rate of 19.2% and the latter with at a rate of 14.5%.[7] A recent study indicates symptoms of depression are more prevalent during the postpartum period than in age matched controls.[8] Further, approximately 40% of women will experience their first depressive episode postpartum,[9] and when left untreated, are more likely to experience further episodes of depression later in life.[10] The impact of PPD is significant for both mothers, infants and the wider family. For mothers PPD is associated with poor health-related quality of life and in extreme cases loss of life through suicide. [11] Further, postpartum depression is associated with increased risk for impairments in the mother's perceived bonding and attachment to the infant, both during the antepartum and postpartum period; in the postpartum period this is related to impairments in maternal responsiveness or sensitivity (hereafter referred to as parenting).[12] Prospectively, PPD is related to impairments in the infant's cognitive, social and emotional functioning.[12,13] Maternal postpartum depression is

For peer review only - http://bmjopen.bmj.com/site/about/guidelines.xhtml

also associated with increased marital discord [14] and depression in fathers.[15] Given the prevalence and impact of PPD, it is important to identify and develop effective interventions.

Existing reviews highlight the effectiveness of a number of interventions for PPD including medication (e.g., antidepressant medication), health promotion interventions (e.g., educational home visits), and psychological interventions (e.g., cognitive behavioural therapy (CBT) and interpersonal therapy (IPT).[16-27] However, the cost/benefit ratio for medication shifts for PPD given unclear evidence concerning safety during pregnancy, [28] and mothers' concerns regarding medication use whilst breastfeeding. [29] Further, women experiencing PPD indicate a preference for psychological interventions over medication.[29] Although there are mixed findings regarding the superiority of one type of psychological intervention over another,[17,25,27] several reviews have found CBT to be an effective psychological intervention for PPD.[23,26,30,31]. As such CBT based interventions are recommended as a first-line intervention in clinical guidelines for a number of common mental health difficulties.[32] Additionally, in comparison with other therapeutic approaches, CBT is provided in a wider range of intervention formats including; individual, [33] group [34] and internet–administered. [35].

Whilst existing interventions have demonstrated CBT based interventions for PPD to be effective for depression they have often neglected important secondary outcomes e.g., anxiety, stress (individual and perceived parenting), parenting (e.g., sensitivity/responsiveness) perceived social support and perceived parental competence has been largely unexamined. It is important to examine the effect of interventions for PPD on symptoms of anxiety given comorbidity rates [36] and research suggesting both maternal anxiety and depression should be addressed in

For peer review only - http://bmjopen.bmj.com/site/about/guidelines.xhtml

interventions.[37] An examination of effect on stress (individual and perceived parenting) is also warranted given the deleterious impact of stress on mothers and infants.[38] Indeed, perceived parenting stress disrupts the ability of mothers to appropriately assess infant signals and to react sensitively to them.[39] Further, research suggests interventions for PPD have little benefit on child outcomes,[40] and therefore both maternal depression and parenting difficulties should be addressed.[41] Additionally, perceived low levels of parental competence [42] and poor social support are associated with PPD suggesting interventions should aim to improve both and be included as a secondary outcome.[43]

A further omission in the current evidence base of interventions for PPD concerns clinical and methodological moderators potentially associated with effectiveness. Whilst a recent review has explored the effect of a variety of moderators on CBT for PPD, including type of study control condition (e.g., wait-list control or treatment-as-usual) and time point of intervention (e.g., antepartum or postpartum)[26] a number of potentially important moderators were not examined. As such, the present review will facilitate the exploration of a number of novel moderators, for example the potential effect of health professional delivering intervention (e.g., psychologists, family doctors, nurses and non-professionals). Investigating the potential impact of these moderators is of importance given CBT based interventions are delivered by a wide range of health professionals. [31] In addition, we will examine the potential effect of including interventions that also include components targeting parenting. This is of particular importance given that research has consistently associated parenting difficulties with postnatal depression.[44] However, to date, to the best of our knowledge, existing reviews have not examined the inclusion of parenting components within CBT based interventions as a moderator.

For peer review only - http://bmjopen.bmj.com/site/about/guidelines.xhtml

Increased understanding of clinical moderators associated with effectiveness are particularly important to inform the development of future interventions for the population.

In addition to the analysis of secondary outcomes and clinical and methodological moderators of the effect of intervention for PPD on the symptoms of depression, further research is also needed to examine intervention acceptability and how barriers to accessing psychological interventions during the peripartum period are addressed. A recent study found low rates of intervention engagement in PPD populations in clinical settings, with up to 86% of those suitable for psychological interventions failing to receive any support.[45] Previous qualitative studies have highlighted both practice (e.g., transportations difficulties, attending appointments around feeding and sleep schedules, childcare needs) and psychological (stigma, sleep deprivation, fear of having child removed) barriers.[23,46] Although there is some evidence suggesting interventions adapted to address these barriers, can improve intervention engagement [19] there is a lack of systematic research investigating how such barriers are addressed by interventions and how uniquely tailoring interventions to the peripartum period may increase engagement and acceptability. As such, we will examine qualitative studies preceding eligible randomised controlled trails (RCTs) regarding intervention development and qualitative studies embedded within RCTs [47] to describe participants' experiences, perspectives and adaptations made to tailor CBT interventions to mothers'.

This review seeks to provide a systematic review of CBT based interventions for PPD. Meta-analysis, data permitting, will be used to examine the effect of CBT on the main outcomes (symptoms of depression and depression diagnosis) and secondary outcomes (e.g., anxiety, stress

For peer review only - http://bmjopen.bmj.com/site/about/guidelines.xhtml

and parenting). In addition, this review will explore the potential clinical and methodological moderators upon the symptoms of depression. Further, data from preceding or embedded qualitative studies [47] will be synthesized to examine women's experiences and perspectives of the interventions. Together this will provide a comprehensive review of the best current evidence regarding CBT based interventions for PPD that can be used to inform future intervention development.

Objectives: (1) to examine the effectiveness of CBT based interventions for PPD on symptoms of depression; (2) to examine the effectiveness of CBT based interventions for PPD on anxiety, stress (individual and perceived parenting stress), parenting (e.g., sensitivity/responsiveness) perceived social support and perceived parental competence; (3) to investigate clinical and methodological moderators potentially associated with effectiveness; and (4) to describe the acceptability of CBT based interventions for PPD including (but not limited to) participants' experience and perspectives of the interventions, satisfaction, barriers and facilitators to intervention use, intervention relevance to mothers' situations and suggestions for improvements; and (5) to identify adaptations to tailor CBT based interventions to mothers' situations potentially associated with acceptability.

For peer review only

METHODS AND ANALYSIS

Design: The protocol for this systematic review has been developed in accordance with the Preferred Reporting Items for Systematic Review and Meta-Analysis Protocols (PRISMA-P)

For peer review only - http://bmjopen.bmj.com/site/about/guidelines.xhtml

2015 statement [48] (see online supplementary appendix 1) and registered with PROSPERO International Prospective Register of Systematic Reviews (#CRDXXXXXX). Protocol amendments will be added to PROSPERO. This systematic review will be conducted in accordance with the Cochrane Collaboration guidance for systematic reviews [49] and reported in accordance with the PRISMA statement. [50]

Patient and public involvement:

Due to project funding constraints patients and members of the public were not involved in the design of this protocol. However, results of the present review will be used to inform the further development of a PPD intervention, following the MRC complex interventions framework [51] targeting both depression and parenting. We will adopt a participatory action research approach [52] to inform the development of the intervention, placing mothers with lived experience of PPD at the centre of the intervention development process.

Inclusion and exclusion criteria: Study inclusion and exclusion criteria were developed using the following domains: Patient; Intervention; Comparison; Outcome; and Study Design (PICOS).[53]

Participants. Adult women aged ≥16 years (a cut-off of 16 years was chosen as studies are likely to be drawn from international settings with variation in the age at which someone is deemed an adult).[54,55] From pregnancy to 12 months postpartum with either; (1) a diagnosis of PPD e.g., Diagnostic and Statistical Manual of Mental Disorders (DSM) IV or V [1,56]; or (2) reporting depression symptomatology within the peripartum period (from pregnancy to 1 year postpartum)

For peer review only - http://bmjopen.bmj.com/site/about/guidelines.xhtml

For peer review only

using a validated tool e.g., Edinburgh Postnatal Depression Scale (EPDS;[57]). No limits will be placed on the severity of depression at baseline. Exclusion criteria are; (1) intervention for mood disorders other than depression (e.g., bipolar affective disorder); and (2) intervention explicitly targeting the prevention of symptoms of depression in at-risk mothers in the perinatal period.[23] For example, a study with the aim to treat current depression during the antepartum period, with an aim to prevent depression during the postpartum period would be eligible for inclusion. However, interventions explicitly targeting the prevention of depression during either the antepartum or postpartum period will be excluded.

Interventions. Only studies investigating interventions explicitly targeting PPD will be included. Eligible interventions will state the use of CBT based interventions, including behavioural activation or problem-solving, and contain specific therapeutic components associated with these approaches. These approaches have been previously synthesised in a meta-analysis for psychotherapy in major depression.[17] CBT will be defined as interventions in which the focus is modifying a client's dysfunctional thoughts on current behaviour and future functioning.[17] BA will be defined as interventions targeting reductions in behavioural avoidance and increases in positively reinforcing activities, including interventions that focus on scheduling behaviours.[58] Problem-solving interventions will be defined as a psychological intervention including the following elements: definition of personal problems, generation of multiple solutions to each problem, selection of the best solution, developing a systematic plan for this solution, and evaluating whether the solution has resolved the problem.[17] There will be no exclusions regarding professional group supporting the intervention, the clinical setting of the intervention, the method of delivery (individual, group or internet-administered), or support

For peer review only - http://bmjopen.bmj.com/site/about/guidelines.xhtml

methods used (e.g., e-mail, face-to-face, telephone). Additionally, self-guided / self-administered interventions will be eligible for inclusion.

Comparators**.** Control conditions eligible for inclusion will be based on standard definitions,[59] with eligible control conditions including: (1) no-treatment control; (2) wait-list control; (3) treatment-as-usual (TAU); (4) non-specific factors component control; (5) specific factors component control; and (6) active comparator. Only trial designs that allow for the isolation of the effects of CBT will be included given it is important for active comparators to discriminate intervention effects.[60] For example, a study comparing CBT alone versus medication alone would be excluded as it would not be possible to isolate the effect of the CBT. However, CBT plus medication versus medication alone and CBT plus information versus information alone would be eligible for inclusion.

Outcomes. This is a mixed methods systematic review involving both quantitative and qualitative outcomes.[61]

Quantitative outcomes. Studies eligible for inclusion will use a self-report or proxy/clinician administered standardised measurement of depression (e.g., the Beck Depression Inventory);[62] or PPD (e.g., EPDS) [57]. In order to ensure the quality of the outcome measurements concerning the primary outcome of depression only studies using depression outcome measurements with at least acceptable internal consistency and test-retest reliability at Cronbach's alpha ≥0.70 (as reported in outcome measurement validation studies) will be included.[63] In addition the proportion of mothers meeting diagnostic criteria for depression

For peer review only - http://bmjopen.bmj.com/site/about/guidelines.xhtml

following intervention will be assessed. In order to ensure quality, only diagnosis made with either the SCID-I [64] or Mini-International Neuropsychiatric Interview (MINI) [65] will be included, as a recent systematic review has highlighted that only these two tools meet sensitivity and specificity criteria.[66] Secondary outcomes of interest are validated self-report measurements of (1) anxiety, (e.g., the Beck Anxiety Inventory);[67] (2) individual stress (e.g., Perceived Stress Scale);[68]; (3) perceived parental stress (e.g., Parenting Stress Index);[69]; (4) self-report parenting (e.g., sensitivity/responsiveness such as; Postpartum Bonding Questionnaire);[70]; (5) perceived social support (e.g., Multidimensional Scale of Perceived Social Support);[71]; and (6) parental competence (e.g., Parenting Sense of Competence Scale).[72] Additionally observational parenting (e.g., sensitivity/responsiveness) measures (e.g., video tapes assessed with mind-mindedness coding manual;[73] will be included as secondary outcome measurements.

Qualitative outcomes. Studies eligible for inclusion will be; (1) qualitative studies preceding RCT studies to develop the intervention(s) examined in RCTs or qualitative studies embedded within RCT studies; and (2) examining acceptability of interventions (e.g., participants' experience and perspectives of the interventions, satisfaction, barriers and facilitators to intervention use, intervention relevance to mothers' situations and suggestions for improvements); and/or (3) examining adaptations made to tailor the intervention to the population. If mixed methods studies are identified using quantitative measures of intervention acceptability (e.g., the Treatment Acceptability/Adherence Scale TAAS; [74]) this data will be extracted and triangulated with the qualitative findings regarding acceptability.

For peer review only - http://bmjopen.bmj.com/site/about/guidelines.xhtml

Study Designs: Both studies with quantitative and qualitative designs will be included.

Quantitative: To examine effectiveness only RCTs will be included, with non-randomised and uncontrolled designs excluded. RCT designs were chosen for this systematic review as they are less prone to biases inherent with other study designs.[75] Randomisation aims to balance known and unknown variables between the treatment groups in order to control for confounding. Random allocation also facilitates the blinding of interventions e.g., to evaluators.[76] Meta-epidemiological studies have shown that trials without proper RCT design, i.e., with inadequate concealment of treatment allocation or inadequate randomization, tend to report higher effect sizes.[77]

Further, RCTs with randomisation procedures at allocation and concealment rated as high risk of bias, in line with the revised Cochrane risk-of-bias tool for randomized trials (RoB 2), [78] will be excluded (see online supplementary appendix 2). This is to minimise the inclusion of studies of low quality with high risk of selection bias known to inflate effect sizes (for example,[79,80]) and is a technique used in previous systematic reviews and meta-analyses.[81–83]

Qualitative: Data from preceding or embedded qualitative studies within included RCTs will be examined for mothers' experiences and perspectives of interventions and potential adaptations of interventions.

Search methods:

For peer review only - http://bmjopen.bmj.com/site/about/guidelines.xhtml

Electronic searches. Searches will be carried out in relevant international electronic databases; MEDLINE; ISI Web of Science; Cumulative Index to Nursing and Allied Health Literature (CINAHL); Cochrane Central Register of Controlled Trials (CENTRAL); Prospero; Excerpta Medica DataBase (EMBASE); Applied Social Sciences Index and Abstracts (ASSIA); PsycINFO; SCOPUS; and SweMed+. Searches will also be conducted on clinical trial registers (www.ClinicalTrials.gov and www.who.int/trialsearch/) and conference proceedings (BIOSIS Previews; Index to Scientific and Technical Proceedings, Health Management Consortium and Web of Science with Conference Proceedings). Databases will be searched using medical subject headings (MeSH) and text words in the title and abstract (see online supplementary appendix 3). The search strategy was developed alongside Agnes Kotka (librarian at Uppsala University Library) and reviewed by Professor Alkistis Skalkidou (Uppsala University) using the PRESS Peer Review Guidelines;[84]; see online supplementary appendix 4). No date limits will be imposed. Studies published in English and Swedish will be accepted.

Other resources. Forward citation searches using Google Scholar (forward citation chasing) [85] will be conducted for all included studies and reference lists of all of the included studies will be hand searched. Studies included within identified secondary evidence reports (e.g., relevant systematic reviews and meta-analyses) will also be reviewed. Systematic reviews will only be included in the hand search if they meet the following criteria; (1) search of at least one database; (2) reporting of selection criteria; (3) a quality appraisal of included studies; and (4) provide a list and synthesis of included studies.[86]

For peer review only - http://bmjopen.bmj.com/site/about/guidelines.xhtml

Additionally, the five journals containing the highest numbers of included studies will be hand searched for recent potentially eligible publications in the past 12 months.[87] Grey literature will be searched using; OpenGrey, a system for grey literature produced in Europe, such as research reports, doctoral dissertations and conference papers (http://www.opengrey.eu/); ProQuest a dissertation and thesis database (http://www.proquest.com/en-US/catalogs/databases/detail/ pqdt.shtml); and DIVA a portal for searching publications from universities managed by Uppsala University. Searches for relevant dissertations will be conducted; however, due to time and funding limitations full dissertations will not be reviewed. The references for potentially eligible dissertations will be made available as part of a list of excluded studies. Potentially relevant studies published in a languages other than English and Swedish will not be included within the review due to lack of resources for translation, and will be provided as part of a list of excluded studies.

Screening and data extraction:

Screening. Duplicates of studies across searches will be identified and removed. Disagreements regarding inclusion will be discussed between the two reviewers, with a third reviewer consulted if consensus cannot be reached. Two independent reviewers will conduct a broad screen of study titles and abstracts, followed by full paper checks of potentially eligible studies. Studies will be excluded that do not clearly meet the outlined PICOS criteria. Overall reasons for exclusion will be recorded (see online supplementary appendix 2) and reported on the PRISMA flow chart in the results manuscript. In addition, a more detailed exclusion table will be presented in the result manuscript with inclusion/exclusion presented for each specific criteria in line with the PICOS statement.

For peer review only - http://bmjopen.bmj.com/site/about/guidelines.xhtml

In the case of studies in which missing data is needed to determine eligibility, authors will be contacted by email for additional information. For authors that do not respond to the first email within two weeks a follow up email will be sent.

Data extraction.

Quantitative extraction. Two reviewers will independently extract data from the included studies following Centre for Reviews and Dissemination (CRD) guidance. [88] Data will be extracted according to data extraction form (see online supplementary appendix 5) including; (1) study characteristics; (2) participant characteristics; (3) intervention characteristics; (4) outcome measurements; and (5) quantitative results. The following data will be extracted:

1. *Study characteristics;* citation, publication type (published or unpublished), country of origin, funding source, language, aims and objectives, design and inclusion/exclusion criteria.

2. *Participant characteristics*; method of depression assessment (e.g., structured clinical interview or screening tool), major depression diagnosis (yes/no), recruitment setting (clinical or community), age of mother (Mean, SD), age of infant (Mean, SD), time point of intervention (e.g., antepartum or postpartum), baseline anxiety, comorbidities, ethnicity of mother (n, %), relationship status (n, %), educational status (n, %), employment status (n, %), mothers first child (n, %), average household income (n, %), breastfeeding (n, %), level of depression severity at baseline (if clinical cut offs available), depression chronicity years (Mean, SD), number of participants invited,

For peer review only - http://bmjopen.bmj.com/site/about/guidelines.xhtml

number of participants screened, number of participants eligible, number of participants randomised and reasons for non-eligibility.

3. *Intervention characteristics*; type of CBT intervention (CBT, BA or problem solving), inclusion of social components (e.g., peer support group or involvement of partner; yes/no), inclusion of parenting intervention components (e.g., video interaction guidance; yes/no), treatment manual (yes/no), measurement of treatment adherence (yes/no), method of delivery (e.g., face to face, group or internet-administered), treatment setting (e.g., clinic or community), health professional delivering intervention (e.g., clinical psychologist or midwife), study specific training (yes/no), duration of treatment (weeks), number of sessions, length of sessions (minutes), maximum length of treatment sessions over treatment course (minutes), group size (Mean, SD) and type of control condition used (e.g., no-treatment control, wait-list control, TAU, non-specific factors component control, specific factors component control and active comparator).

4. *Outcome measurements*; Primary outcome: outcome measure used, participant n, Mean, SD and/or SE for each outcome time point collected and measure of outcome quality (Cronbach alpha for internal consistency and test retest reliability from original validation papers). Secondary outcome(s) (including; anxiety, individual stress, perceived parental stress, self-report parenting, perceived social support, parental competence and observational parenting): outcome measure used, participant n, Mean, SD and/or SE for each outcome time point collected.

5. *Statistical techniques;* power calculation, intention to treat (yes/no), method of dealing with missing data, baseline comparability and for cluster trials, estimates of intra-cluster correlation coefficients (ICC) will be gathered).

For peer review only - http://bmjopen.bmj.com/site/about/guidelines.xhtml

6. *Participant flow*; randomised to intervention, randomised to control, lost to follow-up (at each time point measured), analysed intervention (at each time point measured) and analysed control (at each time point measured) and attrition rate.

7. *Research ethics*; data relating to ethics (e.g., ethical approval, ethical issues highlighted).

Qualitative extraction. Qualitative studies (preceding or embedded within included RCT studies) focused on CBT for PPD intervention acceptability and intervention adaptations will be sought by two reviewers. Data from these studies will be imported into NVivo [89] software for analysis.

Discrepancies will be discussed between the two reviewers, with a third reviewer consulted if consensus cannot be reached. Study authors will be contacted in the event of missing data.

Risk of bias assessment. Methodological quality of included studies will be assessed using the Cochrane Collaboration's Risk of Bias tool 2.0.[78] Two reviewers will work independently to determine the adequacy of (1) randomisation; (2) allocation to intervention; (3) adherence to intervention; (4) handling of missing outcome data; (5) measurement of outcome; and (6) selection of the reported results. Ratings will be compared and any discrepancies discussed, and if consensus is not reached, further discussion will be held with a third reviewer. To assess reporting bias study protocols will be sought for all included studies either via published protocols, trial databases or emailing study authors. Comparisons will be made between outcome measurements reported in the protocol and the paper as well as outcomes reported in the methods and results section of published trial results. Study authors will be contacted to clarify discrepancies and to identify potential changes to the study protocol and request any missing

For peer review only - http://bmjopen.bmj.com/site/about/guidelines.xhtml

data. Consistent with a previous meta-analysis,[82] in order to include quality as a moderator each area addressing risk of bias will be given a score of 0, 1 or 2, indicating bias based on the Risk of Bias tool 2.0,[78] yielding a possible total score of 12. The ratings will be defined as high (9-12), unclear (4-8), or low risk (0-3) respectively. All findings will be summarised within a table to allow easy comparison across studies.

Data synthesis and statistical analysis: Quantitative and qualitative data will be analysed separately.[90] Quantitative data will be managed using an extraction database developed in Microsoft Access for this review and analysed in Comprehensive Meta-analysis (version 2). Qualitative data will be managed and analysed using NVivo10.[89]

Measures of intervention effect. If data allows, a meta-analysis will be conducted. Hedges' g will be calculated to determine the post-treatment between-group standardised mean effect size from outcomes relating to the primary (depression) and secondary outcomes (anxiety, individual stress, perceived parental stress, parenting, perceived social support and parental competence). Where multiple time points are reported, a primary time point of ≤6 months post-treatment will be adopted for the primary analysis to reduce the likelihood of bias associated with examining short-term post-treatment effects only that are likely to result in elevated effect sizes.[81,91,92] However, length of follow-up will be included as a moderator. The control condition sample size will be halved for studies where two interventions eligible for inclusion are compared with one control condition and comparisons will be analysed separately. Additionally, in studies comparing two control conditions with one intervention condition, comparisons will be analysed separately with the sample size in the intervention condition halved [93].

For peer review only - http://bmjopen.bmj.com/site/about/guidelines.xhtml

For peer review only

Dealing with missing data. Study authors will be contacted to provide missing means and standard deviations of post-treatment measurement scores. Intention-to-treat principles stipulate the inclusion of every subject who is randomized according to randomized intervention assignment [94] and will be followed as far as possible. In instances in which intention-to-treat data is not available completer data will be extracted.

Assessment of Heterogeneity. Heterogeneity is anticipated due to variations in interventions in the studies, participant characteristics, and methodological factors, therefore, a random effects model [95] will be adopted with Q and I^2 [96] reported alongside confidence intervals as measures of heterogeneity. If this assumption is not met in the Q and I^2 analysis, a fixed effects model will be used.

Sensitivity analysis. A sensitivity analysis regarding the overall effect size of the primary outcome (depression) will be conducted by removal of (1) each study individually from the overall analysis and the effect size recalculated in order to estimate the statistical validity of the effect size; [97] (2) small studies ($n \leq 20$ across conditions) to explore the suggestion that small trials tend to report larger treatment benefits than larger trials [98]; and/or (3) studies with high attrition rates (≥30% in at least one arm), given attrition rates of ≥30% are associated with imbalances at baseline and are therefore vulnerable to bias such as clinical and psychosocial differences between groups due to differences between those that leave or remain in the study, which is likely to have an impact upon the estimated effect of the exposure. [99]

For peer review only - http://bmjopen.bmj.com/site/about/guidelines.xhtml

Funnel asymmetry. If there is a minimum of 10 studies in any meta-analysis, funnel plot asymmetry will be examined for sources of possible bias (e.g., publication bias, language bias, inclusion of small studies with poor methodological quality, and heterogeneity).[100,101]. Comprehensive Meta-analysis (version 2) will be used to assess possible bias. An estimated effect size taking these biases into account will be calculated using the trim and fill procedure [102], with an estimated effect size calculated separately for each primary and secondary outcome.

Moderator analysis. If sufficient data are available moderator analysis will be undertaken to examine clinical and methodological components and participant characteristics of studies potentially associated with effectiveness. The following moderators will be examined:

Methodological moderators:

- Risk of bias (e.g., high, unclear or low)
- Type of control condition used (e.g., no-treatment control, wait-list control, TAU, non-specific factors component control, specific factors component control and active comparator).[103]
- Length of follow up (e.g., short; post-treatment-<3 months, medium; 3-6 months, long; 7-11 months; extended; 12 months+)

Clinical moderators:

- Severity of depression at baseline (e.g., severe, moderate or mild)
- Type of CBT intervention (e.g., CBT, BA or problem solving)

For peer review only - http://bmjopen.bmj.com/site/about/guidelines.xhtml

- Interventions including additional social components (e.g., peer support group or involvement of partner). Social components will be defined as interventions facilitating contact with peers or partners in a way that encourages nondirective (without specific psychological techniques) support, such as helping people to express their experiences and emotions and offering empathy.
- Interventions including parenting intervention components (e.g., video interaction guidance).[104] Parenting intervention components will be defined as interventions that include specific support in relation to the parent infant relationship this could be via specific sessions with a therapist or via self-help materials**.**
- Method of delivery (e.g., individual, group or internet-administered)
- Time point of intervention (e.g., antepartum or postpartum period)
- Health professional delivering intervention (e.g., clinical psychologist or midwife)

With heterogeneity being anticipated, random effects will be adopted with Q and I^2 reported as measures of heterogeneity.

Qualitative data synthesis. Thematic synthesis will be used to analyse qualitative data.[105] In this review eligible qualitative data will be defined as all text labelled as 'results' or 'findings' in study reports.[105] Similar to analysis of primary qualitative datasets, thematic synthesis involves the systematic coding of data and generating of descriptive and analytical themes. This data will be analysed following a three step process. First, study data will be coded line by line by the first reviewer and checked by a second reviewer with a coding frame developed from codes derived from the data. Second, similar codes will be grouped together to form new

For peer review only - http://bmjopen.bmj.com/site/about/guidelines.xhtml

‘descriptive themes’. Disparities or discrepancies in coding and theme development will be resolved through discussion or in consultation with a third reviewer if necessary; with the coding frame adjusted accordingly. Once coding is complete the two reviewers will consider each theme and sub-theme and examine the text within them for consistency, code interrelations, and identify any potential conceptual hierarchies. Third, analytic themes will be developed to ‘go beyond’ the primary reported data by synthesising findings across studies and interpreting their meaning in relation to review objectives. Narrative descriptions of each theme will be provided, alongside supporting quotations. All changes to codes, conceptual re-alignments, discussions and decisions will be documented as part of the review audit trail. Research supervisors (HOM, JW) have extensive experience in the analysis of qualitative data using a variety of approaches, are fully conversant in the use of NVivo, and will provide supervision to the PhD Student (DP) and Research Assistant (OB) conducting the meta-synthesis.

SUMMARY

This review represents the first review of interventions for PPD that includes mixed methods to examine the overall effectiveness of CBT based interventions (including its impact of secondary outcomes and potential moderators of the effect). The review also examines the acceptability of CBT interventions for PPD and adaptations to potentially improve acceptability of CBT for a PPD population. Conclusions will be drawn in the context of the limitations of the methodology adopted for this review. However the review will provide a necessary step in addressing the current state of the CBT evidence base for PPD and results will have the potential to inform future PPD intervention developments in line with phase I of the Medical Research Council framework for developing complex interventions. [51]

For peer review only - http://bmjopen.bmj.com/site/about/guidelines.xhtml

ETHICS AND DISSEMINATION PLAN

Systematic reviews potentially present two ethical challenges. First, informed consent provided for the original study is not necessarily valid for inclusion of data in the systematic review. Second, systematic reviews may include studies with ethical insufficiencies.[106] However, no raw individual level data is included within this review, with only group level data extracted and analysed, as such no formal ethical approval is required by the National Ethical Review Board in Sweden. In addition, data will be extracted on ethical considerations for each included study with a summary paragraph included in the final manuscript under the heading 'Funding and ethics'.[106]

Dissemination to relevant audiences is a necessary step towards impact. This review will be of relevance to a range of audiences including those working in the peripartum, women's health and CBT fields. We will report review results through scientific conferences and publications, as well as publications for professional and lay audiences and meetings. We will also provide summaries of results and offer to present these for key parties including Health Insurance Directors and National and County Level Health Policy representatives. The authors will use social media and attend participatory involvement events to promote the review results and patient representatives will be contacted to feature results on social media, blogs and vlogs.

REFERENCES

1 American Psychiatric Association. *Diagnostic and statistical manual of mental disorders 5th edition: DSM-V*. Washington DC: American Psychaitric Association 2013.

2 Payne JL. Recent Advances and Controversies in Peripartum Depression. *Curr Obstet*

For peer review only - http://bmjopen.bmj.com/site/about/guidelines.xhtml

Gynecol Rep 2016;**5**:250–6. doi:10.1007/s13669-016-0167-x
3 Vesga-López O, Blanco C, Keyes K, *et al.* Psychiatric disorders in pregnant and postpartum women in the United States. *Arch Gen Psychiatry* 2008;**65**:805–15. doi:10.1001/archpsyc.65.7.805
4 Bloch M, Schmidt PJ, Danaceau M, *et al.* Effects of gonadal steroids in women with a history of postpartum depression. *Am J Psychiatry* 2000;**157**:924–30. doi:10.1176/appi.ajp.157.6.924
5 Sharma V, Mazmanian D. The DSM-5 peripartum specifier: Prospects and pitfalls. *Arch Womens Ment Health* 2014;**17**:171–3. doi:10.1007/s00737-013-0406-3
6 Misri S, Abizadeh J, Nirwin S. Depression during pregnancy and postpartum period. In: Wenzel A, ed. *The Oxford Handbook of Perinatal Psychology*. Oxford University Press 2016. 111–31.
7 Gavin NI, Gaynes BN, Lorh KN, *et al.* Perinatal depression: a systematic review of prevalence and incidence. *Obstet Gynecol* 2005;**106**:1071–83. doi:10.1097/01.AOG.0000183597.31630.db
8 Merkitch KG, Jonas KG, O'Hara MW. Modeling trait depression amplifies the effect of childbearing on postpartum depression. *J Affect Disord* 2017;**223**:69–75. doi:10.1016/j.jad.2017.07.017
9 Woolhouse H, Gartland D, Mensah F, *et al.* Maternal depression from early pregnancy to 4 years postpartum in a prospective pregnancy cohort study: Implications for primary health care. *BJOG An Int J Obstet Gynaecol* 2015;**122**:312–21. doi:10.1111/1471-0528.12837
10 Josefsson A, Sydsjö G. A follow-up study of postpartum depressed women: Recurrent maternal depressive symptoms and child behavior after four years. *Arch Womens Ment Health* 2007;**10**:141–5. doi:10.1007/s00737-007-0185-9
11 Bauer A, Knapp M, Parsonage M. Lifetime costs of perinatal anxiety and depression. *J Affect Disord* 2016;**192**:83–90. doi:10.1016/j.jad.2015.12.005
12 Stein A, Netsi E, Lawrence PJ, *et al.* Mitigating the effect of persistent postnatal depression on child outcomes through an intervention to treat depression and improve parenting: a randomised controlled trial. *Lancet Psychiatry* 2018;**5**:134–44. doi:10.1016/s2215-0366(18)30006-3
13 Netsi E, Pearson RM, Murray L, *et al.* Association of persistent and severe postnatal depression with child outcomes. *JAMA Psychiatry* 2018;**75**:247–53. doi:10.1001/jamapsychiatry.2017.4363
14 Letourneau NL, Dennis CL, Benzies K, *et al.* Postpartum depression is a family affair: Addressing the impact on mothers, fathers, and children. *Issues Ment Health Nurs* 2012;**33**:445–57. doi:10.3109/01612840.2012.673054
15 Massoudi P, Hwang CP, Wickberg B. Fathers' depressive symptoms in the postnatal period: Prevalence and correlates in a population-based Swedish study. *Scand J Public Health* 2016;**44**:688–94. doi:10.1177/1403494816661652
16 Clarke K, King M, Prost A. Psychosocial interventions for perinatal common mental disorders delivered by providers who are not mental health specialists in low- and middle-income countries: A systematic review and meta-analysis. *PLoS Med* 2013;**10**:e1001541. doi:10.1371/journal.pmed.1001541
17 Cuijpers P, van Straten A, Andersson G, *et al.* Psychotherapy for depression in adults: A meta-analysis of comparative outcome studies. *J Consult Clin Psychol* 2008;**76**:909–22.

For peer review only - http://bmjopen.bmj.com/site/about/guidelines.xhtml

doi:10.1037/a0013075

18 Dennis CL. Treatment of postpartum depression, part 2: a critical review of nonbiological interventions. *J Clin Psychiatry* 2004;**65**:1252–65. doi:10.4088/JCP.v65n0915

19 Dennis C-L, Dowswell T. Psychosocial and psychological interventions for preventing postpartum depression. *Cochrane Database Syst Rev* Published Online First: 28 February 2013. doi:10.1002/14651858.CD001134.pub3

20 Dennis C-L, Hodnett ED. Psychosocial and psychological interventions for treating postpartum depression. *Cochrane Database Syst Rev* Published Online First: 17 October 2007. doi:10.1002/14651858.CD006116.pub2

21 Leis JA, Mendelson T, Tandon SD, *et al.* A systematic review of home-based interventions to prevent and treat postpartum depression. *Arch Womens Ment Health* 2009;**12**:3–13. doi:http://0-dx.doi.org.lib.exeter.ac.uk/10.1007/s00737-008-0039-0

22 Morrell CJ, Sutcliffe P, Booth A, *et al.* A systematic review, evidence synthesis and meta-analysis of quantitative and qualitative studies evaluating the clinical effectiveness, the cost-effectiveness, safety and acceptability of interventions to prevent postnatal depression. *Health Technol Assess (Rockv)* 2016;**20**:1–414. doi:10.3310/hta20370

23 Nillni YI, Mehralizade A, Mayer L, *et al.* Treatment of depression, anxiety, and trauma-related disorders during the perinatal period: A systematic review. *Clin Psychol Rev* Published Online First: 2018. doi:10.1016/j.cpr.2018.06.004

24 Scope A, Leaviss J, Kaltenthaler E, *et al.* Is group cognitive behaviour therapy for postnatal depression evidence-based practice? A systematic review. *BMC Psychiatry* 2013;**13**:321. doi:10.1186/1471-244X-13-321

25 Sockol LE, Epperson CN, Barber JP. A meta-analysis of treatments for perinatal depression. *Clin Psychol Rev* 2011;**31**:839–49. doi:10.1016/j.cpr.2011.03.009

26 Sockol LE. A systematic review of the efficacy of cognitive behavioral therapy for treating and preventing perinatal depression. *J Affect Disord* 2015;**177**:7–21. doi:10.1016/j.jad.2015.01.052

27 Stephens S, Ford E, Paudyal P, *et al.* Effectiveness of Psychological Interventions for Postnatal Depression in Primary Care: A Meta-Analysis. *Ann Fam Med* 2016;**14**:463–72. doi:10.1370/afm.1967

28 Tak CR, Job KM, Schoen-Gentry K, *et al.* The impact of exposure to antidepressant medications during pregnancy on neonatal outcomes: a review of retrospective database cohort studies. *Eur J Clin Pharmacol* 2017;**73**:1055–69. doi:10.1007/s00228-017-2269-4

29 Battle CL, Salisbury AL, Schofield CA, *et al.* Perinatal antidepressant use: understanding women's preferences and concerns. *J Psychiatr Pract* 2013;**19**:443–53. doi:10.1097/01.pra.0000438183.74359.46

30 Sockol LE, Epperson CN, Barber JP. A meta-analysis of treatments for perinatal depression. *Clin Psychol Rev* 2011;**31**:839–49. doi:10.1016/j.cpr.2011.03.009

31 Stamou G, García-palacios A, Botella C. Cognitive-Behavioural therapy and interpersonal psychotherapy for the treatment of post-natal depression : a narrative review. *BMC Psychol* 2018;**18**. doi:10.1186/s40359-018-0240-5

32 David D, Cristea I, Hofmann SG. Why Cognitive Behavioral Therapy is the Current Gold Standard of psychotherapy. *Front Psychiatry* 2018;**9**:10–3. doi:10.3389/fpsyt.2018.00004

33 O'Mahen H, Himle JA, Fedock G, *et al.* A pilot randomized controlled trial of cognitive behavioral therapy for perinatal depression adapted for women with low incomes. *Depress Anxiety* 2013;**30**:679–87. doi:http://0-dx.doi.org.lib.exeter.ac.uk/10.1002/da.22050

For peer review only - http://bmjopen.bmj.com/site/about/guidelines.xhtml

34 Van Lieshout RJ, Yang L, Haber E, *et al.* Evaluating the effectiveness of a brief group cognitive behavioural therapy intervention for perinatal depression. *Arch Womens Ment Health* 2017;**20**:225–8. doi:10.1007/s00737-016-0666-9

35 Lau Y, Htun TP, Wong SN, *et al.* Therapist-supported internet-based cognitive behavior therapy for stress, anxiety, and depressive symptoms among postpartum women: A systematic review and meta-analysis. *J Med Internet Res* 2017;**19**:1–17. doi:10.2196/jmir.6712

36 Biaggi A, Conroy S, Pawlby S, *et al.* Identifying the women at risk of antenatal anxiety and depression: A systematic review. J. Affect. Disord. 2016;**191**:62–77. doi:10.1016/j.jad.2015.11.014

37 Barker ED, Jaffee SR, Uher R, *et al.* The contribution of prenatal and postnatal maternal anxiety and depression to child maladjustment. *Depress Anxiety* 2011;**28**.

38 Dunkel Schetter C, Tanner L. Anxiety, depression and stress in pregnancy: Implications for mothers, children, research, and practice. Curr. Opin. Psychiatry. 2012;**25**:141–8. doi:10.1097/YCO.0b013e3283503680

39 Mills-Koonce WR, Appleyard K, Barnett M, *et al.* Adult attachment style and stress as risk factors for early maternal sensitivity and negativity. *Infant Ment Health J* 2011;**32**:277–85. doi:10.1002/imhj.20296

40 O'Hara MW. Postpartum depression: what we know. *J Clin Psychol* 2009;**65**:1258–69. doi:10.1002/jclp.20644

41 Stein A, Pearson RM, Goodman SH, *et al.* Effects of perinatal mental disorders on the fetus and child. *Lancet (London, England)* 2014;**384**:1800–19. doi:10.1016/S0140-6736(14)61277-0

42 Takács L, Smolík F, Putnam S. Assessing longitudinal pathways between maternal depressive symptoms, parenting self-esteem and infant temperament. *PLoS One* 2019;**14**:e0220633. doi:10.1371/journal.pone.0220633

43 Tambag H, Turan Z, Tolun S, *et al.* Perceived social support and depression levels of women in the postpartum period in Hatay, Turkey. *Niger J Clin Pract* 2018;**21**:1525–30. doi:10.4103/njcp.njcp_285_17

44 Murray L, Cooper P, Fearon P. Parenting difficulties and postnatal depression: Implications for primary healthcare assessment and intervention. *Community Pract* 2014;**87**:34–8.

45 Cox EQ, Sowa NA, Meltzer-Brody SE, *et al.* The perinatal depression treatment cascade. *J Clin Psychiatry* 2016;**77**:1189–200. doi:10.4088/JCP.15r10174

46 O'Mahen H, Fedock G, Henshaw E, *et al.* Modifying CBT for perinatal depression: what do women want?: a qualitative study. *Cogn Behav Pract* 2012;**19**:359–71. doi:10.1016/J.CBPRA.2011.05.005

47 Davis K, Minckas N, Bond V, *et al.* Beyond interviews and focus groups : a framework for integrating innovative qualitative methods into randomised controlled trials of complex public health interventions. 2019;**20**:1–16.

48 Moher D, Shamseer L, Clarke M, *et al.* Preferred reporting items for systematic review and meta-analysis protocols (PRISMA-P). *Syst Rev* 2015;**4**.

49 Higgins J., Green S. Cochrane handbook for systematic reviews of interventions version 5.1.0. *Cochrane Collab* 2011.

50 Moher D, Liberati A, Tetzlaff J, *et al.* Preferred reporting items for systematic reviews and meta-analyses: the PRISMA statement. *PLoS Med* 2009;**6**:e1000097.

For peer review only - http://bmjopen.bmj.com/site/about/guidelines.xhtml

doi:10.1371/journal.pmed.1000097
51 Craig P, Dieppe P, Macintyre S, *et al.* Developing and evaluating complex interventions: The new Medical Research Council guidance. *Bmj* 2008;**337**:979–83. doi:10.1136/bmj.a1655
52 Baum F, MacDougall C, Smith D. Participatory action research. *J Epidemiol Community Health* 2006;**60**:854–7. doi:10.1136/jech.2004.028662
53 Costantino G, Montano N, Casazza G. When should we change our clinical practice based on the results of a clinical study? Searching for evidence: PICOS and PubMed. *Intern Emerg Med* 2015;**10**:525–7. doi:10.1007/s11739-015-1225-5
54 European Union Agency for Fundamental Rights. Age of majority. https://fra.europa.eu/en/publication/2017/mapping-minimum-age-requirements/age-majority (accessed 10 Oct 2019).
55 US Legal. Age of Majority. https://minors.uslegal.com/age-of-majority/ (accessed 10 Oct 2019).
56 American Psychiatric Association. *Diagnostic and statistical manual of mental disorders 4th edition : DSM-IV*. 1994.
57 Cox JL, Holden JM, Sagovsky R. Detection of postnatal depression. Development of the 10-item Edinburgh Postnatal Depression Scale. *Br J Psychiatry J Ment Sci* 1987;**150**:782–6.http://www.ncbi.nlm.nih.gov/pubmed/3651732
58 Hopko DR, Lejuez CW, Ruggiero KJ, *et al.* Contemporary behavioral activation treatments for depression: procedures, principles, and progress. *Clin Psychol Rev* 2003;**23**:699–717. doi:10.1016/S0272-7358(03)00070-9
59 Mohr DC, Spring B, Freedland KE, *et al.* The selection and design of control conditions for randomized controlled trials of psychological interventions. *Psychother Psychosom* 2009;**78**:275–84. doi:10.1159/000228248
60 Evans SR. Fundamentals of clinical trial design. *J Exp Stroke Transl Med* 2010;**3**:19–27.https://www.ncbi.nlm.nih.gov/pmc/articles/PMC3083073/pdf/nihms275041.pdf (accessed 28 Nov 2018).
61 Pluye P, Nha Hong Q. Combining the power of stories and the power of numbers: mixed methods research and mixed studies reviews. *Annu Rev Public Health* 2014;**35**:29–45. doi:10.1146/annurev-publhealth-032013-182440
62 Beck AT, Steer RA, Brown GK. Manual for the Beck Depression Inventory. *San Antonio, TX Psychol Corp* 1996.
63 Fitzpatrick R, Davey C, Buxton MJ, *et al.* Evaluating patient-based outcome measures for use in clinical trials HTA Health Technology Assessment NHS R&D HTA Programme. *Health Technol Assess (Rockv)* 1998;**2**.
64 First M, Williams J, Karg R, *et al.* Structured Clinical Interview for DSM-5 (SCID-5 for DSM-5, Research Version; SCID-5-RV). *Am Psychiatr Assoc*
65 Sheehan D V., Lecrubier Y, Sheehan KH, *et al.* The Mini-International Neuropsychiatric Interview (M.I.N.I.): The development and validation of a structured diagnostic psychiatric interview for DSM-IV and ICD-10. In: *Journal of Clinical Psychiatry*. 1998. 22–33.
66 Pettersson A, Boström KB, Gustavsson P, *et al.* Which instruments to support diagnosis of depression have sufficient accuracy? A systematic review. *Nord J Psychiatry* 2015;**69**:497–508. doi:10.3109/08039488.2015.1008568
67 Beck AT, Epstein N, Brown G, *et al.* An inventory for measuring clinical anxiety:

For peer review only - http://bmjopen.bmj.com/site/about/guidelines.xhtml

psychometric properties. *J Consult Clin Psychol* 1988;**56**:893–7.http://www.ncbi.nlm.nih.gov/pubmed/3204199 (accessed 31 Jul 2018).
68 Cohen S, Kamarck T, Mermelstein R. A global measure of perceived stress. *J Health Soc Behav* 1983;**24**:385–96.
69 Abidin RR. Parenting stress index. *Psychol Assess Resour* 2012.
70 Brockington IF, Fraser C, Wilson D. The Postpartum Bonding Questionnaire: a validation. *Arch Womens Ment Health* 2006;**9**:233–42. doi:10.1007/s00737-006-0132-1
71 Zimet GD, Dahlem NW, Zimet SG, *et al.* The multidimensional scale of perceived social support. *J Pers Assess* 1988;**52**:30–41. doi:10.1207/s15327752jpa5201_2
72 Rogers H, Matthews J. The parenting sense of competence scale: Investigation of the factor structure, reliability, and validity for an Australian sample. *Aust Psychol* 2004;**39**:88–96. doi:10.1080/00050060410001660380
73 Meins E, Fernyhough C. Mind-mindedness coding manual, Version 2.0 . 2015;:1–22.
74 Milosevic I, Levy HC, Alcolado GM, *et al.* The treatment acceptability/adherence scale: moving beyond the assessment of treatment effectiveness. *Cogn Behav Ther* 2015;**44**:456–69. doi:10.1080/16506073.2015.1053407
75 Hariton E, Locascio JJ. Randomised controlled trials - the gold standard for effectiveness research: Study design: randomised controlled trials. *BJOG* 2018;**125**:1716. doi:10.1111/1471-0528.15199
76 Moher D, Hopewell S, Schulz KF, *et al.* CONSORT 2010 explanation and elaboration: Updated guidelines for reporting parallel group randomised trials. *Int J Surg* 2012;**10**:28–55. doi:10.1016/j.ijsu.2011.10.001
77 Dechartres A, Trinquart L, Faber T, *et al.* Empirical evaluation of which trial characteristics are associated with treatment effect estimates. *J Clin Epidemiol* 2016;**77**:24–37. doi:10.1016/j.jclinepi.2016.04.005
78 Higgins JP, Savović J, Page MJ, *et al.* Revised Cochrane risk-of-bias tool for randomized trials (RoB 2). 9 October 2018. *Cochrane Database Syst Rev* Published Online First: 2018. doi:10.1002/14651858.CD201601
79 Gellatly J, Bower P, Hennessy S, *et al.* What makes self-help interventions effective in the management of depressive symptoms? Meta-analysis and meta-regression. Psychol. Med. 2007;**37**:1217–28. doi:10.1017/S0033291707000062
80 Cuijpers P, van Straten A, Bohlmeijer E, *et al.* The effects of psychotherapy for adult depression are overestimated: a meta-analysis of study quality and effect size. *Psychol Med* 2010;**40**:211–23. doi:10.1017/S0033291709006114
81 Farrand P, Woodford J. Impact of support on the effectiveness of written cognitive behavioural self-help: A systematic review and meta-analysis of randomised controlled trials. *Clin Psychol Rev* 2013;**33**:182–95. doi:10.1016/j.cpr.2012.11.001
82 Farrand P, Woodford J. Effectiveness of cognitive behavioural self-help for the treatment of depression and anxiety in people with long-term physical health conditions: a systematic review and meta-analysis of randomised controlled trials. *Ann Behav Med A Publ Soc Behav Med* 2015;**49**:579–93. doi:10.1007/s12160-015-9689-0
83 Woodford J, Farrand P, Richards D, *et al.* Psychological treatments for common mental health problems experienced by informal carers of adults with chronic physical health conditions (Protocol). *Syst Rev* 2013;**2**:9. doi:10.1186/2046-4053-2-9
84 Mcgowan J, Sampson M, Salzwedel DM, *et al.* PRESS peer review explanation and strategies: 2015 guideline of electronic search elaboration. 2016.

For peer review only - http://bmjopen.bmj.com/site/about/guidelines.xhtml

85 Cooper C, Booth A, Varley-Campbell J, *et al.* Defining the process to literature searching in systematic reviews: A literature review of guidance and supporting studies. BMC Med. Res. Methodol. 2018;**18**. doi:10.1186/s12874-018-0545-3

86 Robinson KA, Whitlock EP, Oneil ME, *et al.* Integration of existing systematic reviews into new reviews: identification of guidance needs. *Syst Rev* 2014;**3**:60. doi:10.1186/2046-4053-3-60

87 Farrand P, Matthews J, Dickens C, *et al.* Psychological interventions to improve psychological well-being in people with dementia or mild cognitive impairment: systematic review and meta-analysis protocol. *BMJ Open* 2016;**6**:e009713. doi:10.1136/bmjopen-2015-009713

88 Centre for Reviews and Dissemination (CRD). Systematic reviews: CRD's guidance for undertaking reviews in health care. 2009. doi:10.1016/S1473-3099(10)70065-7

89 International Q. NVvio 10 for Windows. 2014.

90 Tricco AC, Antony J, Soobiah C, *et al.* Knowledge synthesis methods for integrating qualitative and quantitative data: a scoping review reveals poor operationalization of the methodological steps. *J Clin Epidemiol* 2016;**73**:29–35. doi:10.1016/j.jclinepi.2015.12.011

91 Ekers D, Webster L, Van Straten A, *et al.* Behavioural activation for depression; An update of meta-analysis of effectiveness and sub group analysis. *PLoS One* 2014;**9**. doi:10.1371/journal.pone.0100100

92 Flückiger C, Del Re AC, Munder T, *et al.* Enduring effects of evidence-based psychotherapies in acute depression and anxiety disorders versus treatment as usual at follow-up - A longitudinal meta-analysis. Clin. Psychol. Rev. 2014;**34**:367–75. doi:10.1016/j.cpr.2014.05.001

93 Simblett S, Birch J, Matcham F, *et al.* A systematic review and meta-analysis of e-mental health interventions to treat symptoms of posttraumatic stress. *JMIR Ment Heal* 2017;**4**:e14. doi:10.2196/mental.5558

94 Gupta S. Intention-to-treat concept: A review. *Perspect Clin Res* 2011;**2**:109. doi:10.4103/2229-3485.83221

95 Higgins JPT, Thompson SG. Quantifying heterogeneity in a meta-analysis. *Stat Med* 2002;**21**:1539–58. doi:10.1002/sim.1186

96 Higgins JPT, Thompson SG, Deeks JJ, *et al.* Measuring inconsistency in meta-analyses. *BMJ Br Med J* 2003;**327**:557–60. doi:10.1136/bmj.327.7414.557

97 Willis BH, Riley RD. Measuring the statistical validity of summary meta-analysis and meta-regression results for use in clinical practice. *Stat Med* 2017;**36**:3283–301. doi:10.1002/sim.7372

98 Greco T, Zangrillo A, Biondi-Zoccai G, *et al.* Meta-analysis: pitfalls and hints. *Hear lung Vessel* 2013;**5**:219–25. doi:10.4028/www.scientific.net/AMR.60-61.110

99 Nunan D, Aronson J, Bankhead C. Catalogue of bias: attrition bias. *BMJ evidence-based Med* 2018;**23**:21–2. doi:10.1136/ebmed-2017-110883

100 Egger M, Davey Smith G, Schneider M, *et al.* Bias in meta-analysis detected by a simple, graphical test. *BMJ* 1997;**315**:629–34. doi:10.1136/BMJ.315.7109.629

101 Sterne JA, Gavaghan D, Egger M. Publication and related bias in meta-analysis: power of statistical tests and prevalence in the literature. *J Clin Epidemiol* 2000;**53**:1119–29.http://www.ncbi.nlm.nih.gov/pubmed/11106885 (accessed 22 Feb 2019).

102 Duval S, Tweedie R. Trim and Fill: A Simple Funnel-Plot-Based Method. 2000;:455–63.

For peer review only - http://bmjopen.bmj.com/site/about/guidelines.xhtml

103 Mohr DC, Ho J, Hart TL, *et al.* Control condition design and implementation features in controlled trials: a meta-analysis of trials evaluating psychotherapy for depression. *Transl Behav Med* 2014;**4**:407–23. doi:10.1007/s13142-014-0262-3
104 Tooten A, Hoffenkamp HN, Hall RAS, *et al.* The effectiveness of video interaction guidance in parents of premature infants: A multicenter randomised controlled trial. *BMC Pediatr* 2012;**12**:1–9. doi:10.1186/1471-2431-12-76
105 Thomas J, Harden A. Methods for the thematic synthesis of qualitative research in systematic reviews. *BMC Med Res Methodol* 2008;**8**:45. doi:10.1186/1471-2288-8-45
106 Vergnes JN, Marchal-Sixou C, Nabet C, *et al.* Ethics in systematic reviews. *J Med Ethics* 2010;**36**:771–4. doi:10.1136/jme.2010.039941

LIST OF ABBREVIATIONS

BA: Behavioural Activation
CBT: Cognitive Behavioural Therapy
CRD: Centre for Reviews and Dissemination
EPDS: Edinburgh Postnatal Depression Scale
DSM: Diagnostic and Statistical Manual of Mental Disorders
IPT: Interpersonal Therapy
MeSH: Medical Subject Headings
MRC: Medical Research Council
PDSS: Postpartum Depression Screening Scale
PPD: Peripartum Depression
RCT: Randomised controlled trial
TAU: Treatment as Usual

AUTHORS' CONTRIBUTIONS

DP, JW and HO conceptualised the study. DP drafted the protocol. DP, JW, HO, and ASS designed the study. DP, JW, and HO assisted with manuscript writing. LvE, CA and OB participated critical revision of the study design and manuscript. All authors read and approved the final manuscript.

ACKNOWLEDGEMENTS

Professor Alkistis Skalkidou from the Department of Women's and Children's Health at Uppsala University for providing peer review of the search strategy.

For peer review only - http://bmjopen.bmj.com/site/about/guidelines.xhtml

Agnes Kotka, Librarian at Uppsala University Library for assisting with the development of the electronic search strategy.

FUNDING STATEMENT

This work was supported by U-CARE, which is a strategic research environment funded by the Swedish Research Council (dnr 2009–1093). This funding source had no role in the design of this study and will not have any role during its execution, analyses, interpretation of the data or decision to submit results.

COMPETING INTERESTS STATEMENT

The authors declare that they have no competing interests.

For peer review only

For peer review only - http://bmjopen.bmj.com/site/about/guidelines.xhtml

PRISMA-P (Preferred Reporting Items for Systematic review and Meta-Analysis Protocols) 2015 checklist: recommended items to address in a systematic review protocol*

Section and topic	Item No	Checklist item	(Page No.#)
**ADMINISTRATIVE INFORMATION**			
Title:			
Identification	1a	Identify the report as a protocol of a systematic review	1
Update	1b	If the protocol is for an update of a previous systematic review, identify as such	n/a
Registration	2	If registered, provide the name of the registry (such as PROSPERO) and registration number	9 (application made awaiting response)
Authors:			
Contact	3a	Provide name, institutional affiliation, e-mail address of all protocol authors; provide physical mailing address of corresponding author	1
Contributions	3b	Describe contributions of protocol authors and identify the guarantor of the review	31
Amendments	4	If the protocol represents an amendment of a previously completed or published protocol, identify as such and list changes; otherwise, state plan for documenting important protocol amendments	9
Support:			
Sources	5a	Indicate sources of financial or other support for the review	32
Sponsor	5b	Provide name for the review funder and/or sponsor	
Role of sponsor or funder	5c	Describe roles of funder(s), sponsor(s), and/or institution(s), if any, in developing the protocol	
**INTRODUCTION**			
Rationale	6	Describe the rationale for the review in the context of what is already known	4-8
Objectives	7	Provide an explicit statement of the question(s) the review will address with reference to participants, interventions, comparators, and outcomes (PICO)	9-13
**METHODS**			
Eligibility criteria	8	Specify the study characteristics (such as PICO, study design, setting, time frame) and report characteristics (such as years considered, language, publication status) to be used as criteria for eligibility for the review	9-13
Information sources	9	Describe all intended information sources (such as electronic databases, contact with study authors, trial registers or other grey literature sources) with planned dates of coverage	13-15

For peer review only

Search strategy	10	Present draft of search strategy to be used for at least one electronic database, including planned limits, such that it could be repeated	14 and online supplementary appendix 3).
Study records:			
Data management	11a	Describe the mechanism(s) that will be used to manage records and data throughout the review	19
Selection process	11b	State the process that will be used for selecting studies (such as two independent reviewers) through each phase of the review (that is, screening, eligibility and inclusion in meta-analysis)	16-18
Data collection process	11c	Describe planned method of extracting data from reports (such as piloting forms, done independently, in duplicate), any processes for obtaining and confirming data from investigators	16-18 and online supplementary appendix 5
Data items	12	List and define all variables for which data will be sought (such as PICO items, funding sources), any pre-planned data assumptions and simplifications	16-18 and online supplementary appendix 5
Outcomes and prioritization	13	List and define all outcomes for which data will be sought, including prioritization of main and additional outcomes, with rationale	11-12
Risk of bias in individual studies	14	Describe anticipated methods for assessing risk of bias of individual studies, including whether this will be done at the outcome or study level, or both; state how this information will be used in data synthesis	13,18-19and online supplementary appendix 2
Data synthesis	15a	Describe criteria under which study data will be quantitatively synthesised	19 and 20
	15b	If data are appropriate for quantitative synthesis, describe planned summary measures, methods of handling data and methods of combining data from studies, including any planned exploration of consistency (such as I^2, Kendall's τ)	19 and 20
	15c	Describe any proposed additional analyses (such as sensitivity or subgroup analyses, meta-regression)	20
	15d	If quantitative synthesis is not appropriate, describe the type of summary planned	n/a
Meta-bias(es)	16	Specify any planned assessment of meta-bias(es) (such as publication bias across studies, selective reporting within studies)	21
Confidence in cumulative evidence	17	Describe how the strength of the body of evidence will be assessed (such as GRADE)	19

*** It is strongly recommended that this checklist be read in conjunction with the PRISMA-P Explanation and Elaboration (cite when available) for important clarification on the items. Amendments to a review protocol should be tracked and dated. The copyright for PRISMA-P (including checklist) is held by the PRISMA-P Group and is distributed under a Creative Commons Attribution Licence 4.0.**

From: Shamseer L, Moher D, Clarke M, Ghersi D, Liberati A, Petticrew M, Shekelle P, Stewart L, PRISMA-P Group. Preferred reporting items for systematic review and meta-analysis protocols (PRISMA-P) 2015: elaboration and explanation. BMJ. 2015 Jan 2;349(jan02 1):g7647.

For peer review only

For peer review only

PICOS STATEMENT

Table 1: PICOS statement

PICOS	Eligibility Criteria	✓
Population	Adult women (aged ≥ 16 years)	❑
	Antepartum or postpartum (up to 12 months)	❑
	Depressive disorder with peripartum onset (no limits will be placed on depression severity)<br><br>For example:<br>• A diagnosis of major depression with peripartum onset specifier in accordance with the Diagnostic and Statistical Manual of Mental Disorders (DSM) IV or V.[37,38]<br><br>• Reporting depression symptomatology with a peripartum onset using a validated tool e.g., Edinburgh Postnatal Depression Scale (EPDS).[39]	❑
	Exclusion criteria: intervention for mood disorders other than depression (e.g. bipolar affective disorder).	❑ Does not contain
	Exclusion criteria: intervention explicitly targeting the prevention of symptoms of depression in at-risk mothers in the perinatal period.<br><br>For example,<br>A study to treating current depression during the antepartum period, with an aim to prevent depression during the postpartum period would be eligible for inclusion. However, interventions explicitly targeting the prevention of depression during either the antepartum or postpartum period will be excluded.	❑ Does not contain
Intervention	Interventions explicitly targeting an improvement in peripartum depression e.g., not targeting mother-infant interaction.<br><br>*Note:* no exclusions are placed upon:<br>• Professional group supporting the intervention<br>• Clinical setting of the intervention<br>• Delivery mode (self-guided, individual or group)<br>• Support methods (internet, face-to-face or telephone)	❑
	Intervention explicitly states the use of cognitive behavioural therapy (CBT), behavioural activation (BA) and/or problem-solving.<br><br>For example:<br><br>• CBT will be defined as interventions in which the focus is modifying a client's dysfunctional thoughts on current behaviour and future functioning.[13]	❑

For peer review only

	• BA will be defined as interventions targeting reductions in behavioural avoidance and increases in positively reinforcing activities, including interventions that focus on scheduling behaviours.[40]<br>• Problem-solving interventions will be defined as a psychological intervention including the following elements: definition of personal problems, generation of multiple solutions to each problem, selection of the best solution, developing a systematic plan for this solution, and evaluating whether the solution has resolved the problem. [13]	
Comparison	Trial contains a suitable control condition<br><br>For example:<br><br>• no-treatment control<br>• wait-list control<br>• treatment-as-usual<br>• non-specific factors component control<br>• specific factors component control<br>• active comparator<br><br>*Note:* only designs that isolate the effects of CBT should be included. For example, a study comparing CBT alone versus medication alone would be excluded as it would not be possible to isolate the effect of the CBT.	❑
Outcomes	Depression or peripartum depression is the primary outcome measure (self-report, clinician or proxy administered measure of depression)	❑
	Quality of depression measure used has internal consistency and test-retest reliability with a Cronbach's alpha ≥0.70	❑
Study design	Only randomized controlled trials	❑
	Quality of randomization procedure based on Risk of Bias tool 2.0. [57]<br><br>*Exclusion criteria:* Non-random sequence generation (high risk of bias)<br><br>*Exclusion criteria:* Designs in which allocation sequence is not concealed (before participants are enrolled and assigned to interventions) (high risk of bias)	❑<br><br>❑

Overall decision		
Include	Exclude	Review
❑	❑	❑

Notes:

For peer review only

Table 2: Article allocation in Endnote

# Endnote	Allocation	Description
0	Include	Article in line with PICOS or does not detail enough information to warrant exclusion
1	Exclude	Article clearly not in line with PICOS
2	Systematic review	Article is a systematic review in line with the PICOS
3	Meta-analysis	Article is a meta-analysis in line with the PICOS
4	Review	Article is a review of relevant literature
5	Protocol	Article is a study protocol in line with PICOS
6	Qualitative	Article is a qualitative paper in line with PICOS
7	Other	Article is useful to researcher for another purpose

For peer review only

Search strategies – electronic databases

Medline via Pubmed 1996-present - Provider: United States National Library of Medicine (NLM) – Date:		
**#**	**Searches**	**Results**
**#1**	**postpartum [Title/Abstract] OR<br>post-partum [Title/Abstract] OR<br>antepartum [Title/Abstract] OR<br>ante-partum [Title/Abstract] OR<br>partum [Title/Abstract] OR<br>prepartum [Title/Abstract] OR<br>pre-partum [Title/Abstract] OR<br>intrapartum [Title/Abstract] OR<br>intra-partum [Title/Abstract] OR<br>peripartum [Title/Abstract] OR<br>peri-partum[Title/Abstract] OR<br>postnatal [Title/Abstract] OR<br>post-natal [Title/Abstract] OR<br>perinatal [Title/Abstract] OR<br>peri-natal[Title/Abstract] OR<br>antenatal [Title/Abstract] OR<br>ante-natal [Title/Abstract] OR<br>prenatal [Title/Abstract] OR<br>pre-natal [Title/Abstract] OR<br>pregnant [Title/Abstract] OR<br>pregnancy [Title/Abstract] OR<br>pregnancies [Title/Abstract] OR<br>puerper*[Title/Abstract] OR<br>maternal [Title/Abstract] OR<br>trimester [Title/Abstract] OR<br>impregnated [Title/Abstract] OR<br>gravid* [Title/Abstract] OR<br>multigravid* [Title/Abstract] OR<br>primigravid* [Title/Abstract] OR<br>parity [Title/Abstract] OR<br>obstetric [Title/Abstract] OR<br>gestation [Title/Abstract] OR<br>"in utero" [Title/Abstract] OR<br>maternity [Title/Abstract] OR<br>partus [Title/Abstract] OR<br>obstetrical [Title/Abstract]**	
**#2**	**pregnancy [Mesh] OR<br>postpartum period [Mesh]**	
**#3**	**#1 OR #2**	
**#4**	**depression [Title/Abstract] OR<br>depressed [Title/Abstract] OR<br>depressive [Title/Abstract] OR<br>"low mood" [Title/Abstract] OR<br>mood [Title/Abstract] OR**	

For peer review only

Search strategies – electronic databases

	distress [Title/Abstract] OR<br>wellbeing [Title/Abstract] OR<br>“well being” [Title/Abstract] OR<br>emotion [Title/Abstract] OR<br>emotional [Title/Abstract] OR<br>melanchol* [Title/Abstract] OR<br>affect [Title/Abstract] OR<br>affective [Title/Abstract] OR<br>dysphori* [Title/Abstract] OR<br>dysthymia [Title/Abstract] OR<br>alexithymia [Title/Abstract]	
#5	affect [MeSH Terms] OR<br>mood disorders [MeSH Terms] OR<br>depression [MeSH Terms] OR<br>depressive disorder[MeSH Terms] OR<br>affective symptoms [MeSH Terms] OR<br>affective disorder [MeSH Terms] OR<br>depression, postpartum [MeSH Terms] OR<br>prenatal Care/psychology [MeSH Terms] OR<br>perinatal Care/psychology [MeSH Terms]) OR<br>postnatal Care/psychology [MeSH Terms] OR<br>pregnancy Complications/psychology [MeSH Terms] OR<br>pregnancy Complications/therapy [MeSH Terms]	
#6	#4 OR #5	
#7	cognitive [Title/Abstract] OR<br>behaviour [Title/Abstract] OR<br>behavioural [Title/Abstract] OR<br>behaviour [Title/Abstract] OR<br>behavioural [Title/Abstract] OR<br>cognitive behavio* [Title/Abstract] OR<br>“behavioural activation” [Title/Abstract] OR<br>“behavioral activation” [Title/Abstract] OR<br>“problem solving” [Title/Abstract] OR<br>ccbt [Title/Abstract] OR<br>icbt [Title/Abstract] OR<br>“cognitive restructuring” [Title/Abstract] OR<br>“cognitive reframing” [Title/Abstract] OR<br>“activity scheduling” [Title/Abstract]	
#8	behavior therapy [MeSH Terms] OR<br>cognitive therapy [MeSH Terms] OR<br>behavior therapies, cognitive [MeSH Terms]	
#9	#7 OR #8	
#10	therapy [Title/Abstract] OR<br>therapies [Title/Abstract] OR<br>psychotherapy [Title/Abstract] OR<br>intervention [Title/Abstract] OR<br>management [Title/Abstract] OR	

For peer review only

Search strategies – electronic databases

	"program evaluation" [Title/Abstract] OR<br>program [Title/Abstract] OR<br>programs [Title/Abstract] OR<br>programme [Title/Abstract] OR<br>programmes [Title/Abstract] OR<br>group [Title/Abstract] OR<br>course [Title/Abstract] OR<br>online [Title/Abstract] OR<br>internet [Title/Abstract] OR<br>web [Title/Abstract] OR<br>"web based" [Title/Abstract] OR<br>phone [Title/Abstract] OR<br>telephone [Title/Abstract] OR<br>skype [Title/Abstract] OR<br>e-therapy [Title/Abstract] OR<br>etherapy [Title/Abstract] OR<br>"computer assisted" [Title/Abstract]OR<br>"internet intervention" [Title/Abstract] OR<br>computer [Title/Abstract] OR<br>computerised [Title/Abstract] OR<br>computerized [Title/Abstract] OR<br>mobile [Title/Abstract] OR<br>tablet [Title/Abstract] OR<br>smartphone [Title/Abstract] OR<br>"internet administered" [Title/Abstract] OR<br>"e-mental health" [Title/Abstract] OR<br>"m-mental health" [Title/Abstract] OR<br>Ehealth [Title/Abstract] OR<br>"e-health" [Title/Abstract] OR<br>"e-intervention" [Title/Abstract]	
#11	prenatal Care/methods [Mesh]<br>perinatal Care/methods [Mesh]<br>postnatal Care/methods [Mesh]	
#12	#10 OR #11	
#13	"randomized controlled trial" [Title/Abstract] OR<br>"randomized control trial" [Title/Abstract] OR<br>RCT [Title/Abstract] OR<br>controlled [Title/Abstract] OR<br>randomised [Title/Abstract] OR<br>randomized [Title/Abstract] OR<br>randomisation [Title/Abstract] OR<br>randomization [Title/Abstract] OR<br>"random assignment" [Title/Abstract] OR<br>"random allocation"[Title/Abstract] OR<br>random [Title/Abstract] OR<br>randomly [Title/Abstract] OR<br>control [Title/Abstract] OR	

For peer review only

Search strategies – electronic databases

	feasibility [Title/Abstract] OR<br>pilot [Title/Abstract] OR<br>“comparative study” [Title/Abstract] OR<br>“follow up” [Title/Abstract] OR<br>meta-analysis [Title/Abstract] OR<br>“meta analysis” [Title/Abstract] OR<br>review [Title/Abstract]	
#14	allocation, random [MeSH Terms] OR<br>randomized controlled trial [MeSH Terms] OR<br>meta analysis [MeSH Terms] OR<br>randomized controlled trials as topic [MeSH Terms] OR<br>meta analysis as topic [MeSH Terms] OR<br>review, systematic [MeSH Terms]	
#15	randomized controlled trial [Publication Type] OR systematic review [Publication Type] OR<br>controlled clinical trial [Publication Type] OR<br>meta-analysis [Publication Type]	
#16	#13 OR #14 OR #15	
#17	#3 AND #6 AND #9 AND #12 AND #16	
#17 syntax	((((((((((((((((((((((((((((((((((((((postpartum[Title/Abstract]) OR post-partum[Title/Abstract]) OR antepartum[Title/Abstract]) OR ante-partum[Title/Abstract]) OR partum[Title/Abstract]) OR prepartum[Title/Abstract]) OR pre-partum[Title/Abstract]) OR intrapartum[Title/Abstract]) OR intra-partum[Title/Abstract]) OR peripartum[Title/Abstract]) OR peri-partum[Title/Abstract]) OR postnatal[Title/Abstract]) OR post-natal[Title/Abstract]) OR perinatal[Title/Abstract]) OR peri-natal[Title/Abstract]) OR antenatal[Title/Abstract]) OR ante-natal[Title/Abstract]) OR prenatal[Title/Abstract]) OR pre-natal[Title/Abstract]) OR pregnant[Title/Abstract]) OR pregnancy[Title/Abstract]) OR pregnancies[Title/Abstract]) OR puerper*[Title/Abstract]) OR maternal[Title/Abstract]) OR trimester[Title/Abstract]) OR impregnated[Title/Abstract]) OR gravid*[Title/Abstract]) OR multigravid*[Title/Abstract]) OR primigravid*[Title/Abstract]) OR parity[Title/Abstract]) OR obstetric[Title/Abstract]) OR gestation[Title/Abstract]) OR "in utero"[Title/Abstract]) OR maternity[Title/Abstract]) OR partus[Title/Abstract]) OR obstetrical[Title/Abstract])) OR ((Pregnancy[MeSH Terms]) OR postpartum period[MeSH Terms]))) AND ((((((((((((((((((depression[Title/Abstract]) OR depressed[Title/Abstract]) OR depressive[Title/Abstract]) OR "low mood"[Title/Abstract]) OR mood[Title/Abstract]) OR distress[Title/Abstract]) OR wellbeing[Title/Abstract]) OR "well being"[Title/Abstract]) OR emotion[Title/Abstract]) OR emotional[Title/Abstract]) OR melanchol*[Title/Abstract]) OR affect[Title/Abstract]) OR affective[Title/Abstract]) OR dysphori*[Title/Abstract]) OR dysthymia[Title/Abstract]) OR	TOTAL:

Search strategies – electronic databases

	alexithymia[Title/Abstract])) OR (((((((((((affect[MeSH Terms]) OR mood disorders[MeSH Terms]) OR depression[MeSH Terms]) OR depressive disorder[MeSH Terms]) OR affective symptoms[MeSH Terms]) OR affective disorder[MeSH Terms]) OR depression, postpartum[MeSH Terms]) OR Prenatal Care/psychology[MeSH Terms]) OR Perinatal Care/psychology[MeSH Terms]) OR Postnatal Care/psychology[MeSH Terms]) OR Pregnancy Complications/psychology[MeSH Terms]) OR Pregnancy Complications/therapy[MeSH Terms]))) AND (((((((((((((((cognitive[Title/Abstract]) OR behavior[Title/Abstract]) OR behavioral[Title/Abstract]) OR behaviour[Title/Abstract]) OR behavioural[Title/Abstract]) OR cognitive behavio*[Title/Abstract]) OR "behavioural activation"[Title/Abstract]) OR "behavioral activation"[Title/Abstract]) OR "problem solving"[Title/Abstract]) OR ccbt[Title/Abstract]) OR icbt[Title/Abstract]) OR "cognitive restructuring"[Title/Abstract]) OR "cognitive reframing"[Title/Abstract]) OR "activity scheduling"[Title/Abstract])) OR (((behavior therapy[MeSH Terms]) OR cognitive therapy[MeSH Terms]) OR behavior therapies, cognitive[MeSH Terms]))) AND ((((((((((((((((((((((((((((((((((((therapy[Title/Abstract]) OR therapies[Title/Abstract]) OR psychotherapy[Title/Abstract]) OR intervention[Title/Abstract]) OR Management[Title/Abstract]) OR "program evaluation"[Title/Abstract]) OR program[Title/Abstract]) OR programs[Title/Abstract]) OR programme[Title/Abstract]) OR programmes[Title/Abstract]) OR group[Title/Abstract]) OR course[Title/Abstract]) OR online[Title/Abstract]) OR Internet[Title/Abstract]) OR web[Title/Abstract]) OR "web based"[Title/Abstract]) OR phone[Title/Abstract]) OR telephone[Title/Abstract]) OR skype[Title/Abstract]) OR e-therapy[Title/Abstract]) OR etherapy[Title/Abstract]) OR "computer assisted"[Title/Abstract]) OR "internet intervention"[Title/Abstract]) OR computer[Title/Abstract]) OR computerised[Title/Abstract]) OR computerized[Title/Abstract]) OR mobile[Title/Abstract]) OR tablet[Title/Abstract]) OR smartphone[Title/Abstract]) OR "internet administered"[Title/Abstract]) OR e-mental health[Title/Abstract]) OR m-mental health[Title/Abstract]) OR Ehealth[Title/Abstract]) OR e-health[Title/Abstract])) OR e-intervention[Title/Abstract])) OR (((Prenatal Care/methods[MeSH Terms]) OR Perinatal Care/methods[MeSH Terms]) OR Postnatal Care/methods[MeSH Terms]))) AND ((((((randomized controlled trial[Publication Type]) OR systematic review[Publication Type]) OR controlled clinical trial[Publication Type]) OR meta-analysis[Publication Type])) OR ((((((((((((((((((("randomized controlled trial"[Title/Abstract]) OR "randomized control trial"[Title/Abstract]) OR RCT[Title/Abstract]) OR controlled[Title/Abstract]) OR randomised[Title/Abstract]) OR	

For peer review only

Search strategies – electronic databases

	**randomized[Title/Abstract]) OR randomisation[Title/Abstract]) OR randomization[Title/Abstract]) OR "random assignment"[Title/Abstract]) OR "random allocation"[Title/Abstract]) OR random[Title/Abstract]) OR randomly[Title/Abstract]) OR control[Title/Abstract]) OR feasibility[Title/Abstract]) OR pilot[Title/Abstract]) OR "comparative study"[Title/Abstract]) OR "follow up"[Title/Abstract]) OR meta-analysis[Title/Abstract]) OR "meta analysis"[Title/Abstract]) OR Review[Title/Abstract])) OR ((((((allocation, random[MeSH Terms]) OR randomized controlled trial[MeSH Terms]) OR meta analysis[MeSH Terms]) OR randomized controlled trials as topic[MeSH Terms]) OR meta analysis as topic[MeSH Terms]) OR review, systematic[MeSH Terms]))**	

ISI Web of Science Core Collection (1900-present). Including Conference Proceedings Citation Index- Science (CPCI-S) -1990-present Conference Proceedings Citation Index- Social Science & Humanities (CPCI-SSH) -1990-Present via Clarivate Analytics- Date:	
**Search**	**Results**
TI=(postpartum OR post-partum OR antepartum OR ante-partum OR partum OR prepartum OR pre-partum OR intrapartum OR intra-partum OR peripartum OR peri-partum OR postnatal OR post-natal OR perinatal OR peri-natal OR antenatal OR ante-natal OR prenatal OR pre-natal OR pregnant OR pregnancy OR pregnancies OR puerper* OR maternal OR trimester OR impregnated OR gravid* OR multigravid* OR primigravid* OR parity OR obstetric OR gestation OR “in utero” OR maternity OR partus OR obstetrical) AND TI=(depression OR depressed OR depressive OR “low mood” OR mood OR distress OR wellbeing OR well-being OR emotion OR emotional OR melanchol* OR affect OR affective OR dysphori* OR dysthymia OR alexithymia) AND TI=(cognitive OR behaviour OR behavioural OR behaviour OR behavioural OR “cognitive behavio*” OR “behavioural activation” OR “behavioral activation” OR “problem solving” OR ccbt OR icbt OR “cognitive restructuring” OR “cognitive reframing” OR “activity scheduling”) AND TI=(therapy OR therapies OR psychotherapy OR intervention OR management OR “program evaluation” OR program OR programs OR programme OR programmes OR group OR course OR online OR internet OR web OR web-based OR phone OR telephone OR skype OR e-therapy OR etherapy OR “computer assisted” OR “internet intervention” OR computer OR computerised OR computerized OR mobile OR tablet OR smartphone OR “internet administered” OR “e-mental health” OR “m-mental health” OR Ehealth OR e-health OR e-intervention) AND TI=(“randomized controlled trial” OR “randomized control trial” OR RCT OR controlled OR randomised OR randomized OR randomisation OR randomization OR “random assignment” OR “random allocation” OR random OR randomly OR control OR feasibility OR pilot OR “comparative study” OR “follow up” OR meta-analysis OR “meta analysis” OR review) OR AB=(postpartum OR post-partum OR antepartum OR	

For peer review only

Search strategies – electronic databases

ante-partum OR partum OR prepartum OR pre-partum OR intrapartum OR intra-partum OR peripartum OR peri-partum OR postnatal OR post-natal OR perinatal OR peri-natal OR antenatal OR ante-natal OR prenatal OR pre-natal OR pregnant OR pregnancy OR pregnancies OR puerper* OR maternal OR trimester OR impregnated OR gravid* OR multigravid* OR primigravid* OR parity OR obstetric OR gestation OR "in utero" OR maternity OR partus OR obstetrical) AND AB=(depression OR depressed OR depressive OR "low mood" OR mood OR distress OR wellbeing OR well-being OR emotion OR emotional OR melanchol* OR affect OR affective OR dysphori* OR dysthymia OR alexithymia) AND AB=(cognitive OR behaviour OR behavioural OR behaviour OR behavioural OR "cognitive behavio*" OR "behavioural activation" OR "behavioral activation" OR "problem solving" OR ccbt OR icbt OR "cognitive restructuring" OR "cognitive reframing" OR "activity scheduling") AND AB=(therapy OR therapies OR psychotherapy OR intervention OR management OR "program evaluation" OR program OR programs OR programme OR programmes OR group OR course OR online OR internet OR web OR web-based OR phone OR telephone OR skype OR e-therapy OR etherapy OR "computer assisted" OR "internet intervention" OR computer OR computerised OR computerized OR mobile OR tablet OR smartphone OR "internet administered" OR "e-mental health" OR "m-mental health" OR Ehealth OR e-health OR e-intervention) AND AB=("randomized controlled trial" OR "randomized control trial" OR RCT OR controlled OR randomised OR randomized OR randomisation OR randomization OR "random assignment" OR "random allocation" OR random OR randomly OR control OR feasibility OR pilot OR "comparative study" OR "follow up" OR meta-analysis OR "meta analysis" OR review)<br>*Indexes=SCI-EXPANDED, SSCI, A&HCI, CPCI-S, CPCI-SSH, ESCI Timespan=All years*	

Cumulative Index to Nursing and Allied Health Literature (CINAHL) via EBSCO host - 1981-Present - Date:	
**Search**	**Results**
TI (postpartum OR post-partum OR antepartum OR ante-partum OR partum OR prepartum OR pre-partum OR intrapartum OR intra-partum OR peripartum OR peri-partum OR postnatal OR post-natal OR perinatal OR peri-natal OR antenatal OR ante-natal OR prenatal OR pre-natal OR pregnant OR pregnancy OR pregnancies OR puerper* OR maternal OR trimester OR impregnated OR gravid* OR multigravid* OR primigravid* OR parity OR obstetric OR gestation OR "in utero" OR maternity OR partus OR obstetrical OR MH "Pregnancy") AND TI (depression OR depressed OR depressive OR "low mood" OR mood OR distress OR wellbeing OR "well being" OR emotion OR emotional OR melanchol* OR affect OR affective OR dysphori* OR dysthymia OR alexithymia OR MH "Depression, Postpartum" OR MH "Depression") AND TI (cognitive OR behaviour OR behavioural OR behaviour OR behavioural OR cognitive W2 behavio* OR "behavioural activation" OR "behavioral activation" OR "problem solving" OR ccbt OR icbt OR "cognitive restructuring" OR "cognitive reframing" OR "activity scheduling" OR MH	

For peer review only

Search strategies – electronic databases

"Cognitive Therapy" OR MH "Behavior Therapy") AND TI (therapy OR therapies OR psychotherapy OR intervention OR management OR “program evaluation” OR program OR programs OR programme OR programmes OR group OR course OR online OR internet OR web OR “web based” OR phone OR telephone OR skype OR e-therapy OR etherapy OR “computer assisted” OR “internet intervention” OR computer OR computerised OR computerized OR mobile OR tablet OR smartphone OR “internet administered” OR “e-mental health” OR “m-mental health” OR Ehealth OR “e-health” OR “e-intervention”) AND TI (“randomized controlled trial” OR “randomized control trial” OR RCT OR controlled OR randomised OR randomized OR randomisation OR randomization OR “random assignment” OR “random allocation” OR random OR randomly OR control OR feasibility OR pilot OR “comparative study” OR “follow up” OR meta-analysis OR “meta analysis” OR review OR MH "Randomized Controlled Trials" OR MH "Pilot Studies" OR MH "Meta Analysis" OR MH "Systematic Review") OR AB (postpartum OR post-partum OR antepartum OR ante-partum OR partum OR prepartum OR pre-partum OR intrapartum OR intra-partum OR peripartum OR peri-partum OR postnatal OR post-natal OR perinatal OR peri-natal OR antenatal OR ante-natal OR prenatal OR pre-natal OR pregnant OR pregnancy OR pregnancies OR puerper* OR maternal OR trimester OR impregnated OR gravid* OR multigravid* OR primigravid* OR parity OR obstetric OR gestation OR “in utero” OR maternity OR partus OR obstetrical OR MH "Pregnancy") AND AB (depression OR depressed OR depressive OR “low mood” OR mood OR distress OR wellbeing OR “well being” OR emotion OR emotional OR melanchol* OR affect OR affective OR dysphori* OR dysthymia OR alexithymia OR MH "Depression, Postpartum" OR MH "Depression") AND AB (cognitive OR behaviour OR behavioural OR behaviour OR behavioural OR cognitive W2 behavio* OR “behavioural activation” OR “behavioral activation” OR “problem solving” OR ccbt OR icbt OR “cognitive restructuring” OR “cognitive reframing” OR “activity scheduling” OR MH "Cognitive Therapy" OR MH "Behavior Therapy") AND AB (therapy OR therapies OR psychotherapy OR intervention OR management OR “program evaluation” OR program OR programs OR programme OR programmes OR group OR course OR online OR internet OR web OR “web based” OR phone OR telephone OR skype OR e-therapy OR etherapy OR “computer assisted” OR “internet intervention” OR computer OR computerised OR computerized OR mobile OR tablet OR smartphone OR “internet administered” OR “e-mental health” OR “m-mental health” OR Ehealth OR “e-health” OR “e-intervention”) AND AB (“randomized controlled trial” OR “randomized control trial” OR RCT OR controlled OR randomised OR randomized OR randomisation OR randomization OR “random assignment” OR “random allocation” OR random OR randomly OR control OR feasibility OR pilot OR “comparative study” OR “follow up” OR meta-analysis OR “meta analysis” OR review OR MH "Randomized Controlled Trials" OR MH "Pilot Studies" OR MH "Meta Analysis" OR MH "Systematic Review")	

For peer review only

Search strategies – electronic databases

Cochrane Central Register of Controlled Trials (CENTRAL) 1996-present – Date:		
**#**	**Searches**	**Results**
#1	(postpartum OR post-partum OR antepartum OR ante-partum OR partum OR prepartum OR pre-partum OR intrapartum OR intra-partum OR peripartum OR peri-partum OR postnatal OR post-natal OR perinatal OR peri-natal OR antenatal OR ante-natal OR prenatal OR pre-natal OR pregnant OR pregnancy OR pregnancies OR puerper* OR maternal OR trimester OR impregnated OR gravid* OR multigravid* OR primigravid* OR parity OR obstetric OR gestation OR “in utero” OR maternity OR partus OR obstetrical):ti,ab,kw	
#2	MeSH descriptor: [Pregnancy] explode all trees	
#3	MeSH descriptor: [Postpartum Period] explode all trees	
#4	#1 OR #2 OR #3	
#5	(depression OR depressed OR depressive OR "low mood" OR mood OR distress OR wellbeing OR "well-being" OR emotion OR emotional OR melanchol* OR affect OR affective OR dysphori* OR dysthymia OR alexithymia):ti,ab,kw	
#6	MeSH descriptor: [Mood Disorders] explode all trees	
#7	MeSH descriptor: [Depression] explode all trees	
#8	MeSH descriptor: [Depressive Disorder] explode all trees	
#9	MeSH descriptor: [Affective Symptoms] explode all trees	
#10	MeSH descriptor: [Depression, Postpartum] explode all trees	
#11	#5 OR #6 OR #7 OR #8 OR #9 OR #10	
#12	(cognitive OR behaviour OR behavioural OR behaviour OR behavioural OR cognitive NEAR/2 behavio* OR “behavioural activation” OR “behavioral activation” OR “problem solving” OR ccbt OR icbt OR “cognitive restructuring” OR “cognitive reframing” OR “activity scheduling”):ti,ab,kw	
#13	MeSH descriptor: [Behavior Therapy] explode all trees	
#14	MeSH descriptor: [Cognitive Therapy] explode all trees	
#15	#12 OR #13 OR #14	
#16	(therapy OR therapies OR psychotherapy OR intervention OR management OR “program evaluation” OR program OR programs OR programme OR programmes OR group OR course OR online OR internet OR web OR “web based” OR phone OR telephone OR skype OR e-therapy OR etherapy OR “computer assisted” OR “internet intervention” OR computer OR computerised OR computerized OR mobile OR tablet OR smartphone OR	

For peer review only

Search strategies – electronic databases

	"internet administered" OR "e-mental health" OR "m-mental health" OR Ehealth OR "e-health" OR "e-intervention"):ti,ab,kw	
#17	("randomized controlled trial" OR "randomized control trial" OR RCT OR controlled OR randomised OR randomized OR randomisation OR randomization OR "random assignment" OR "random allocation" OR random OR randomly OR control OR feasibility OR pilot OR "comparative study" OR "follow up" OR meta-analysis OR "meta analysis" OR review):ti,ab,kw	
#18	MeSH descriptor: [Random Allocation] explode all trees	
#19	MeSH descriptor: [Randomized Controlled Trial] explode all trees	
#20	MeSH descriptor: [Meta-Analysis as Topic] explode all trees	
#21	MeSH descriptor: [Randomized Controlled Trials as Topic] explode all trees	
#22	#17 OR #18 OR #19 OR #20 OR #21	
#23	#4 AND #11 AND #15 AND #16 AND #22	

PROSPERO: International prospective register of systematic reviews – 2011-present Date:	
**Search**	**Results**
postpartum OR post-partum OR antepartum OR ante-partum OR partum OR prepartum OR pre-partum OR intrapartum OR intra-partum OR peripartum OR peri-partum OR postnatal OR post-natal OR perinatal OR peri-natal OR antenatal OR ante-natal OR prenatal OR pre-natal OR pregnant OR pregnancy OR pregnancies OR puerper* OR maternal OR trimester OR impregnated OR gravid* OR multigravid* OR primigravid* OR parity OR obstetric OR gestation OR in utero OR maternity OR partus OR obstetrical AND depression OR depressed OR depressive OR low mood OR mood OR distress OR wellbeing OR well-being OR emotion OR emotional OR melanchol* OR affect OR affective OR dysphori* OR dysthymia OR alexithymia AND cognitive OR behaviour OR behavioural OR behaviour OR behavioural OR cognitive behavio* OR behavioural activation OR behavioral activation OR problem solving OR ccbt OR icbt OR cognitive restructuring OR cognitive reframing OR activity scheduling AND therapy OR therapies OR psychotherapy OR intervention OR management OR program evaluation OR program OR programs OR programme OR programmes OR group OR course OR online OR internet OR web OR web-based OR phone OR telephone OR skype OR e-therapy OR etherapy OR computer-assisted OR internet intervention OR computer OR computerised OR computerized OR mobile OR tablet OR smartphone OR internet-administered OR e-mental health OR m-mental health OR Ehealth OR e-health OR e-intervention AND randomized controlled trial OR randomized control trial OR RCT OR controlled OR randomised OR randomized OR randomisation OR randomization OR random assignment OR	

For peer review only

Search strategies – electronic databases

random allocation OR random OR randomly OR control OR feasibility OR pilot OR comparative study OR follow up:HA	

Excerpta Medica database (Embase) via OVID 1947-present – Date:		
**#**	**Searches**	**Results**
#1	(postpartum OR post-partum OR antepartum OR ante-partum OR partum OR prepartum OR pre-partum OR intrapartum OR intra-partum OR peripartum OR peri-partum OR postnatal OR post-natal OR perinatal OR peri-natal OR antenatal OR ante-natal OR prenatal OR pre-natal OR pregnant OR pregnancy OR pregnancies OR puerper* OR maternal OR trimester OR impregnated OR gravid* OR multigravid* OR primigravid* OR parity OR obstetric OR gestation OR in utero OR maternity OR partus OR obstetrical).ti,ab.	
#2	exp pregnancy/	
#3	postpartum period.mp.	
#4	puerperium/	
#5	#1 OR #2 OR #3 OR #4	
#6	(depression OR depressed OR depressive OR low mood OR mood OR distress OR wellbeing OR well-being OR emotion OR emotional OR melanchol* OR affect OR affective OR dysphori* OR dysthymia OR alexithymia).ti,ab.	
#7	exp mood disorder/	
#8	exp depression/	
#9	exp postnatal depression/	
#10	#6 OR #7 OR #8 OR #9	
#11	(cognitive OR behaviour OR behavioural OR behaviour OR behavioural OR cognitive ADJ2 behavio* OR behavioural activation OR behavioral activation OR problem solving OR ccbt OR icbt OR cognitive restructuring OR cognitive reframing OR activity scheduling).ti,ab.	
#12	exp cognitive behavioral therapy/	
#13	#12 OR #13	
#16	(therapy OR therapies OR psychotherapy OR intervention OR management OR program evaluation OR program OR programs OR programme OR programmes OR group OR	

For peer review only

Search strategies – electronic databases

	course OR online OR internet OR web OR web based OR phone OR telephone OR skype OR e-therapy OR etherapy OR computer assisted OR internet intervention OR computer OR computerised OR computerized OR mobile OR tablet OR smartphone OR internet administered OR e-mental health OR m-mental health OR Ehealth OR e-health OR e-intervention).ti,ab.	
#17	(randomized controlled trial OR randomized control trial OR RCT OR controlled OR randomised OR randomized OR randomisation OR randomization OR random assignment OR random allocation OR random OR randomly OR control OR feasibility OR pilot OR comparative study OR follow up OR meta-analysis OR meta analysis OR review).ti,ab.	
#18	exp randomization/	
#19	controlled clinical trial/	
#20	exp "systematic review"/	
#22	#17 OR #18 OR #19 OR #20 OR #21	
#23	#4 AND #11 AND #15 AND #16 AND #22	

Applied Social Sciences Index & Abstracts (ASSIA) via ProQuest 1987-present – Date:	
**Search**	**Results**
AB("postpartum" OR "post-partum" OR "antepartum" OR "ante-partum" OR "partum" OR "prepartum" OR "pre-partum" OR "intrapartum" OR "intra-partum" OR "peripartum" OR "peri-partum" OR "postnatal" OR "post-natal" OR "perinatal" OR "peri-natal" OR "antenatal" OR "ante-natal" OR "prenatal" OR "pre-natal" OR "pregnant" OR "pregnancy" OR "pregnancies" OR "puerper*" OR "maternal" OR "trimester" OR "impregnated" OR "gravid*" OR "multigravid*" OR "primigravid*" OR "parity" OR "obstetric" OR "gestation" OR “in utero” OR "maternity" OR "partus" OR "obstetrical")OR TI("postpartum" OR "post-partum" OR"antepartum" OR "ante-partum" OR "partum" OR "prepartum" OR "pre-partum" OR "intrapartum" OR "intra-partum" OR "peripartum" OR "peri-partum" OR "postnatal" OR "post-natal" OR "perinatal" OR "peri-natal" OR "antenatal" OR "ante-natal" OR "prenatal" OR "pre-natal" OR "pregnant" OR "pregnancy" OR "pregnancies" OR "puerper*" OR "maternal" OR "trimester" OR "impregnated" OR "gravid*" OR "multigravid*" OR "primigravid*" OR "parity" OR "obstetric" OR "gestation" OR “in utero” OR "maternity" OR "partus" OR "obstetrical") OR MAINSUBJECT.EXACT.EXPLODE("Pregnancy") OR MAINSUBJECT.EXACT.EXPLODE("Postpartum women") AND AB("depression” OR “depressed” OR “depressive” OR “low mood” OR “mood” OR “distress” OR “wellbeing” OR “well-being” OR “emotion” OR “emotional” OR “melanchol*” OR “affect” OR “affective” OR “dysphori*” OR	

For peer review only

Search strategies – electronic databases

“dysthymia” OR “alexithymia”) OR TI("depression” OR “depressed” OR “depressive” OR “low mood” OR “mood” OR “distress” OR “wellbeing” OR “well-being” OR “emotion” OR “emotional” OR “melanchol*” OR “affect” OR “affective” OR “dysphori*” OR “dysthymia” OR “alexithymia”) AND AB(“cognitive” OR “behaviour” OR “behavioural” OR “behaviour” OR “behavioural” OR “cognitive behavio*” OR “behavioural activation” OR “behavioral activation” OR “problem solving” OR “ccbt” OR “icbt” OR “cognitive restructuring” OR “cognitive reframing” OR “activity scheduling”) OR TI(“cognitive” OR “behaviour” OR “behavioural” OR “behaviour” OR “behavioural” OR “cognitive behavio*” OR “behavioural activation” OR “behavioral activation” OR “problem solving” OR “ccbt” OR “icbt” OR “cognitive restructuring” OR “cognitive reframing” OR “activity scheduling”) OR MAINSUBJECT.EXACT.EXPLODE("Cognitive behaviour therapy") AND AB(“therapy” OR “therapies” OR “psychotherapy” OR “intervention” OR “management” OR “program evaluation” OR “program” OR “programs” OR “programme” OR “programmes” OR “group” OR “course” OR “online” OR “internet” OR “web” OR “web-based” OR “phone” OR “telephone” OR “skype” OR “e-therapy” OR “etherapy” OR “computer-assisted” OR “internet intervention” OR “computer” OR “computerised” OR “computerized” OR “mobile” OR “tablet” OR “smartphone” OR “internet-administered” OR “e-mental health” OR “m-mental health” OR “Ehealth” OR “e-health” OR “e-intervention”) OR TI(“therapy” OR “therapies” OR “psychotherapy” OR “intervention” OR “management” OR “program evaluation” OR “program” OR “programs” OR “programme” OR “programmes” OR “group” OR “course” OR “online” OR “internet” OR “web” OR “web-based” OR “phone” OR “telephone” OR “skype” OR “e-therapy” OR “etherapy” OR “computer-assisted” OR “internet intervention” OR “computer” OR “computerised” OR “computerized” OR “mobile” OR “tablet” OR “smartphone” OR “internet-administered” OR “e-mental health” OR “m-mental health” OR “Ehealth” OR “e-health” OR “e-intervention”) AND AB(“randomized controlled trial” OR “randomized control trial” OR “RCT” OR “controlled” OR “randomised” OR “randomized” OR “randomisation” OR “randomization” OR “random assignment” OR “random allocation” OR “random” OR “randomly” OR “control” OR “feasibility” OR “pilot” OR “comparative study” OR “follow up” OR “meta-analysis” OR “metaanalysis” OR “review”) OR TI(“randomized controlled trial” OR “randomized control trial” OR “RCT” OR “controlled” OR “randomised” OR “randomized” OR “randomisation” OR “randomization” OR “random assignment” OR “random allocation” OR “random” OR “randomly” OR “control” OR “feasibility” OR “pilot” OR “comparative study” OR “follow up” OR “meta-analysis” OR “metaanalysis” OR “review”) OR MAINSUBJECT.EXACT("Randomized controlled trials") OR MAINSUBJECT.EXACT.EXPLODE("Pilot studies") OR MAINSUBJECT.EXACT.EXPLODE("Followup") OR MAINSUBJECT.EXACT.EXPLODE("Meta-analysis")	

For peer review only

Search strategies – electronic databases

Psychinfo via EBSCO host -1967-present Date: check screenshot		
**#**	**Searches**	**Results**
1	(postpartum OR post-partum OR antepartum OR ante-partum OR partum OR prepartum OR pre-partum OR intrapartum OR intra-partum OR peripartum OR peri-partum OR postnatal OR post-natal OR perinatal OR peri-natal OR antenatal OR ante-natal OR prenatal OR pre-natal OR pregnant OR pregnancy OR pregnancies OR puerper* OR maternal OR trimester OR impregnated OR gravid* OR multigravid* OR primigravid* OR parity OR obstetric OR gestation OR in utero OR maternity OR partus OR obstetrical).ti,ab.	
2	*Pregnancy/	
3	1 OR 2	
4	(depression OR depressed OR depressive OR low mood OR mood OR distress OR wellbeing OR well-being OR emotion OR emotional OR melanchol* OR affect OR affective OR dysphori* OR dysthymia OR alexithymia).ti,ab.	
5	*Major Depression/	
6	*Postpartum Depression/	
7	4 OR 5 OR 6	
8	(cognitive OR behaviour OR behavioural OR behaviour OR behavioural OR cognitive behavio* OR behavioural activation OR behavioral activation OR problem solving OR ccbt OR icbt OR cognitive restructuring OR cognitive reframing OR activity scheduling).ti,ab.	
9	*Cognitive Behavior Therapy/	
10	8 OR 9	
11	(therapy OR therapies OR psychotherapy OR intervention OR management OR program evaluation OR program OR programs OR programme OR programmes OR group OR course OR online OR internet OR web OR web-based OR phone OR telephone OR skype OR e-therapy OR etherapy OR computer-assisted OR internet intervention OR computer OR computerised OR computerized OR mobile OR tablet OR smartphone OR internet-administered OR e-mental health OR m-mental health OR Ehealth OR e-health OR e-intervention).ti,ab.	
12	(randomized controlled trial OR randomized control trial OR RCT OR controlled OR randomised OR randomized OR randomisation OR randomization OR random assignment	

For peer review only

Search strategies – electronic databases

	OR random allocation OR random OR randomly OR control OR feasibility OR pilot OR comparative study OR follow up OR meta-analysis OR meta analysis OR review).ti,ab.	
13	3 AND 7 AND 10 AND 11 AND 12	

Scopus 1966-present – Date:	
**Search**	**Results**
TITLE-ABS (postpartum OR post-partum OR antepartum OR ante-partum OR partum OR prepartum OR pre-partum OR intrapartum OR intra-partum OR peripartum OR peri-partum OR postnatal OR post-natal OR perinatal OR peri-natal OR antenatal OR ante-natal OR prenatal OR pre-natal OR pregnant OR pregnancy OR pregnancies OR puerper* OR maternal OR trimester OR impregnated OR gravid* OR multigravid* OR primigravid* OR parity OR obstetric OR gestation OR "in utero" OR maternity OR partus OR obstetrical) AND TITLE-ABS (depression OR depressed OR depressive OR "low mood" OR mood OR distress OR wellbeing OR "well-being" OR emotion OR emotional OR melanchol* OR affect OR affective OR dysphori* OR dysthymia OR alexithymia) AND TITLE-ABS (cognitive OR behaviour OR behavioural OR behaviour OR behavioural OR cognitive W/2 behavio* OR "behavioural activation" OR "behavioral activation" OR "problem solving" OR ccbt OR icbt OR "cognitive restructuring" OR "cognitive reframing" OR "activity scheduling") AND TITLE-ABS (therapy OR therapies OR psychotherapy OR intervention OR management OR "program evaluation" OR program OR programs OR programme OR programmes OR group OR course OR online OR internet OR web OR "web-based" OR phone OR telephone OR skype OR "e-therapy" OR etherapy OR "computer assisted" OR "internet intervention" OR computer OR computerised OR computerized OR mobile OR tablet OR smartphone OR "internet administered" OR "e-mental health" OR "m-mental health" OR Ehealth OR "e-health" OR "e-intervention") AND TITLE-ABS ("randomized controlled trial" OR "randomized control trial" OR RCT OR controlled OR randomised OR randomized OR randomisation OR randomization OR "random assignment" OR "random allocation" OR random OR randomly OR control OR feasibility OR pilot OR "comparative study" OR "follow up" OR meta-analysis OR "meta analysis" OR review)	

Svemed+ via Karolinska Institutet - Date:	
**Search**	**Results**
(postpartum OR post-partum OR antepartum OR ante-partum OR partum OR prepartum OR pre-partum OR intrapartum OR intra-partum OR peripartum OR peri-partum OR postnatal OR post-natal OR perinatal OR peri-natal OR antenatal OR ante-natal OR prenatal OR pre-natal OR pregnant OR pregnancy OR pregnancies OR puerper* OR maternal OR trimester OR impregnated OR gravid* OR multigravid* OR primigravid* OR parity OR obstetric OR gestation OR "in utero" OR maternity OR partus OR obstetrical OR exp: “pregnancy” OR exp: postpartum period”) AND (depression OR depressed OR depressive OR	

For peer review only - http://bmjopen.bmj.com/site/about/guidelines.xhtml

Search strategies – electronic databases

"low mood" OR mood OR distress OR wellbeing OR "well-being" OR emotion OR emotional OR melanchol* OR affect OR affective OR dysphori* OR dysthymia OR alexithymia OR exp: "affect" OR exp: "mood disorders" OR exp: "depression" OR exp: "depressive disorder" OR exp: "affective symptoms" OR exp: "affective disorder" OR exp: "depression, postpartum" OR exp: "prenatal Care/psychology" OR exp: "perinatal Care/psychology" OR exp: "postnatal Care/psychology" OR exp: "pregnancy Complications/psychology" OR exp: "pregnancy Complications/therapy") AND (cognitive OR behaviour OR behavioural OR behaviour OR behavioural OR cognitive behavio* OR "behavioural activation" OR "behavioral activation" OR "problem solving" OR ccbt OR icbt OR "cognitive restructuring" OR "cognitive reframing" OR "activity scheduling" OR exp: "behavior therapy" OR exp: "cognitive therapy" OR exp: "behavior therapies, cognitive")	

For peer review only

PRESS Guideline — Search Submission & Peer Review Assessment

SEARCH SUBMISSION: *THIS SECTION TO BE FILLED IN BY THE SEARCHER*

Searcher: Danelle Pettman **Email:** Danelle.pettman@kbh.uu.se
Reviewer: Professor Alkistis Skalkidou
Date submitted: 12.11.2018 **Date requested by**: 19.11.2018

Systematic Review Title:

Effectiveness and acceptability of cognitive behavioural therapy based interventions for maternal peripartum depression: A systematic review, meta-analysis and thematic synthesis.

This search strategy is …

X	My PRIMARY (core) database strategy — First time submitting a strategy for search question and database
	My PRIMARY (core) strategy — Follow-up review NOT the first time submitting a strategy for search question and database. If this is a response to peer review, itemize the changes made to the review suggestions
	SECONDARY search strategy— First time submitting a strategy for search question and database
	SECONDARY search strategy — NOT the first time submitting a strategy for search question and database. If this is a response to peer review, itemize the changes made to the review suggestions

Database

(i.e., MEDLINE,CINAHL…): *[mandatory]*

Medline

Interface

(i.e., Ovid, EBSCO…): *[mandatory]*

Pubmed

For peer review only - http://bmjopen.bmj.com/site/about/guidelines.xhtml

Additional database searches

Once the primary search strategy is confirmed we will also search; ISI Web of Science; Cumulative Index to Nursing and Allied Health Literature (CINAHL); Cochrane Central Register of Controlled Trials (CENTRAL); Prospero; Excerpta Medica DataBase (EMBASE); Applied Social Sciences Index and Abstracts (ASSIA); Psychinfo; SCOPUS; and SweMed+

Research Question

(Describe the purpose of the search) *[mandatory]*

(1) to examine the effectiveness of CBT based interventions for PPD on symptoms of depression; (2) to examine the effectiveness of CBT based interventions for PPD on secondary outcome measures including; anxiety, stress (individual and perceived parenting stress), parenting (e.g., sensitivity/responsiveness) perceived social support and perceived parental competence; (3) to investigate clinical and methodological moderators potentially associated with effectiveness; and (4) to describe the acceptability of CBT based interventions for PPD including (but not limited to) mothers' experiences and perceptions, satisfaction, barriers and facilitators to intervention use, intervention relevance to mothers' situations and suggestions for improvements; and (5) to identify adaptations to CBT based interventions associated with acceptability.

PICO Format

(Outline the PICOs for your question — i.e., Patient, Intervention, Comparison, Outcome, and Study Design — as applicable)

**P**	Mothers from conception to 1 year postpartum suffering with depression or low mood
**I**	All variants of behavioural, cognitive and cognitive-behavioural therapy
**C**	All comparators will be considered
**O**	Measures of depression or peripartum depression
**S**	Randomised control trials

For peer review only - http://bmjopen.bmj.com/site/about/guidelines.xhtml

Inclusion Criteria

(List criteria such as age groups, study designs, etc., to be included) *[optional]*

Participants. Adult women with either a diagnosis of PDD, or reporting some level of depression symptomatology with a peripartum onset using a validated tool (e.g., Edinburgh Postnatal Depression Scale (EPDS).

Interventions. CBT, behavioural activation (BA) and problem-solving interventions explicitly targeting an improvement in peripartum depression as a primary outcome.

Comparators. (1) no-treatment control; (2) wait-list control; (3) treatment-as-usual (TAU); (4) non-specific factors component control; (5) specific factors component control; and (6) active comparator

Outcomes: Primary outcomes of interest are self-report, clinician or proxy administered standardised measurement of depression or peripartum depession. Secondary outcomes of interest are standardised measures of (1) anxiety; (2) individual stress; (3) perceived parental stress; (4) self-report parenting; (5) perceived social support; and (6) parental competence.

Study design. Only randomized control trials will be included.

Exclusion Criteria

(List criteria such as study designs, date limits, etc., to be excluded) *[optional]*

Participants. (a) the treatment of mood disorders other than depression (e.g. bipolar affective disorder); and (b) interventions focussed on prevention of maternal psychopathology in at-risk, but not currently symptomatic mothers.

Only studies in English or Swedish will be included.

Was a search filter applied?

No ■ Yes ☐

If YES, which one(s) (e.g., Cochrane RCT filter, PubMed Clinical Queries filter)? Provide the source if this is a published filter. *[mandatory if YES to previous question — textbox]*

For peer review only - http://bmjopen.bmj.com/site/about/guidelines.xhtml

Other notes or comments you feel would be useful for the peer reviewer? ***[optional]***

We had considered not including the concept 4 therapy terms, however this reduces the specificity of the search.

Please copy and paste your search strategy here, exactly as run, including the number of hits per line. ***[mandatory]***

For peer review only

Concept 1	Concept 2	Concept 3	Concept 4	Concept 5
**Perinatal**	**Depression terms**	**CBT**	**Therapy terms**	**RCT**
**Title & Abstract**<br>Postpartum 47725<br>post-partum 11003<br>antepartum 5374<br>ante-partum 423<br>partum 12020<br>prepartum 2077<br>pre-partum 329<br>intrapartum 7905<br>intra-partum 319<br>peripartum 4151<br>peri-partum 160<br>postnatal 96485<br>post-natal 6733<br>Perinatal 65465<br>peri-natal 177<br>antenatal 31533<br>ante-natal 511<br>prenatal 87822<br>pre-natal 1028<br>pregnan* 480609<br>puerper* 14147<br>maternal 231206<br>trimester 49419<br>impregnated 10808<br>gravid* 12698<br>multigravid* 1185<br>primigravid* 4766<br>parity 29634<br>obstetric 39258<br>gestation 109623<br>before birth 3679<br>in utero 25721<br>**Mesh terms**<br>Pregnancy 844017	**Title & Abstract**<br>depress* 416497<br>depressive 102129<br>low mood 621<br>mood 66046<br>distress 99553<br>wellbeing 12032<br>well-being 63718<br>emotion 33746<br>emotional 133450<br>melanchol* 2906<br>affect 589721<br>affective 51447<br>affective symptoms 1647<br>negative affect 8508<br>dysphori*5153<br>affective disorder 6830<br>**Mesh terms**<br>affect 30820<br>mood disorders 112524<br>depression 195007<br>depressive disorder 100595<br>affective symptoms 12245<br>affective disorder 112524	**Title & Abstract**<br>cognitive 306140<br>cognitive behavio* 23129<br>behavio* 1122596<br>CBT 9128<br>BA 25470<br>problem solving 16422<br>ccbt 155<br>icbt 551<br>cognitive restructuring 785<br>cognitive reframing 84<br>activity scheduling 39<br>**Mesh terms**<br>behavior therapy 67509<br>cognitive therapy 24435<br>behavior therapies, cognitive 24435	**Title & Abstract**<br>Therapy 1749047<br>Therapies 248514<br>Psychotherapy 35578<br>Intervention 515758<br>Management 983110<br>program evaluation 6170<br>program 400451<br>programs 255468<br>programme 86763<br>programmes 55208<br>group 2335915<br>course 515471<br>online 86832<br>Internet 45003<br>Web 89875<br>web-based 25325<br>Phone 18507<br>Telephone 52303<br>Skype 273<br>e-therapy 452<br>etherapy 9<br>computer-assisted 24439<br>computerized 67756<br>internet intervention* 462<br>computer* 281642<br>mobile 85365<br>tablet* 48173<br>smartphone* 6968<br>internet-administered 65<br>ementalhealth 26	**Publication type**<br>Randomized controlled trial 470367<br>systematic review 222096<br>Clinical trial 809548<br>controlled clinical trial 558223<br>meta-analysis 93273<br>**Title & Abstract**<br>Randomized controlled trial 57325<br>Randomized control trial 3210<br>RCT 18011<br>Controlled 684318<br>randomi* 557922<br>random assignment 2191<br>random allocation 1516<br>random 239928<br>randomly 299405<br>control 2340336<br>controls 775814<br>feasibility 154608<br>pilot 132357<br>comparative study 72223<br>follow up 852728<br>meta-analysis 116966<br>metaanalysis 1531<br>Review 1392398<br>**Mesh terms**<br>allocation, random 96464<br>randomized controlled trial 121644<br>meta analysis 17054<br>randomized controlled trials as topic 121644<br>meta analysis as topic 17054<br>review, systematic 0

For peer review only - http://bmjopen.bmj.com/site/about/guidelines.xhtml

postpartum period 59467<br>prenatal care 24767<br>perinatal care 8957<br>care, postnatal 5085<br>pregnancy complications 400382<br>depression, postpartum 4780			e-mental health 175<br>m-mental health 3<br>Mmentalhealth 1<br>Ehealth 2629<br>e-health 2356	
**Total for concept 1: 1230102**	**Total for concept 2: 1670493**	**Total for concept 3: 1414869**	**Total for concept 4: 6226286**	**Total for concept 5: 6135718**
				**Total for all: 3917**

Syntax:

(((((((((((((((((((((((((((((((((((((postpartum[Title/Abstract]) OR post-partum antepartum[Title/Abstract]) OR ante-partum partum[Title/Abstract]) OR prepartum[Title/Abstract]) OR pre-partum[Title/Abstract]) OR intrapartum[Title/Abstract]) OR intra-partum[Title/Abstract]) OR peripartum[Title/Abstract]) OR peri-partum[Title/Abstract]) OR postnatal[Title/Abstract]) OR post-natal[Title/Abstract]) OR Perinatal[Title/Abstract]) OR peri-natal[Title/Abstract]) OR antenatal[Title/Abstract]) OR ante-natal[Title/Abstract]) OR prenatal[Title/Abstract]) OR pre-natal[Title/Abstract]) OR pregnan*[Title/Abstract]) OR puerper[Title/Abstract]) OR puerper*[Title/Abstract]) OR maternal[Title/Abstract]) OR trimester[Title/Abstract]) OR impregnated[Title/Abstract]) OR gravid*[Title/Abstract]) OR multigravid*[Title/Abstract]) OR primigravid*[Title/Abstract]) OR parity[Title/Abstract]) OR obstetric[Title/Abstract]) OR gestation[Title/Abstract]) OR before birth[Title/Abstract]) OR in utero[Title/Abstract])) OR (((((((pregnancy[MeSH Terms]) OR postpartum period[MeSH Terms]) OR prenatal care[MeSH Terms]) OR perinatal care[MeSH Terms]) OR care, postnatal[MeSH Terms]) OR pregnancy complications[MeSH Terms]) OR depression, postpartum[MeSH Terms]))) AND (((((((((((((((((((depress*[Title/Abstract]) OR depressive[Title/Abstract]) OR low mood[Title/Abstract]) OR mood[Title/Abstract]) OR distress[Title/Abstract]) OR wellbeing[Title/Abstract]) OR well being[Title/Abstract]) OR emotion[Title/Abstract]) OR emotional[Title/Abstract]) OR melanchol*[Title/Abstract]) OR affect[Title/Abstract]) OR affective[Title/Abstract]) OR affective symptoms[Title/Abstract]) OR negative affect[Title/Abstract]) OR dysphori*[Title/Abstract]) OR affective disorder[Title/Abstract])) OR ((((((affect[MeSH Terms]) OR mood disorders[MeSH Terms]) OR depression[MeSH Terms]) OR depressive disorder[MeSH Terms]) OR affective symptoms[MeSH Terms]) OR affective disorder[MeSH Terms]))) AND (((((((((((((cognitive[Title/Abstract]) OR cognitive behavio*[Title/Abstract]) OR behavio*[Title/Abstract]) OR CBT[Title/Abstract]) OR BA[Title/Abstract]) OR problem solving[Title/Abstract]) OR ccbt[Title/Abstract]) OR icbt[Title/Abstract]) OR cognitive

For peer review only - http://bmjopen.bmj.com/site/about/guidelines.xhtml

restructuring[Title/Abstract]) OR cognitive reframing[Title/Abstract]) OR activity scheduling[Title/Abstract])) OR (((cognitive behavior therapy[MeSH Terms]) OR behavior therapy[MeSH Terms]) OR cognitive therapy[MeSH Terms]))) AND (((((((((((((((((((((((((((((((((((therapy[Title/Abstract]) OR therapies[Title/Abstract]) OR psychotherapy[Title/Abstract]) OR intervention[Title/Abstract]) OR management[Title/Abstract]) OR program evaluation[Title/Abstract]) OR program[Title/Abstract]) OR programs[Title/Abstract]) OR programme[Title/Abstract]) OR programmes[Title/Abstract]) OR group[Title/Abstract]) OR course[Title/Abstract]) OR online[Title/Abstract]) OR Internet[Title/Abstract]) OR Web[Title/Abstract]) OR web-based[Title/Abstract]) OR Phone[Title/Abstract]) OR Telephone[Title/Abstract]) OR Skype[Title/Abstract]) OR e-therapy[Title/Abstract]) OR Etherapy[Title/Abstract]) OR computer-assisted[Title/Abstract]) OR computerized[Title/Abstract]) OR internet intervention*[Title/Abstract]) OR computer*[Title/Abstract]) OR mobile[Title/Abstract]) OR tablet*[Title/Abstract]) OR smartphone*[Title/Abstract]) OR internet-administered[Title/Abstract]) OR ementalhealth[Title/Abstract]) OR e-mental health[Title/Abstract]) OR m-mental health[Title/Abstract]) OR Mmentalhealth[Title/Abstract]) OR Ehealth[Title/Abstract]) OR e-health[Title/Abstract])) AND (((((((Randomized controlled trial[Publication Type]) OR systematic review[Publication Type]) OR controlled clinical trial[Publication Type]) OR meta-analysis[Publication Type])) OR ((((((((((((((((((Randomized controlled trial[Title/Abstract]) OR Randomized control trial[Title/Abstract]) OR RCT[Title/Abstract]) OR Controlled[Title/Abstract]) OR randomi*[Title/Abstract]) OR random assignment[Title/Abstract]) OR random allocation[Title/Abstract]) OR random[Title/Abstract]) OR randomly[Title/Abstract]) OR control[Title/Abstract]) OR controls[Title/Abstract]) OR feasibility[Title/Abstract]) OR pilot[Title/Abstract]) OR comparative study[Title/Abstract]) OR follow up[Title/Abstract]) OR meta-analysis[Title/Abstract]) OR metaanalysis[Title/Abstract]) OR Review[Title/Abstract])) OR ((((((allocation, random[MeSH Terms]) OR randomized controlled trial[MeSH Terms]) OR meta analysis[MeSH Terms]) OR randomized controlled trials as topic[MeSH Terms]) OR meta analysis as topic[MeSH Terms]) OR review, systematic[MeSH Terms]))

(Add more space, as necessary.)

For peer review only - http://bmjopen.bmj.com/site/about/guidelines.xhtml

PEER REVIEW ASSESSMENT: *THIS SECTION TO BE FILLED IN BY THE REVIEWER*

Reviewer: Professor Alkistis Skalkidou
Date completed: 12/12/2018

1. TRANSLATION

A ---No revisions	☐
B --- Revision(s) suggested	☒
C --- Revision(s) required	☐

If "B" or "C," please provide an explanation or example:

Does the search strategy match the research question/PICO?
Yes
Are the search concepts clear?
Yes
Are there too many or too few PICO elements included?
Adequate- but I wonder if the algorithm will work well when you require CBT AND therapy- will it catch something that only has CBT in the text? The word CBT includes the concept of therapy
Same goes for MESH term depression, postpartum AND depression…
Are the search concepts too narrow or too broad?
Ok but sometimes they include each other, see above- Maybe this is not a problem but should be checked.
Does the search retrieve too many or too few records? (Please show number of hits per line.)
As expected
Are unconventional or complex strategies explained?
Yes

2. BOOLEAN AND PROXIMITY OPERATORS

A ---No revisions	☐
B --- Revision(s) suggested	☒
C --- Revision(s) required	☐

If "B" or "C," please provide an explanation or example:

For peer review only - http://bmjopen.bmj.com/site/about/guidelines.xhtml

Are Boolean or proximity operators used correctly?<br>Yes<br>*Is the use of nesting with brackets appropriate and effective for the search?*<br>Yes<br>*If NOT is used, is this likely to result in any unintended exclusions?*<br>-<br>*Could precision be improved by using proximity operators (eg, adjacent, near, within) or phrase searching instead of AND?*<br>Probably, should be checked<br>*Is the width of proximity operators suitable (eg, might adj5 pick up more variants than adj2)?*<br>Not used

3. SUBJECT HEADINGS

A ---No revisions	■
B --- Revision(s) suggested	☐
C --- Revision(s) required	☐

If "B" or "C," please provide an explanation or example:

Are the subject headings relevant?<br>Yes<br>*Are any subject headings too broad or too narrow?*<br>No but somethings they intercept<br>*Are subject headings exploded where necessary and vice versa?*<br>Yes<br>*Are major headings ("starring" or restrict to focus) used? If so, is there adequate justification?*<br>-<br>*Are subheadings missing?*<br>No<br>*Are subheadings attached to subject headings? (Floating subheadings may be preferred.)*<br>Are not<br>*Are floating subheadings relevant and used appropriately?*<br>Have not seen any used (Subheading [sh:noexp])<br>*Are both subject headings and terms in free text (see the following) used for each concept?*<br>Yes

For peer review only - http://bmjopen.bmj.com/site/about/guidelines.xhtml

4. TEXT WORD SEARCHING

A ---No revisions	■
B --- Revision(s)suggested	☐
C --- Revision(s) required	☐

If "B" or "C," please provide an explanation or example:

Does the search include all spelling variants in free text (eg, UK vs. US spelling)?
Yes
Does the search include all synonyms or antonyms (eg, opposites)?
Perinatal concept include: "Maternity", "Partus" and "Obstetrical"
Depression concept include: "dysthymia" and "alexithymia"
Does the search capture relevant truncation (ie, is truncation at the correct place)?
Yes
Is the truncation too broad or too narrow?
Adequate
Are acronyms or abbreviations used appropriately? Do they capture irrelevant material? Are the full terms also included?
Yes
Have the appropriate fields been searched; for example, is the choice of the text word fields (.tw.) or all fields (.af.) appropriate? Are there any other fields to be included or excluded (database specific)?
OK
Should any long strings be broken into several shorter search statements?

5. SPELLING, SYNTAX, AND LINE NUMBERS

A ---No revisions	■
B --- Revision(s)suggested	☐
C --- Revision(s) required	☐

If "B" or "C," please provide an explanation or example:

Are there any spelling errors?
I have not identified any.
Are there any errors in system syntax; for example, the use of a truncation symbol from a different search interface?
No
Are there incorrect line combinations or orphan lines (ie, lines that are not referred to in the final summation that could indicate an error in an AND or OR statement)?
No

For peer review only - http://bmjopen.bmj.com/site/about/guidelines.xhtml

6. LIMITS AND FILTERS

A ---No revisions	☒
B --- Revision(s) suggested	☐
C --- Revision(s) required	☐

If "B" or "C," please provide an explanation or example:

OVERALL EVALUATION (Note: If one or more "revision required" is noted above, the response below must be "revisions required".)

A ---No revisions	☐
B --- Revision(s) suggested	☒
C --- Revision(s) required	☐

Additional comments:

Some more comments- look at my review on ART and endometrial cancer, Cochrane, on how we looked at grey literature (additional databases).
I also thought that you have the whole perinatal period as one, and it could be very different effects of interventions for those depressed during and/or after pregnancy- I do not know if you want to already now plan for sensitivity analyses according to timing of intervention and/or maternal depression. Outcome has the same problem- at which time is depression measured? Just a thought.

For peer review only

Data Extraction Form

Reviewer	
**Study Identification Features**	
Unique Study Identifier	
Title	
Authors	
Year of publication	
Citation	
Publication type	
Journal name	
Linked papers	
Contact author information	
Country of origin	
Funding source	
Language	
**Study Characteristics**	
Aims and objectives	
Design	
Inclusion/exclusion criteria	
**Participant characteristics**	
Method of depression assessment (e.g. structured clinical interview or screening tool)	
Diagnosis of major depressive disorder (yes/no)	
Recruitment setting (clinical or community)	
Age of mother (Mean, SD)	
Age of infant (Mean, SD)	
Point of intervention (e.g., prenatal or postnatal) ***Moderator***	
Ethnicity of mother (n, %)	
Relationship status (n, %)	
Educational status (n, %)	
Employment status (n, %),	
Mothers First child (n, %)	
Average household income (n,%)	
Breastfeeding (n,%)	
Level of depression severity at baseline (if clinical cut offs available)	
Depression Chronicity years (Mean, SD)	

For peer review only

**Intervention Components**	
Type of CBT intervention (CBT, BA or problem solving) ***Moderator***	
Inclusion of social components (e.g., peer support group or involvement of partner) -Yes/No ***Moderator***	
Inclusion of parenting intervention components (e.g., video interaction guidance - Yes/No ***Moderator***	
Treatment manual (yes/no)	
Measurement of treatment adherence (yes/no)	
Method of delivery (e.g. face to face, group or internet-administered) ***Moderator***	
Treatment setting (for example, primary care, inpatient)	
Health professional delivering intervention (e.g., clinical psychologist or midwife) ***Moderator***	
Study specific training (Yes/No)	
Duration of treatment (Weeks)	
Number of sessions	
Length of sessions (Minutes)	
Maximum length of treatment sessions over treatment course (Minutes)	
Group size (Mean, SD)	
Type of control condition used (e.g., no-treatment control, wait-list control, TAU, non- specific factors component control, specific factors component control and active comparator) ***Moderator***	
**Outcome Measurements**	
Primary outcome measure for each time point collected in both intervention and control conditions. Where multiple time points are reported, a primary end point ≤6 months post-treatment will be adopted. However, outcomes for all time points reported will be examined to enable a potential moderator analysis on length of follow-up.	
Depression measure used	
Participant n	
Mean	

For peer review only

SD and/or SE	
Quality of primary outcome measure - Cronbach's alpha for internal consistency (may need original validation paper)	
Quality of primary outcome measure - Cronbach's alpha for test-retest reliability (may need original validation paper)	
Length of follow up categorical (e.g., short; post-treament-3 months, medium; 3-6 months, long; 7-11 months; extended; 12 months+) ***Moderator***	
Secondary outcome measure for each secondary outcome (anxiety, individual stress, perceived parental stress, self-report parenting, perceived social support, parental competence and observational parenting) at each time point collected in both intervention and control conditions.	
Measure used	
Participant n	
Mean	
SD and/or SE	
**Statistical Techniques**	
Power calculation	
Intention to treat (yes/no)	
Method of dealing with missing data	
Baseline comparability	
Estimates of intracluster correlation coefficients (ICC) (cluster trials)	
**Participant Flow**	
Randomised to intervention	
Randomised to control	
Lost to follow-up intervention (at each time point measured)	
Lost to follow-up control (at each time point measured)	
Analysed intervention (at each time point measured)	
Analysed control (at each time point measured)	
Attrition rate	
**Research ethics**	
Data relating to ethics (e.g., ethical approval, ethical issues highlighted)	
**Additional comments**	

For peer review only

For peer review only
